# Myokine Cathepsin B as a Key Muscle–Brain Axis Regulator Mediates Treadmill-Running-Induced Hippocampal Neurogenesis and Cognitive Improvement in Mice

**DOI:** 10.34133/research.1233

**Published:** 2026-04-23

**Authors:** Xuchang Zhou, Dongxue Wang, Huili Deng, Jianmin Guo, Xier Chen, Zhangyu Lin, Baolong Liu, Ruobing Zhao, Lu Gao, Xuan Yin, Yun Zhang, Yan Chen, Yajing Yang, Qingxian Li, Qu Shen, Jianguang Ji, Guoxin Ni

**Affiliations:** ^1^ Department of Rehabilitation Medicine, The First Affiliated Hospital of Xiamen University, School of Medicine, Xiamen University, Xiamen 361003, China.; ^2^ Faculty of Health Sciences, University of Macau, Macau SAR 999078, China.; ^3^ School of Medicine, Xiamen University, Xiamen 361102, China.; ^4^ Institute of Integrated Bioinfomedicine and Translational Science (IBTS), School of Chinese Medicine, Hong Kong Baptist University, Hong Kong 999077, China.; ^5^ School of Sports Medicine, Wuhan Sports University, Wuhan 430079, China.; ^6^ Department of Developmental Cell Biology, Key Laboratory of Cell Biology, Ministry of Public Health, China Medical University, Shenyang 110122, China.; ^7^ Department of Exercise Physiology, Beijing Sport University, Beijing 100084, China.; ^8^ Medical College, Guangxi University, Nanning 53004, China.; ^9^ College of Acupuncture-Moxibustion and Orthopedics, Hubei University of Chinese Medicine, Wuhan 430061, China.; ^10^ Department of Orthopedic Surgery, the First Affiliated Hospital of Xiamen University, School of Medicine, Xiamen University, Xiamen 361003, China.

## Abstract

This study aimed to explore the impact of treadmill running at different intensities and durations on hippocampal neurogenesis and cognitive function in mice, with a focus on the interorgan communication mechanism mediated by the extracellular vesicle (EV) cargo cathepsin B (CTSB) via the muscle–brain axis. We define the intensity of treadmill running mice based on measurements of maximum oxygen uptake. The findings from treadmill running studies at varying intensities and durations in C57BL/6J mice revealed that treadmill running improved hippocampal neurogenesis and memory in wild-type (WT) mice in an intensity-dependent manner. Omics and UK Biobank cohort analyses identified muscle-derived CTSB as a key exercise-responsive factor, whose expression may be regulated by O-linked *N*-acetylglucosaminylation. Overexpression of O-linked *N*-acetylglucosaminyltransferase (OGT) prolonged the half-life of CTSB and inhibited its ubiquitination-mediated degradation, whereas inhibition of OGT accelerated its degradation. Mechanistically, treadmill running may promote the secretion of muscle-derived CTSB into the bloodstream via EVs and its subsequent delivery to the hippocampus through activation of the OGT/CTSB signaling. In WT mice, knockdown of muscular CTSB partially reversed the treadmill-running-induced improvements in hippocampal neurogenesis and memory, while overexpression of muscular OGT further enhanced the release of muscle-derived CTSB. Moreover, in amyloid precursor protein/presenilin 1 mice, treadmill running potentially improved cognitive function, reduced amyloid-β deposition, neurofibrillary degeneration, and neuroinflammation by up-regulating muscular CTSB. Knockdown of muscular CTSB attenuated the benefits of treadmill running, while overexpression of CTSB further enhanced the exercise-induced effects. Overall, this study demonstrates that treadmill running may activate the muscular OGT/CTSB signaling axis, promoting the secretion of the myokine CTSB protein into the circulatory system via EVs and its transport to the brain, thereby improving hippocampal neurogenesis and cognitive function in both WT and amyloid precursor protein/presenilin 1 mice. These findings highlight the role of myokine CTSB as a pivotal modulator in muscle–brain axis communication mechanism, with its stability regulated by O-linked *N*-acetylglucosaminylation.

## Introduction

With the acceleration of global population aging, the decline in learning, memory, and cognitive abilities among the elderly has become increasingly prominent [1]. Epidemiological studies indicate that approximately 50 million older adults worldwide currently suffer from cognitive impairments [2]. Cognitive decline commonly serves as an early warning sign for neurodegenerative diseases, notably Alzheimer’s disease (AD), significantly affecting patients’ learning, memory, social interaction, and daily living abilities [3,4]. As a brain region critically involved in learning and memory, the hippocampus exhibits age-related reductions in volume and neuronal number, and this degenerative change is significantly correlated with cognitive decline [5,6]. Accumulated research indicates that aerobic exercise is an effective nonpharmacological intervention for improving or delaying cognitive decline. In particular, long-term moderate-intensity training can increase hippocampal volume and enhance spatial memory [7–11], suggesting that scientifically designed exercise prescriptions may help mitigate aging-related cognitive decline. However, the dose–response connection between exercise intervention and cognitive improvement, as well as the underlying molecular mechanisms, is still not fully clarified.

Skeletal muscle, recognized as the largest endocrine organ, regulates the function of distal tissues and organs through the secretion of various myokines. Research has shown that certain myokines can enter the bloodstream, traversing the blood–brain barrier (BBB), and act directly the central nervous system, affecting both neurogenesis and synaptic plasticity [12]. Myokines such as fibronectin type III domain containing 5/irisin have been preliminarily demonstrated to traverse the BBB and up-regulate hippocampal brain-derived neurotrophic factor (BDNF) levels, thereby enhancing cognitive function [13–15]. Nevertheless, it is still uncertain which myokines are pivotal in the muscle–brain axis, and whether their expression and function exhibit exercise dose dependency has yet to be untangled. Cathepsin B (CTSB) is regarded as a key candidate myokine mediating exercise–muscle–brain signaling [16,17]. Previous studies have demonstrated that running training significantly increases CTSB levels in skeletal muscle and plasma in both rodents and primates [18]. In wild-type (WT) mice, running-induced neurogenesis and spatial memory enhancement are closely associated with elevated skeletal muscle and plasma CTSB levels. The administration of exogenous CTSB promotes learning and memory function [19]. However, the mechanisms by which exercise regulates the expression of CTSB in muscle, as well as the specific forms in which CTSB is secreted, remain unclear.

O-linked *N*-acetylglucosaminylation (O-GlcNAcylation), as a profound posttranslational modification (PTM) of proteins, is dynamically regulated under both physiological and pathological conditions [20]. Abnormal regulation of O-linked *N*-acetylglucosamine (O-GlcNAc) homeostasis has been implicated in the pathogenesis of diabetes and neurodegenerative diseases [21]. Research reveals that O-GlcNAcylation can reduce ubiquitin–proteasome-mediated protein degradation by competitively inhibiting protein phosphorylation or modulating proteolytic signaling pathways, thereby enhancing protein stability [22]. Previous studies have demonstrated that treadmill running up-regulates O-GlcNAcylation levels in muscle tissue [23]. Therefore, we hypothesize that exercise-induced O-GlcNAcylation may inhibit the ubiquitination and degradation of CTSB, thereby stabilizing the protein and promoting its accumulation and secretion in muscle tissue. On the other hand, extracellular vesicles (EVs) possess the ability to stably transport bioactive molecules over long distances in bodily fluids and to cross the BBB. EVs are regarded as a natural delivery system with the ability to penetrate biological barriers, widely involving in intertissue/organ communication [24]. EVs can protect their cargo (DNA, RNA, proteins, lipids, etc.) from degradation, particularly in the bloodstream, serving as a crucial means of intertissue/organ communication. Studies have shown that EVs play roles in physiological and pathological processes such as maintaining BBB homeostasis, regulating tumor dynamics, and modulating inflammatory responses [25,26]. However, it remains unclear whether the myokine CTSB is released via EVs to regulate neurogenesis and cognitive function.

Therefore, in this study, treadmill running of varying intensities and durations were designed based on maximum oxygen uptake (VO_2_max) testing to investigate the dose–response connection between treadmill running and cognitive function in WT mice. Furthermore, we found that treadmill running up-regulates O-GlcNAcylation levels of CTSB in muscle tissue and promotes its secretion into the peripheral circulation via EVs, ultimately improving memory and cognitive function in WT mice. Importantly, this underlying mechanism was further extended to the beneficial effects of treadmill running on AD through the use of amyloid precursor protein/presenilin 1 (APP/PS1) mice. This study unravels a key molecular mechanism through which treadmill running improves cognitive function, providing a theoretical basis for exercise intervention in the treatment of neurodegenerative disorders.

## Results

### Treadmill running at different intensities improves hippocampal neurogenesis and cognitive function in WT mice

**Fig. 1.**
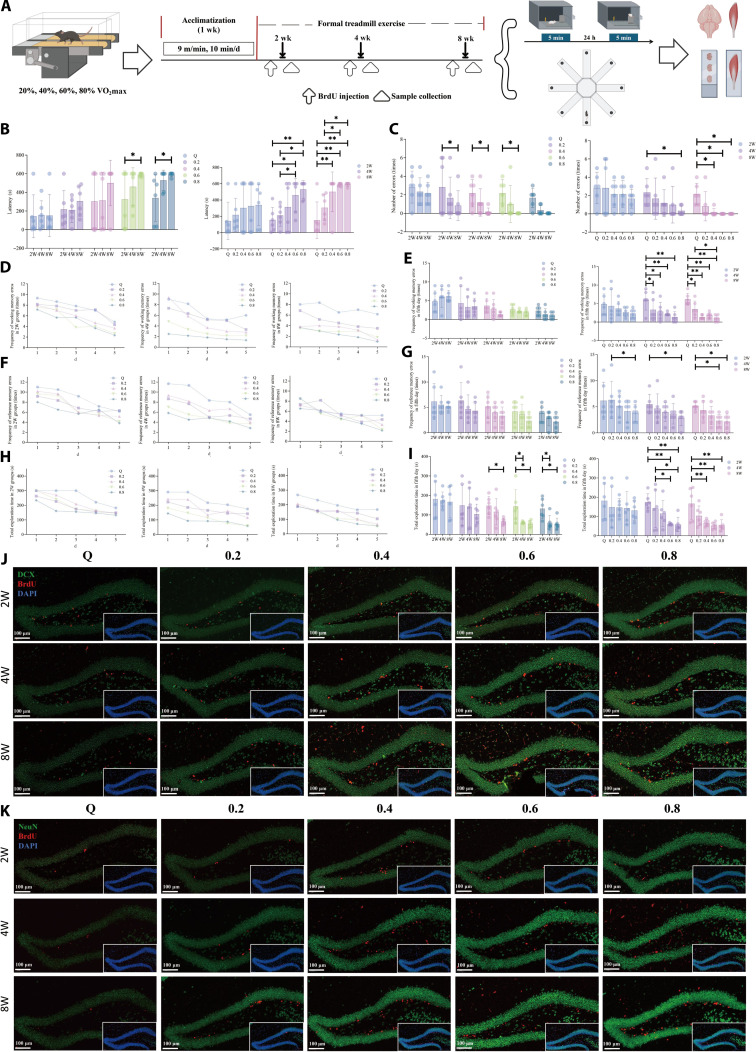
Effects of treadmill running at different intensities and durations on hippocampal neurogenesis and cognitive function in wild-type (WT) mice. (A) Schematic diagram of the animal experimental design. (B) Effects of treadmill running on the latency in the step-down avoidance test in WT mice. (C) Effects of treadmill running on the number of errors in the step-down avoidance test in WT mice. (D and E) Effects of treadmill running on the frequency of working memory errors in the 8-arm maze test in WT mice. (F and G) Effects of treadmill running on the frequency of reference memory errors in the 8-arm maze test in WT mice. (H and I) Effects of treadmill running on the total exploration time in the 8-arm maze test in WT mice. (J) Expression of 5-bromo-2′-deoxyuridine-positive (BrdU^+^)/doublecortin-positive (DCX^+^) cells in the hippocampus of WT mice. (K) Expression of BrdU^+^/neuronal-nuclear-antigen-positive (NeuN^+^) cells in the hippocampus of WT mice. 2W group, 2-week treadmill running; 4W group, 4-week treadmill running; 8W group, 8-week treadmill running; Q group, sedentary control group; 0.2 group, 20% maximum oxygen uptake (VO_2_max) treadmill running; 0.4 group, 40% VO_2_max treadmill running; 0.6 group, 60% VO_2_max treadmill running; 0.8 group, 80% VO_2_max treadmill running. *n* = 6; *P < 0.05; **P < 0.01.

As shown in Fig. 1A, VO_2_max measurements were used to define the exercise intensities for treadmill running. Based on VO_2_max values, the treadmill running groups were set at 20%, 40%, 60%, and 80% of the VO_2_max. Mice in each intensity group were further randomly divided into subgroups undergoing 2-, 4-, or 8-week treadmill running interventions. Briefly, ninety 8-week-old male C57BL/6J mice were randomly assigned to 15 groups: 2-week sedentary control group (Q2 group, *n* = 6), 2-week 20% VO_2_max treadmill running group (R2-0.2 group, *n* = 6), 2-week 40% VO_2_max treadmill running group (R2-0.4 group, *n* = 6), 2-week 60% VO_2_max treadmill running group (R2-0.6 group, *n* = 6), 2-week 80% VO_2_max treadmill running group (R2-0.8 group, *n* = 6), 4-week sedentary control group (Q4 group, *n* = 6), 4-week 20% VO_2_max treadmill running group (R4-0.2 group, *n* = 6), 4-week 40% VO_2_max treadmill running group (R4-0.4 group, *n* = 6), 4-week 60% VO_2_max treadmill running group (R4-0.6 group, *n* = 6), 4-week 80% VO_2_max treadmill running group (R4-0.8 group, *n* = 6), 8-week sedentary control group (Q8 group, *n* = 6), 8-week 20% VO_2_max treadmill running group (R8-0.2 group, *n* = 6), 8-week 40% VO_2_max treadmill running group (R8-0.4 group, *n* = 6), 8-week 60% VO_2_max treadmill running group (R8-0.6 group, *n* = 6), and 8-week 80% VO_2_max treadmill running group (R8-0.8 group, *n* = 6). All mice were subjected to the step-down avoidance test and the 8-arm maze test before euthanasia to evaluate memory and learning abilities. As shown in Fig. 1B, the latency period gradually increased with higher exercise intensity in the step-down avoidance test. Under the same exercise intensity, longer exercise duration resulted in longer latency. Furthermore, as illustrated in Fig. 1C, when exercise duration was kept constant, higher exercise intensity led to a decrease in error frequency. Similarly, under the same exercise intensity, longer exercise duration was associated with fewer errors. Results from the 8-arm maze test demonstrated that, as shown in Fig. 1D and E, under the same exercise duration, higher exercise intensity resulted in fewer working memory errors. When exercise intensity remained constant, working memory errors decreased with prolonged exercise time, although no significant differences were observed among groups. In addition, reference memory errors were influenced by exercise intensity. Under the same exercise duration, higher exercise intensity led to a reduction in reference memory errors (Fig. 1F and G). Moreover, the completion time in the 8-arm maze test was affected by both exercise intensity and exercise duration (Fig. 1H and I). Moreover, hematoxylin–eosin (HE) staining (Fig. S1A) and Nissl staining (Fig. S1B) were used to examine morphological changes in the hippocampal tissue and Nissl bodies, respectively. The results indicated that treadmill running exerted a positive effect on the histomorphology of hippocampal neurons, and this effect exhibited a dose–response relationship with both exercise intensity and duration. Furthermore, immunofluorescence staining for 5-bromo-2′-deoxyuridine (BrdU)/doublecortin (DCX) (Fig. 1J and Fig. S9A) and BrdU/neuronal nuclear antigen (NeuN) (Fig. 1K and Fig. S9B and C) was performed to assess the effect of treadmill running on hippocampal neurogenesis. The results showed that treadmill running increased the number and density of double-positive cells in the hippocampal region in a manner dependent on both exercise intensity and duration, suggesting that treadmill running may promote neuronal differentiation and maturation in the hippocampus. In summary, these findings preliminarily reveal that treadmill running can enhance hippocampal neurogenesis and memory function in WT mice in an exercise dose-dependent manner.

### Potential molecular mechanisms underlying treadmill-running-induced hippocampal neurogenesis and cognitive improvement

**Fig. 2.**
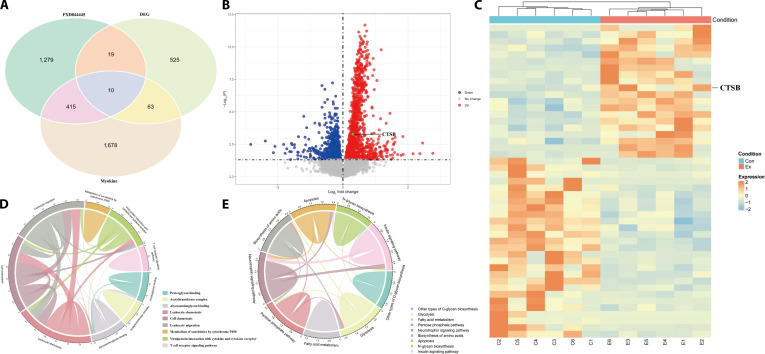
Treadmill running may promote hippocampal neurogenesis and cognitive function in wild-type (WT) mice by up-regulating the expression of the myokine cathepsin B (CTSB). (A) Venn diagram showing the overlap among differentially expressed genes (DEGs) in skeletal muscle transcriptome, differentially expressed proteins in skeletal muscle-derived extracellular vesicle (EV) proteome, and myokines. (B) Volcano plot of differentially expressed proteins in the proteome of human muscle after exercise. (C) Heatmap of DEGs in the skeletal muscle transcriptome of WT mice after 8 weeks of treadmill running at 60% maximum oxygen uptake (VO_2_max). (D) Pathway enrichment analysis of DEGs in the skeletal muscle transcriptome of WT mice following 8 weeks of treadmill running at 60% VO_2_max. (E) Pathway enrichment analysis of differentially expressed proteins in the proteome of human muscle after exercise. (B) and (E) were adapted and modified from the ProteomeXchange Consortium (https://proteomecentral.proteomexchange.org/cgi/GetDataset), and the accession numbers is ProteomeXchange: PXD044445.

**Fig. 3.**
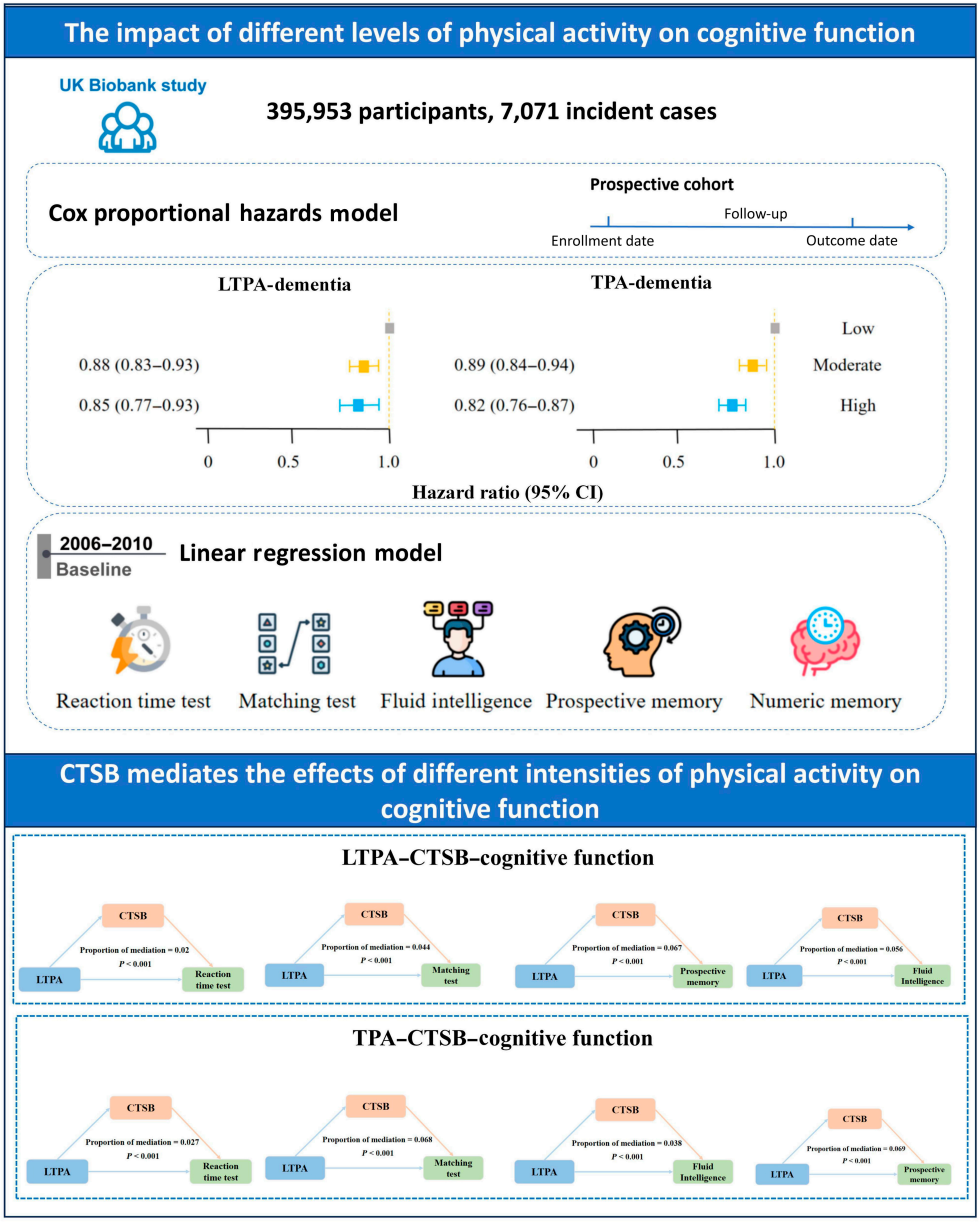
Flowchart of UK Biobank analysis. CI, confidence interval.

Although treadmill running exhibits a dose–response relationship with hippocampal neurogenesis and memory improvement in WT mice, our prior investigations indicate that high-intensity treadmill running may result in cartilage deterioration in mice [27,28]. In addition, we observed that all mice in the 80% VO_2_max group demonstrated poor compliance with the running regimen in this study. It should be emphasized that high-speed treadmill running may pose risks such as pinching injuries to the mouse’s hind legs or tail. Therefore, we believe that exercising at 60% VO_2_max intensity for 8 weeks may be the most suitable treadmill running regimen for promoting hippocampal neurogenesis and memory improvement in mice. To further investigate key secretory myokines in the muscle–brain axis, we performed transcriptomic sequencing on muscle tissue (the direct effect organ of exercise) from mice subjected to 8 weeks of treadmill running at 60% VO_2_max intensity, comparing it with muscle tissue from sedentary control mice. In addition, we retrieved one postexercise muscle proteomics dataset from the ProteomeXchange Consortium in a population study (ProteomeXchange: PXD044445). We conducted an integrative analysis of differentially expressed genes (DEGs) from muscle transcriptomics, a set of myokines, and proteomic data from human muscle after treadmill running. The results revealed 10 potential myokines (CTSB, cytoglobin, defective in cullin neddylation 1 domain containing 1 [DCUN1D1], glutaredoxin [GLRX], growth factor receptor bound protein 2, gelsolin [GSN], lysophospholipase like 1, MPS one binder kinase activator-like 1B, *N*-α-acetyltransferase 50, and serpin family E member 1 [SERPINE1]) that may function as key regulators (Fig. 2A). To further identify the key myokines, we utilized the UK Biobank database to investigate the myokines that mediate exercise-induced improvements in memory and cognition in a population-based cohort (characteristics of participants included in analyses seen in Tables 1 to 3). Our results show that higher levels of physical activity (PA), particularly moderate-intensity leisure-time PA (LTPA) and total PA (TPA), were significantly associated with a reduced incidence of dementia and enhanced cognitive performance, including reaction time, fluid intelligence, and prospective memory, although high-intensity PA showed mixed or adverse effects on cognition (Table 4). Mediation analyses revealed that CTSB (Fig. 3 and Tables 5 and 6), not other myokines (Tables 7 to 14), significantly mediated the beneficial effects of both LTPA and TPA on memory and cognitive outcomes, especially reaction time and fluid intelligence. The specific analysis process can be referred to in Fig. 3 and Supplementary Text. Furthermore, as shown in Fig. S2A to C, data retrieved from the database revealed that in a blood proteomics study involving individuals performing exercise of different intensities, a single bout of moderate-intensity exercise failed to produce a significant up-regulation of blood CTSB protein levels, whereas a single bout of high-intensity exercise elicited a clear and significant elevation [29]. This finding also suggests that the expression of CTSB protein in the blood may exhibit exercise intensity dependence in population studies. As shown in Fig. 2B and C, volcano plots of the muscle transcriptome and proteome before and after exercise demonstrate the expression of CTSB as a DEG/protein. Enrichment analysis of signaling pathways from both transcriptomic of mouse muscle (Fig. 2D) and proteomic of human muscle (Fig. 2E) data revealed that glucose metabolism and its associated O-GlcNAc modification may represent key regulatory mechanisms. Therefore, based on the findings from the aforementioned population studies and animal experiments, we hypothesize that the CTSB may act as an intermediary in the pathway through which exercise improves brain function, potentially associated with alterations in glycosylation modification levels in muscle tissue. Accordingly, in our experiments, we subsequently investigated CTSB protein levels in mouse skeletal muscle under different intensities of treadmill running (Fig. S2D). We found that treadmill running promotes CTSB expression in muscle in a dose-dependent manner. Furthermore, we observed that treadmill running elevates the protein expression of BDNF and CTSB in the mouse hippocampal tissue, with these effects being dependent on both exercise intensity and duration (Fig. S2E). Consistently, the elevations in blood BDNF and CTSB concentrations also exhibited exercise dose dependency (Fig. S2F and G). These findings imply that exercise could enhance the expression of myogenic CTSB protein to enhance hippocampal neurogenesis and cognitive function.

**Table 1.** Assigned values for frequency and duration of leisure time physical activity

	Options	Assigned values
Frequency (times/week)	Once in the last 4 weeks	0.25
	2–3 times in the last 4 weeks	0.625
	Once a week	1
	2–3 times a week	2.5
	4–5 times a week	4.5
	Every day	7
Duration (min)	Less than 15 min	7.5
	Between 15 and 30 min	22.5
	Between 30 min and 1 h	45
	Between 1 and 1.5 h	75
	Between 1.5 and 2 h	105
	Between 2 and 3 h	150
	Over 3 h	195

**Table 2.** International Classification of Disease codes used to ascertain dementia

International Classification of Diseases 10th Revision
F00, F00.0, F00.1, F00.2, F00.9, G30, G30.0, G30.1, G30.8, G30.9, F01, F01.0, F01.1, F01.2, F01.3, F01.8, F01.9, I67.3, F02.0, G31.0, A81.0, F02, F02.1, F02.2, F02.3, F02.4, F02.8, F03, F05.1, F10.6, G31.1, G31.8

**Table 3.** Characteristics of participants included in analyses

	Overall	Normal	Dementia
*N*	397,765	390,619	7,146
**Follow-up (years) (median [IQR])**	13.63 [12.89–14.29]	13.65 [12.93–14.30]	10.48 [8.03–12.29]
**Age (median [IQR])**	58.0 [51.0–64.0]	58.0 [50.0–64.0]	66.0 [63.0–68.0]
**Female (%)**	179,912 (45.2)	176,218 (45.1)	3,694 (51.7)
White	379,053 (95.3)	372,169 (95.3)	6,884 (96.3)
Asian	9,395 (2.4)	9,274 (2.4)	121 (1.7)
Black	6,757 (1.7)	6,644 (1.7)	113 (1.6)
Mixed	2,560 (0.6)	2,532 (0.6)	28 (0.4)
**Education (%)**			
College or university degree	132,399 (33.3)	130,863 (33.5)	1,536 (21.5)
A levels/AS levels or equivalent	45,269 (11.4)	44,679 (11.4)	590 (8.3)
O levels/GCSEs or equivalent	85,261 (21.4)	83,878 (21.5)	1,383 (19.4)
GCSEs or equivalent	21,635 (5.4)	21,467 (5.5)	168 (2.4)
NVQ or HND or HNC or equivalent	25,883 (6.5)	25,361 (6.5)	522 (7.3)
Other qualifications	87,318 (22.0)	84,371 (21.6)	2,947 (41.2)
**BMI (%)**			
<18.5	2,078 (0.5)	2,029 (0.5)	49 (0.7)
18.5–25	131,503 (33.1)	129,382 (33.1)	2,121 (29.7)
25–30	169,531 (42.6)	166,422 (42.6)	3,109 (43.5)
≥30	94,653 (23.8)	92,786 (23.8)	1,867 (26.1)
**LTPA categories, MET-min/week (median [IQR])**	701.3 [274.2–1,448.4]	702.2 [275.2–1,448.4]	668.7 [225.0–1,448.4]
**LTPA category (%)**			
<600	166,635 (41.9)	163,698 (41.9)	2,937 (41.1)
600–3,000	175,052 (44.0)	172,204 (44.1)	2,848 (39.9)
≥3,000	28,670 (7.2)	28,159 (7.2)	511 (7.2)
Missing	27,408 (6.9)	26,558 (6.8)	850 (11.9)
**TPA categories, MET-min/week (median [IQR])**	1,506.0 [594.0–3,217.5]	1,506.0 [594.0–3,219.0]	1,404.0 [516.0–3,199.0]
**TPA category (%)**			
<600	99,875 (25.1)	97,933 (25.1)	1,942 (27.2)
600–3,000	188,183 (47.3)	184,956 (47.3)	3,227 (45.2)
≥3,000	107,895 (27.1)	105,993 (27.1)	1,902 (26.6)
Missing	1,812 (0.5)	1,737 (0.4)	75 (1.0)
**Smoking (%)**			
Never	219,868 (55.3)	216,510 (55.4)	3,358 (47.0)
Previous	139,867 (35.2)	136,766 (35.0)	3,101 (43.4)
Current	38,030 (9.6)	37,343 (9.6)	687 (9.6)
**Alcohol (%)**			
Never	16,612 (4.2)	16,122 (4.1)	490 (6.9)
Previous	13,690 (3.4)	13,227 (3.4)	463 (6.5)
Current	367,463 (92.4)	361,270 (92.5)	6,193 (86.7)
**Family dementia (%)**			
Yes	46,960 (11.8)	45,450 (11.6)	1,510 (21.1)
No	350,805(88.2)	345,169(88.4)	5,636(78.9)
**APOEɛ4 (%)**			
APOEɛ4 carrier	103,647 (26.1)	100,021 (25.6)	3,626 (50.7)
APOEɛ4 noncarrier	294,118(73.9)	290,598(74.4)	3,520(49.3)
**Age (median [IQR])**	58.0 [51.0–64.0]	58.0 [50.0–64.0]	66.0 [63.0–68.0]
**TDI (median[IQR])**	−2.22 [−3.68–0.37]	−2.22 [−3.68–0.36]	−1.96 [−3.56–1.00]

IQR, interquartile range; A, advanced; AS, advanced subsidiary; O, ordinary; GCSEs, General Certificate of Secondary Educations; NVQs, National Vocational Qualifications; HND, Higher National Diploma; HNC, Higher National Certificate; BMI, body mass index; LTPA, leisure-time physical activity; TPA, total physical activity; MET, metabolic equivalent of task; APOEɛ4, apolipoprotein E ε4; TDI, Townsend deprivation index.

**Table 4.** Hazard ratios for dementia and cognitive decline across physical activity levels

LTPA	*N*	Model 1^a^			Model 2^b^			Model 3^c^		
		*β*	95% CI	*P* value	*aβ*	95% CI	*P* value	*aβ*	95% CI	*P* value
Dementia	370,357 (events = 6,296)									
Moderate versus low		0.92	(0.87 to 0.97)	0.001	0.85	(0.81 to 0.89)	<0.001	0.88	(0.83 to 0.93)	<0.001
High versus low		1.01	(0.92 to 1.11)	0.809	0.81	(0.74 to 0.89)	<0.001	0.85	(0.77 to 0.93)	0.001
Reaction time test	367,571									
Moderate versus low		−8.54	(−9.31 to −7.77)	<0.001	−7.25	−7.99 to −6.51	<0.001	−4.40	(−5.14 to −3.67)	<0.001
High versus low		−13.60	(−15.04 to −12.16)	<0.001	−12.09	(−13.47 to −10.7)	<0.001	−8.96	(−10.34 to −7.58)	<0.001
Matching test	370,347									
Moderate versus low		−0.06	(−0.07 to −0.06)	<0.001	−0.04	(−0.05 to −0.04)	<0.001	−0.02	(−0.03 to −0.02)	<0.001
High versus low		−0.04	(−0.05 to −0.02)	<0.001	−0.02	(−0.04 to −0.01)	0.002	0.00	(−0.02 to −0.01)	0.884
Fluid intelligence	122,849									
Moderate versus low		0.29	(0.26 to 0.31)	<0.001	0.13	(0.11 to 0.16)	<0.001	0.03	(0.01 to 0.06)	0.004
High versus low		0.01	(−0.03 to 0.06)	0.628	−0.12	(−0.16 to −0.08)	<0.001	−0.24	(−0.29 to −0.20)	<0.001
Prospective memory	126,245									
Moderate versus low		0.05	(0.04 to 0.06)	<0.001	0.04	(0.03 to 0.04)	<0.001	0.01	(0.01 to 0.02)	<0.001
High versus low		0.03	(0.02 to 0.04)	<0.001	0.03	(0.01 to 0.04)	<0.001	−0.01	(−0.02 to 0.01)	0.340
Numeric memory	38,372									
Moderate versus low		0.13	(0.1 to 0.16)	<0.001	0.05	(0.03 to 0.08)	<0.001	0.02	(−0.01 to 0.05)	0.133
High versus low		0.03	(−0.02, 0.08)	0.236	−0.06	(−0.11 to −0.02)	0.008	−0.10	(−0.15 to −0.06)	<0.001
TPA										
Dementia	395953 (events = 7,071)									
Moderate versus low		0.87	(0.83 to 0.92)	<0.001	0.87	(0.82 to 0.92)	<0.001	0.89	(0.84 to 0.94)	<0.001
High versus low		0.9	(0.85 to 0.96)	0.001	0.79	(0.75 to 0.85)	<0.001	0.82	(0.76 to 0.87)	<0.001
Reaction time test	392,710									
Moderate versus low		−6.35	(−7.25 to −5.46)	<0.001	−3.19	(−4.05 to −2.34)	<0.001	−1.82	(−2.67 to −0.96)	<0.001
High versus low		−5.76	(−6.77 to −4.76)	<0.001	−5.49	(−6.45 to −4.53)	<0.001	−4.09	(−5.05 to −3.14)	<0.001
Matching test	395,941									
Moderate versus low		−0.04	(−0.05 to −0.03)	<0.001	−0.01	(−0.02 to 0.00)	0.036	0	(−0.01 to 0.01)	0.682
High versus low		0.01	(0.00 to 0.02)	0.018	0.01	(0.00 to 0.02)	0.009	0.02	(0.01 to 0.03)	<0.001
Fluid intelligence	130,715									
Moderate versus low		0.26	(0.23 to 0.29)	<0.001	0.09	(0.06 to 0.11)	<0.001	0.05	(0.03 to 0.08)	<0.001
High versus low		−0.19	(−0.23 to −0.16)	<0.001	−0.19	(−0.22 to −0.16)	<0.001	−0.24	(−0.27 to −0.21)	<0.001
Prospective memory	134,646									
Moderate versus low		0.04	(0.03 to 0.05)	<0.001	0.02	(0.01 to 0.03)	<0.001	0.01	(0.00 to 0.02)	0.007
High versus low		0.01	(0.01 to 0.02)	0.001	0.01	(0.01 to 0.02)	0.001	0	(−0.01 to 0.00)	0.432
Numeric memory	40,211									
Moderate versus low		0.12	(0.09 to 0.15	<0.001	0.04	(0.01 to 0.08)	0.007	0.02	(−0.01 to 0.05)	0.254
High versus low		−0.07	(−0.1 to −0.03)	<0.001	−0.08	(−0.11 to −0.04)	<0.001	−0.11	(−0.15 to −0.08)	<0.001

^a^ Model 1: Crude model.

^b^ Model 2: Sex, age, education, and apolipoprotein E ε4 (APOEɛ4) were adjusted.

^c^ Model 3: Sex, age, education, APOEɛ4, ethnic, family dementia history, body mass index, Townsend deprivation index, smoking status, and alcohol status were adjusted.

CI, confidence interval.

**Table 5.** Mediation analysis of CTSB for cognitive function across LTPA levels

LTPA and CTSB	Estimate ^a^	Lower 95%CI	Upper 95% CI	*P* value
Reaction time test (*N* = 47,967)				
ACME	-0.230	−0.360	−0.106	<0.001
ADE	−11.318	−13.432	−9.072	<0.001
Total effect	−11.547	−13.678	−9.284	<0.001
Proportion of mediation (%)	0.020	0.009	0.033	<0.001
**Matching test (*N* = 48,429)**				
ACME	−0.004	−0.005	−0.002	<0.001
ADE	−0.076	−0.098	−0.053	<0.001
Total effect	−0.080	−0.102	−0.056	<0.001
Proportion of mediation (%)	0.044	0.026	0.075	<0.001
**Fluid intelligence (*N* = 15,748)**				
ACME	0.017	0.011	0.023	<0.001
ADE	0.287	0.218	0.361	<0.001
Total effect	0.304	0.236	0.377	<0.001
Proportion of mediation (%)	0.056	0.035	0.084	<0.001
**Prospective memory (*N* = 16,310)**				
ACME	0.004	0.003	0.006	<0.001
ADE	0.057	0.040	0.076	<0.001
Total effect	0.061	0.044	0.080	<0.001
Proportion of mediation (%)	0.067	0.041	0.106	<0.001
**Numeric memory (*N* = 5,203)**				
ACME	0.000	−0.004	0.004	0.944
ADE	0.126	0.051	0.208	<0.001
Total effect	0.126	0.050	0.208	0.002
Proportion of mediation (%)	0.001	−0.032	0.037	0.942

^a^ The model was adjusted for age and sex.

CTSB, cathepsin B; LTPA, leisure-time physical activity; CI, confidence interval; ACME, average causal mediation effect; ADE, average direct effect.

**Table 6.** Mediation analysis of CTSB for cognitive function across TPA levels

TPA and CTSB	Estimate ^a^	Lower 95%CI	Upper 95% CI	*P* value
Reaction time test (*N* = 52,011)				
ACME	−0.190	−0.292	−0.098	<0.001
ADE	−6.802	−9.229	−4.088	<0.001
Total effect	−6.992	−9.459	−4.295	<0.001
Prop. mediated (%)	0.027	0.013	0.052	<0.001
**Matching test (*N* = 52,323)**				
ACME	−0.003	−0.004	−0.002	<0.001
ADE	−0.037	−0.062	−0.010	0.008
Total effect	−0.040	−0.066	−0.013	0.006
Prop. mediated	0.068	0.034	0.221	0.006
**Fluid intelligence (*N* = 16,918)**				
ACME	0.010	0.004	0.016	<0.001
ADE	0.248	0.168	0.336	<0.001
Total effect	0.257	0.176	0.344	<0.001
Prop. mediated	0.038	0.015	0.070	<0.001
**Prospective memory (*N* = 17,581)**				
ACME	0.002	0.001	0.004	<0.001
ADE	0.033	0.013	0.055	0.002
Total effect	0.035	0.015	0.057	<0.001
Prop. mediated	0.069	0.029	0.190	<0.001
**Numeric memory (*N* = 5,525)**				
ACME	0.001	−0.003	0.005	0.612
ADE	0.071	−0.016	0.167	0.130
Total effect	0.072	−0.016	0.167	0.126
Prop. mediated	0.010	−0.194	0.170	0.638

^a^ The model was adjusted for age and sex.

CTSB, cathepsin B; TPA, total physical activity; CI, confidence interval; ACME, average causal mediation effect; ADE, average direct effect.

**Table 7.** Mediation analysis of DCUN1D1 for cognitive function across LTPA levels

LTPA and DCUN1D1	Estimate ^a^	Lower 95%CI	Upper 95% CI	*P* value
Reaction time test (*N* = 47,967)				
ACME	0.000	−0.035	0.033	0.994
ADE	−11.547	−13.653	−9.293	<0.001
Total effect	−11.547	−13.649	−9.280	<0.001
Prop. mediated	0.000	−0.003	0.003	0.994
**Matching test (*N* = 48,429)**				
ACME	0.000	−0.001	0.000	0.488
ADE	−0.080	−0.102	−0.056	<0.001
Total effect	−0.080	−0.102	−0.056	<0.001
Prop. mediated	0.001	−0.003	0.007	0.488
**Fluid intelligence (*N* = 15,748)**				
ACME	0.002	0.000	0.004	0.052
ADE	0.303	0.234	0.376	<0.001
Total effect	0.304	0.236	0.378	<0.001
Prop. mediated	0.005	0.000	0.013	0.052
**Prospective memory (*N* = 16,310)**				
ACME	0.000	0.000	0.001	0.024
ADE	0.061	0.043	0.079	<0.001
Total effect	0.061	0.044	0.080	<0.001
Prop. mediated	0.007	0.000	0.019	0.024
**Numeric memory (*N* = 5,203)**				
ACME	0.000	−0.003	0.002	0.700
ADE	0.127	0.052	0.208	<0.001
Total effect	0.126	0.050	0.208	<0.001
Prop. mediated	−0.003	−0.034	0.019	0.700

^a^ The model was adjusted for age and sex.

DCUN1D1, defective in cullin neddylation 1 domain containing 1; LTPA, leisure-time physical activity; CI, confidence interval; ACME, average causal mediation effect; ADE, average direct effect.

**Table 8.** Mediation analysis of DCUN1D1 for cognitive function across TPA levels

TPA and DCUN1D1	Estimate ^a^	Lower 95%CI	Upper 95% CI	*P* value
Reaction time test (*N* = 52,011)				
ACME	0.000	−0.016	0.016	0.960
ADE	−6.992	−9.421	−4.283	<0.001
Total effect	−6.992	−9.427	−4.285	<0.001
Prop. mediated	0.000	−0.002	0.002	0.960
**Matching test (*N* = 52,323)**				
ACME	0.000	0.000	0.000	0.602
ADE	−0.040	−0.065	−0.013	0.006
Total effect	−0.040	−0.065	−0.013	0.006
Prop. mediated	0.001	−0.003	0.009	0.608
**Fluid intelligence (*N* = 16,918)**				
ACME	0.001	−0.001	0.003	0.226
ADE	0.257	0.177	0.345	<0.001
Total effect	0.258	0.178	0.346	<0.001
Prop. mediated	0.003	−0.002	0.012	0.226
**Prospective memory (*N* = 17,581)**				
ACME	0.000	0.000	0.001	0.150
ADE	0.035	0.015	0.057	<0.001
Total effect	0.035	0.015	0.058	<0.001
Prop. mediated	0.009	−0.004	0.037	0.150
**Numeric memory (*N* = 5,525)**				
ACME	0.000	−0.003	0.001	0.666
ADE	0.072	−0.015	0.169	0.122
Total effect	0.072	−0.016	0.169	0.126
Prop. mediated	−0.002	−0.106	0.074	0.724

^a^ The model was adjusted for age and sex.

DCUN1D1, defective in cullin neddylation 1 domain containing 1; TPA, total physical activity; CI, confidence interval; ACME, average causal mediation effect; ADE, average direct effect.

**Table 9.** Mediation analysis of GLRX for cognitive function across LTPA levels

LTPA and GLRX	Estimate ^a^	Lower 95%CI	Upper 95% CI	*P* value
Reaction time test (*N* = 47,967)				
ACME	−0.121	−0.205	−0.055	<0.001
ADE	−11.425	−13.530	−9.154	<0.001
Total effect	−11.546	−13.625	−9.307	<0.001
Prop. mediated	0.010	0.005	0.018	<0.001
**Matching test (*N* = 48,429)**				
ACME	0.000	−0.001	0.001	0.796
ADE	−0.080	−0.102	−0.056	<0.001
Total effect	−0.080	−0.102	−0.056	<0.001
Prop. mediated	0.001	−0.008	0.010	0.796
**Fluid intelligence (*N* = 15,748)**				
ACME	0.001	−0.001	0.004	0.3
ADE	0.304	0.235	0.377	<0.001
Total effect	0.305	0.235	0.379	<0.001
Prop. mediated	0.003	−0.004	0.012	0.3
**Prospective memory (*N* = 16,310)**				
ACME	0.000	−0.001	0.001	0.688
ADE	0.061	0.044	0.080	<0.001
Total effect	0.061	0.044	0.080	<0.001
Prop. mediated	0.002	−0.008	0.013	0.688
**Numeric memory (*N* = 5,203)**				
ACME	0.003	−0.001	0.008	0.180
ADE	0.124	0.048	0.205	0.002
Total effect	0.127	0.051	0.208	<0.001
Prop. mediated	0.019	−0.011	0.080	0.180

^a^ The model was adjusted for age and sex.

GLRX, glutaredoxin; LTPA, leisure-time physical activity; CI, confidence interval; ACME, average causal mediation effect; ADE, average direct effect.

**Table 10.** Mediation analysis of GLRX for cognitive function across TPA levels

TPA and GLRX	Estimate ^a^	Lower 95%CI	Upper 95% CI	*P* value
Reaction time test (*N* = 52,011)				
ACME	−0.060	−0.124	−0.008	0.026
ADE	−6.932	−9.361	−4.227	<0.001
Total effect	−6.992	−9.444	−4.288	<0.001
Prop. mediated	0.008	0.001	0.021	0.026
**Matching test (*N* = 52,323)**				
ACME	0.000	0.000	0.000	0.678
ADE	−0.039	−0.065	−0.013	0.006
Total effect	−0.040	−0.065	−0.013	0.006
Prop. mediated	0.001	−0.011	0.017	0.684
**Fluid intelligence (*N* = 16,918)**				
ACME	0.000	−0.001	0.002	0.474
ADE	0.257	0.177	0.346	<0.001
Total effect	0.258	0.177	0.347	<0.001
Prop. mediated	0.001	−0.002	0.008	0.474
**Prospective memory (*N* = 17,581)**				
ACME	0.000	0.000	0.000	0.624
ADE	0.035	0.015	0.058	<0.001
Total effect	0.035	0.015	0.058	<0.001
Prop. mediated	0.001	−0.006	0.015	0.624
**Numeric memory (*N* = 5,525)**				
ACME	0.000	−0.002	0.002	0.998
ADE	0.072	−0.015	0.169	0.122
Total effect	0.072	−0.016	0.169	0.124
Prop. mediated	0.000	−0.123	0.078	0.986

^a^ The model was adjusted for age and sex.

GLRX, glutaredoxin; TPA, total physical activity; CI, confidence interval; ACME, average causal mediation effect; ADE, average direct effect.

**Table 11.** Mediation analysis of SERPINE1 for cognitive function across LTPA levels

LTPA and SERPINE1	Estimate ^a^	Lower 95%CI	Upper 95% CI	*P* value
Reaction time test (*N* = 47,967)				
ACME	0.095	−0.001	0.203	0.054
ADE	−11.639	−13.745	−9.368	<0.001
Total effect	−11.544	−13.677	−9.301	<0.001
Prop. mediated	−0.008	−0.018	0.000	0.054
**Matching test (*N* = 48,429)**				
ACME	0.001	0.000	0.002	0.204
ADE	−0.080	−0.102	−0.057	<0.001
Total effect	−0.080	−0.102	−0.056	<0.001
Prop. mediated	−0.008	−0.024	0.005	0.204
**Fluid intelligence (*N* = 15,748)**				
ACME	−0.003	−0.007	0.001	0.118
ADE	0.307	0.238	0.381	<0.001
Total effect	0.305	0.236	0.379	<0.001
Prop. mediated	−0.008	−0.022	0.003	0.118
**Prospective memory (*N* = 16,310)**				
ACME	−0.001	−0.002	0.000	0.128
ADE	0.062	0.044	0.081	<0.001
Total effect	0.061	0.044	0.080	<0.001
Prop. mediated	−0.010	−0.028	0.004	0.128
**Numeric memory (*N* = 5,203)**				
ACME	0.003	−0.002	0.008	0.178
ADE	0.124	0.048	0.205	0.002
Total effect	0.127	0.051	0.208	<0.001
Prop. mediated	0.022	−0.012	0.089	0.178

^a^ The model was adjusted for age and sex.

SERPINE1, serpin family E member 1; LTPA, leisure-time physical activity; CI, confidence interval; ACME, average causal mediation effect; ADE, average direct effect.

**Table 12.** Mediation analysis of SERPINE1 for cognitive function across TPA levels

TPA and SERPINE1	Estimate ^a^	Lower 95%CI	Upper 95% CI	*P* value
Reaction time test (*N* = 52,011)				
ACME	0.098	0.010	0.200	0.030
ADE	−7.088	−9.529	−4.372	<0.001
Total effect	−6.990	−9.420	−4.276	<0.001
Prop. mediated	−0.014	−0.033	−0.002	0.030
**Matching test (*N* = 52,323)**				
ACME	0.001	0.000	0.002	0.102
ADE	−0.040	−0.066	−0.014	0.004
Total effect	−0.040	−0.065	−0.013	0.006
Prop. mediated	−0.021	−0.084	0.006	0.108
**Fluid intelligence (*N* = 16,918)**				
ACME	−0.002	−0.005	0.001	0.222
ADE	0.260	0.179	0.348	<0.001
Total effect	0.258	0.178	0.346	<0.001
Prop. mediated	−0.007	−0.023	0.005	0.222
**Prospective memory (*N* = 17,581)**				
ACME	−0.001	−0.002	0.000	0.098
ADE	0.036	0.016	0.058	<0.001
Total effect	0.035	0.015	0.058	<0.001
Prop. mediated	−0.017	−0.059	0.004	0.098
**Numeric memory (*N* = 5,525)**				
ACME	0.002	−0.001	0.006	0.188
ADE	0.070	−0.017	0.167	0.136
Total effect	0.072	−0.016	0.169	0.120
Prop. mediated	0.020	−0.092	0.282	0.284

^a^ The model was adjusted for age and sex.

SERPINE1, serpin family E member 1; TPA, total physical activity; CI, confidence interval; ACME, average causal mediation effect; ADE, average direct effect.

**Table 13.** Mediation analysis of GSN for cognitive function across LTPA levels

LTPA and GSN	Estimate ^a^	Lower 95%CI	Upper 95% CI	*P* value
Reaction time test (*N* = 47,967)				
ACME	0.023	−0.075	0.119	0.590
ADE	−11.570	−13.686	−9.280	<0.001
Total effect	−11.545	−13.648	−9.304	<0.001
Prop. mediated	−0.002	−0.010	0.007	0.590
**Matching test (*N* = 48,429)**				
ACME	0.000	−0.001	0.001	0.786
ADE	−0.080	−0.102	−0.056	<0.001
Total effect	−0.080	−0.102	−0.056	<0.001
Prop. mediated	0.001	−0.011	0.016	0.786
**Fluid intelligence (*N* = 15,748)**				
ACME	−0.004	−0.008	−0.001	0.016
ADE	0.308	0.239	0.382	<0.001
Total effect	0.304	0.235	0.379	<0.001
Prop. mediated	−0.013	−0.026	−0.002	0.016
**Prospective memory (*N* = 16,310)**				
ACME	0.000	−0.001	0.001	0.972
ADE	0.061	0.044	0.080	<0.001
Total effect	0.061	0.044	0.080	<0.001
Prop. mediated	0.000	−0.015	0.014	0.972
**Numeric memory (*N* = 5,203)**				
ACME	−0.001	−0.007	0.003	0.550
ADE	0.128	0.053	0.209	<0.001
Total effect	0.126	0.050	0.208	<0.001
Prop. mediated	−0.011	−0.077	0.032	0.550

^a^ The model was adjusted for age and sex.

GSN, gelsolin; LTPA, leisure-time physical activity; CI, confidence interval; ACME, average causal mediation effect; ADE, average direct effect.

**Table 14.** Mediation analysis of GSN for cognitive function across TPA levels

TPA and GSN	Estimate ^a^	Lower 95%CI	Upper 95% CI	*P* value
Reaction time test (*N* = 52,011)				
ACME	0.007	−0.097	0.108	0.868
ADE	−7.000	−9.420	−4.287	<0.001
Total effect	−6.993	−9.434	−4.300	<0.001
Prop. mediated	−0.001	−0.016	0.015	0.868
**Matching test (*N* = 52,323)**				
ACME	0.000	−0.001	0.001	0.810
ADE	−0.040	−0.065	−0.012	0.006
Total effect	−0.040	−0.065	−0.012	0.006
Prop. mediated	−0.003	−0.036	0.031	0.816
**Fluid intelligence (*N* = 16,918)**				
ACME	−0.003	−0.006	0.001	0.134
ADE	0.260	0.181	0.349	<0.001
Total effect	0.258	0.177	0.346	<0.001
Prop. mediated	−0.010	−0.027	0.003	0.134
**Prospective memory (*N* = 17,581)**				
ACME	0.000	−0.001	0.001	0.624
ADE	0.035	0.015	0.058	<0.001
Total effect	0.035	0.015	0.058	<0.001
Prop. mediated	0.005	−0.022	0.040	0.624
**Numeric memory (*N* = 5,525)**				
ACME	0.000	−0.008	0.007	0.950
ADE	0.072	−0.015	0.169	0.122
Total effect	0.072	−0.017	0.167	0.126
Prop. mediated	−0.001	−0.384	0.274	0.972

^a^ The model was adjusted for age and sex.

GSN, gelsolin; TPA, total physical activity; CI, confidence interval; ACME, average causal mediation effect; ADE, average direct effect.

### OGT-dependent O-GlcNAcylation promotes the protein stability of CTSB by blocking its ubiquitination-mediated degradation

**Fig. 4.**
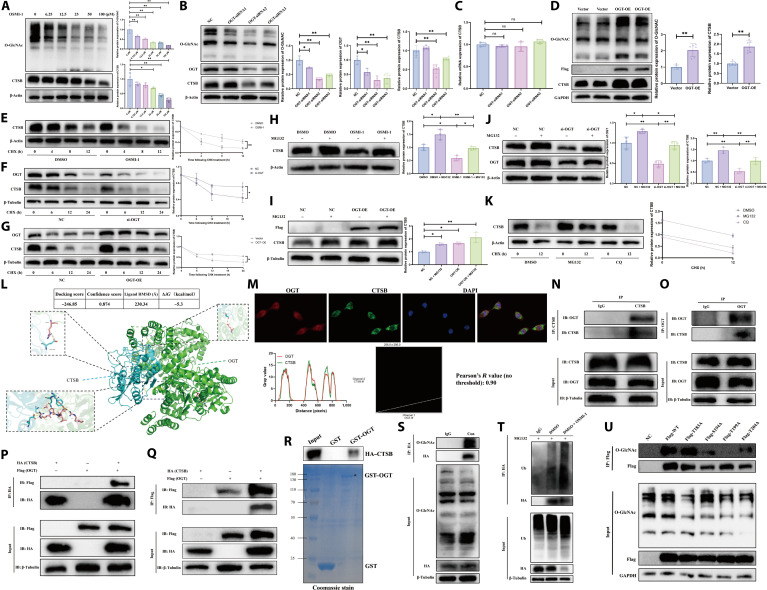
O-linked *N*-acetylglucosaminyltransferase (OGT)-dependent O-linked *N*-acetylglucosaminylation (O-GlcNAcylation) promotes cathepsin B (CTSB) protein stability by inhibiting its ubiquitination (Ub). (A) Western blotting (WB) analysis of changes in total O-GlcNAcylation and CTSB protein expression following treatment with different concentrations of OSMI-1. (B) WB analysis of changes in total O-GlcNAcylation, OGT, and CTSB protein expression after OGT knockdown. (C) Reverse transcription quantitative polymerase chain reaction (qPCR) analysis of changes in CTSB mRNA expression after OGT knockdown. (D) WB analysis of changes in total O-GlcNAcylation, Flag–OGT, and CTSB protein expression after OGT overexpression (OGT-OE). (E) WB analysis of changes in CTSB protein expression after treatment with OSMI-1 and cycloheximide (CHX). (F) WB analysis of changes in CTSB protein expression after treatment with small interfering RNA (siRNA) targeting OGT (si-OGT) and CHX. (G) WB analysis of changes in CTSB protein expression after treatment with OGT-OE and CHX. (H) WB analysis of changes in CTSB protein expression after treatment with OSMI-1 and MG132. (I) WB analysis of changes in CTSB protein expression after treatment with OGT-OE and MG132. (J) WB analysis of changes in CTSB protein expression after treatment with si-OGT and MG132. (K) WB analysis of changes in CTSB protein expression after treatment with MG132, chloroquine (CQ), and CHX. (L) Molecular docking analysis suggesting a potential physical interaction between CTSB and OGT proteins. (M) Immunofluorescence analysis of the colocalization of CTSB and OGT proteins. (N and O) Endogenous co-immunoprecipitation (IP) analysis suggesting a potential physical interaction between CTSB and OGT proteins. (P and Q) Exogenous co-IP analysis suggesting a potential physical interaction between CTSB and OGT proteins. (R) glutathione *S*-transferase (GST) pull-down assay suggesting a potential physical interaction between CTSB and OGT proteins. (S) IP analysis suggesting the presence of O-GlcNAcylation modification on CTSB protein. (T) IP analysis indicating that ubiquitination-mediated degradation of CTSB protein is regulated by OSMI-1. (U) Four point mutation plasmids were constructed to validate potential O-GlcNAcylation sites on the CTSB protein. *n* = 3; *P < 0.05; **P < 0.01. ns, not significant; GAPDH, glyceraldehyde-3-phosphate dehydrogenase; RMSD, root mean square deviation; IB, immunoblotting; HA, hemagglutinin; DMSO, dimethyl sulfoxide.

Although exercise-induced up-regulation of the CTSB expression has been reported, the underlying mechanisms remain incompletely understood. Signal pathway enrichment analysis from the aforementioned omics studies indicated that glucose metabolism and its associated glycosylation modifications may be closely related to exercise-promoted CTSB expression. Previous studies have reported that exercise can up-regulate the overall level of O-GlcNAcylation in muscle [23]. Given that O-GlcNAcylation is a broadly occurring PTM, we hypothesized that exercise may stabilize CTSB expression by enhancing its O-GlcNAcylation. O-GlcNAcylation site prediction website was used to untangle the presence of multiple potential O-GlcNAc modification sites in CTSB proteins across different species (Fig. S3A to C). A C2C12 myoblast differentiation model was successfully established (Fig. S3D). O-linked *N*-acetylglucosaminyltransferase (OGT) and O-GlcNAcase (OGA) are currently known as the pair of enzymes responsible for promoting and inhibiting O-GlcNAcylation, respectively. The OGT inhibitor OSMI-1 and the OGA inhibitor Thiamet G were used to up-regulate or down-regulate total O-GlcNAcylation levels in C2C12 cells. As demonstrated in Fig. 4A, OSMI-1 administration markedly reduced the protein expression of CTSB, while Thiamet G treatment exhibited no significant effect on CTSB protein levels (Fig. S3E), suggesting that CTSB protein is regulated by OGT-mediated O-GlcNAcylation. Consistently, OGT knockdown markedly inhibited CTSB expression (Fig. 4B), whereas OGT overexpression (OGT-OE) significantly promoted it (Fig. 4D). Importantly, OGT knockdown did not affect CTSB mRNA expression (Fig. 4C). Furthermore, cycloheximide (CHX) was used to inhibit protein synthesis (Fig. S3F). Under CHX treatment, both OSMI-1 (Fig. 4E) and small interfering RNA (siRNA) targeting OGT (si-OGT) (Fig. 4F) accelerated the degradation of CTSB protein, while OGT-OE delayed its degradation (Fig. 4G), indicating that OGT-dependent O-GlcNAcylation can enhance CTSB protein stability. In addition, MG132 was used to investigate whether CTSB degradation is influenced by OGT-dependent O-GlcNAcylation (Fig. S3G). In the presence of MG132, both OSMI-1 (Fig. 4H) and si-OGT (Fig. 4J) promoted CTSB degradation, whereas OGT-OE suppressed it (Fig. 4I), suggesting that OGT-dependent O-GlcNAcylation may inhibit the ubiquitination-mediated degradation of CTSB. Moreover, we further untangled that the degradation of CTSB protein primarily relies on the ubiquitin–proteasome pathway rather than the lysosomal pathway (Fig. 4K and Fig. S8A). Immunoprecipitation (IP) assay further demonstrated that OGT-dependent O-GlcNAcylation may promote O-GlcNAc modification of CTSB protein (Fig. 4S) and inhibit its ubiquitination-mediated degradation (Fig. 4T and Fig. S8B).

Moreover, the predicted results for CTSB protein O-GlcNAcylation sites across multiple species (*Homo sapiens*/*Mus musculus*/*Rattus norvegicus*) were intersected to identify 4 highly conserved potential CTSB O-GlcNAcylation sites (Fig. S3A to C). Four point mutation plasmids (T183A, S194A, T199A, and T204A) were constructed. IP experiments revealed that the T199 site on the CTSB protein is most likely to undergo O-GlcNAcylation (Fig. 4U and Fig. S8C). In addition, molecular docking (Fig. 4L), immunofluorescence (Fig. 4M), co-IP (Fig. 4N to Q), and glutathione *S*-transferase pull-down (Fig. 4R) assays consistently suggested a potential physical interaction between OGT and CTSB proteins, providing indirect evidence for the existence of O-GlcNAcylation on CTSB. Furthermore, overexpression of OGT and CTSB in mouse muscle tissue revealed a significant colocalization between OGT and CTSB expression via immunofluorescence staining (Fig. S4A and B). Subsequently, we further investigated whether the T199 site of CTSB is the critical O-GlcNAc modification site that promotes CTSB protein stability and inhibits its ubiquitination-mediated degradation. The results from MG132 treatment combined with CTSB T199 site mutation demonstrated that, compared to WT-CTSB-OE, mutation at the CTSB T199 site reduced CTSB protein expression (Fig. S6A and B). The CHX chase assay also confirmed that mutation at the CTSB T199 site accelerated CTSB protein degradation (Fig. S6C and E). Furthermore, OSMI-1 treatment accelerated CTSB protein degradation in the WT-CTSB-OE group but had no significant effect on CTSB protein expression in the CTSB T199 site mutation group (Fig. S6D and F). Finally, IP experimental results also showed that both OSMI-1 treatment and mutation at the CTSB T199 site enhanced the ubiquitination of CTSB protein in the WT-CTSB-OE group, whereas OSMI-1 treatment did not significantly affect the ubiquitination level of CTSB protein in the CTSB T199 site mutation group (Fig. S6G and H). These results indicate that OGT may promote CTSB O-GlcNAcylation and enhance its protein stability by suppressing ubiquitin-dependent degradation.

### Treadmill running promotes hippocampal neurogenesis and cognitive function in WT mice via activation of the muscle OGT/CTSB axis

**Fig. 5.**
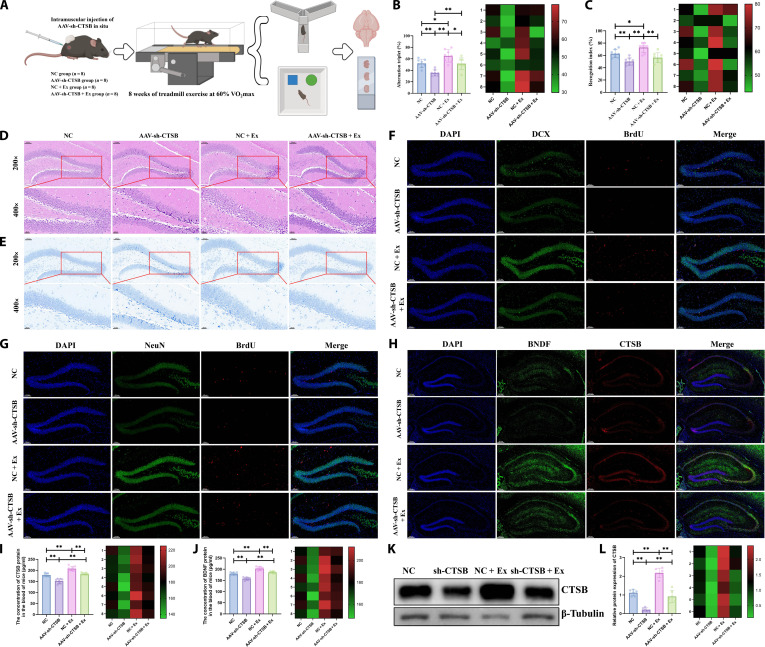
Knockdown of cathepsin B (CTSB) in skeletal muscle partially reverses the exercise-induced promotion of hippocampal neurogenesis and cognitive function in wild-type (WT) mice. (A) Schematic diagram of the animal experimental design. (B) Y-maze test in WT mice. (C) Novel object recognition test in WT mice. (D) Hematoxylin–eosin (HE) staining of brain tissue in WT mice. (E) Nissl staining of brain tissue in WT mice. (F and G) Immunofluorescence detection of hippocampal neurogenesis in WT mice. (H) Immunofluorescence detection of CTSB and brain-derived neurotrophic factor (BDNF) protein expression in the hippocampus of WT mice. (I) Enzyme-linked immunosorbent assay (ELISA) detection of CTSB protein expression in the serum of WT mice. (J) ELISA detection of BDNF protein expression in the serum of WT mice. (K and L) Western blotting (WB) analysis of CTSB protein expression in skeletal muscle tissue of WT mice. *n* = 8; *P < 0.05; **P < 0.01.

To further validate that exercise promotes hippocampal neurogenesis by up-regulating the CTSB expression in mouse muscle tissue, we locally injected adeno-associated virus (AAV)-mediated short hairpin RNA targeting CTSB (AAV-sh-CTSB) into the mouse muscle to knock down CTSB protein expression (Fig. 5A). As illustrated in Fig. 5B, results from the Y-maze test indicated that knocking down muscle CTSB partially reversed the promotive effect of treadmill running on the correct alternation rate. The novel object recognition test further confirmed that treadmill running effectively enhanced learning and memory performance, whereas inhibition of muscle CTSB significantly reduced the novel object recognition index (Fig. 5C). Furthermore, HE staining (Fig. 5D) and Nissl staining (Fig. 5E) results demonstrated that knocking down muscle CTSB significantly reduced the number of hippocampal neurons, although no obvious neuronal loss or necrosis was observed. Treadmill running markedly improved the volume and morphology of hippocampal Nissl bodies in CTSB-knockdown mice and increased neuronal count and density, suggesting that treadmill running may improve hippocampal neurohistological morphology by promoting muscle CTSB expression. Further, treadmill running significantly boosted the number of BrdU^+^/DCX^+^ and BrdU^+^/NeuN^+^ cells, while AAV-sh-CTSB partially reversed the facilitative effects of treadmill running on hippocampal neurogenesis (Fig. 5F and G and Fig. S9D to F). We also examined the expression levels of BDNF and CTSB proteins in mouse hippocampal tissue via immunofluorescence staining (Fig. 5H and Fig. S9M and N). The results showed that after 8 weeks of treadmill running, the protein expression of both BDNF and CTSB were significantly increased in the hippocampus. However, knocking down muscle CTSB using AAV-sh-CTSB impaired the exercise-induced up-regulation of BDNF and CTSB expression in hippocampal tissue. The expression trends of CTSB and BDNF in serum were consistent with those in hippocampal tissue (Fig. 5I and J). In addition, Western blotting (WB) was performed to assess CTSB protein levels in muscle tissue, confirming that AAV-sh-CTSB reversed the exercise-induced up-regulation of muscle CTSB (Fig. 5K and L). These results preliminarily untangle that treadmill running may improve hippocampal neurogenesis and memory function in WT mice by up-regulating CTSB expression in muscle tissue.

**Fig. 6.**
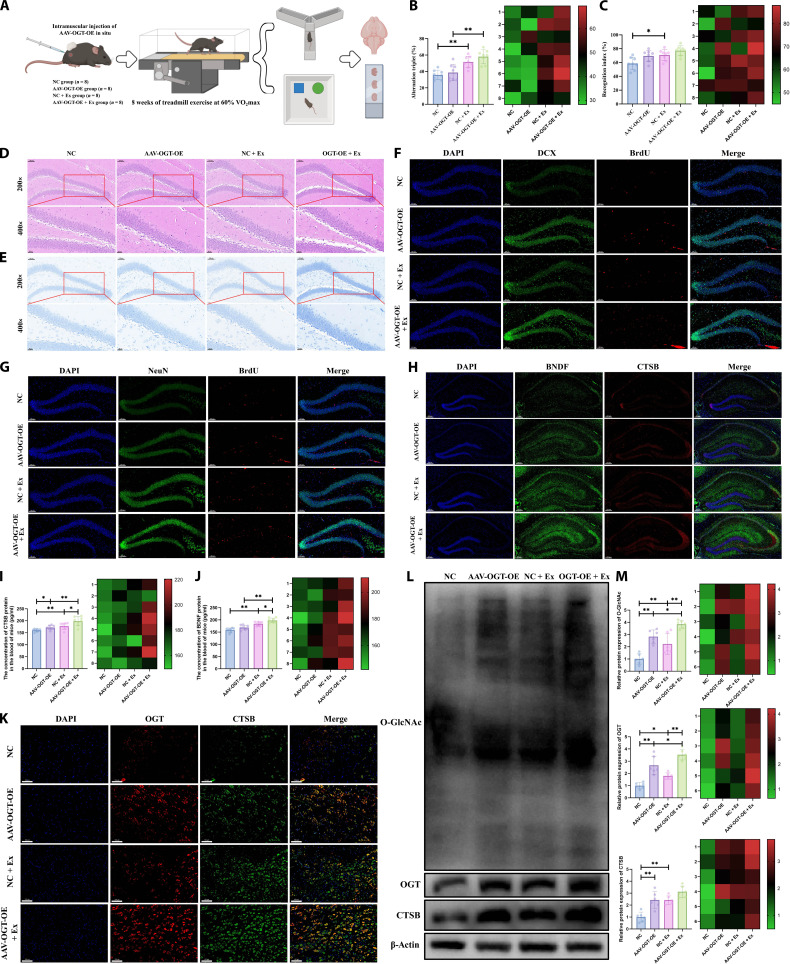
Overexpression of O-linked *N*-acetylglucosaminyltransferase (OGT) in skeletal muscle enhances the beneficial effects of treadmill running on hippocampal neurogenesis and cognitive function in wild-type (WT) mice. (A) Schematic diagram of the animal experimental design. (B) Y-maze test in WT mice. (C) Novel object recognition test in WT mice. (D) Hematoxylin–eosin (HE) staining of brain tissue in WT mice. (E) Nissl staining of brain tissue in WT mice. (F and G) Immunofluorescence detection of hippocampal neurogenesis in WT mice. (H) Immunofluorescence detection of cathepsin B (CTSB) and brain-derived neurotrophic factor (BDNF) protein expression in the hippocampus of WT mice. (I) Enzyme-linked immunosorbent assay (ELISA) detection of CTSB protein expression in the serum of WT mice. (J) ELISA detection of BDNF protein expression in the serum of WT mice. (K) Immunofluorescence detection of CTSB and BDNF protein expression in skeletal muscle tissue of WT mice. (L and M) Western blotting (WB) analysis of total O-linked *N*-acetylglucosaminylation (O-GlcNAcylation), OGT, and CTSB protein expression in skeletal muscle tissue of WT mice. *n* = 8; *P < 0.05; **P < 0.01.

Subsequently, AAV-OGT-OE was injected in situ into mouse skeletal muscle to elevate OGT protein expression, aiming to investigate whether treadmill running promotes hippocampal neurogenesis in WT mice via the OGT/CTSB axis (Fig. 6A). As demonstrated in Fig. 6B, the Y-maze test results indicated that, consistent with prior findings, treadmill running significantly improved the correct alternation rate in the Y-maze, reflecting enhanced short-term spatial working memory. Although mice with muscle-specific OGT-OE also exhibited an increasing trend in correct alternation rate, the difference was not statistically significant. The novel object recognition test revealed that the recognition index was markedly higher in the AAV-OGT-OE + exercise (Ex) group compared to the NC (negative control; for AAV-OGT-OE) + Ex group (Fig. 6C). Both HE staining (Fig. 6D) and Nissl staining (Fig. 6E) results demonstrated that treadmill running effectively boosted both the quantity and density of hippocampal neurons. Similarly, elevated OGT levels in muscle tissue exerted a positive effect on hippocampal neuronal morphology. Furthermore, double immunofluorescence staining for BrdU/DCX (Fig. 6F and Fig. S9G) and BrdU/NeuN (Fig. 6G and Fig. S9H and I) revealed that AAV-OGT-OE significantly raised the count of BrdU^+^/DCX^+^ and BrdU^+^/NeuN^+^ double-labeled cells in the hippocampus, mirroring the neurogenesis-promoting effects of exercise. The combination of AAV-OGT-OE and treadmill running resulted in an even more pronounced increase. In addition, immunofluorescence staining indicated that AAV-OGT-OE and treadmill running acted synergistically to increase the levels of BDNF and CTSB proteins in hippocampal tissue (Fig. 6H and Fig. S9O and P). Serum concentrations of BDNF and CTSB showed consistent alterations (Fig. 6I and J). Moreover, protein expression of OGT and CTSB in muscle tissue was assessed via immunofluorescence and WB. We found that treadmill running promoted the expression of both OGT and CTSB in muscle tissue (Fig. 6K to M). Importantly, OGT-OE further enhanced CTSB protein expression in mouse muscle. These findings suggest that treadmill running may promote hippocampal neurogenesis and improve memory function in WT mice potentially through activating the muscle OGT/CTSB axis.

### Treadmill running improves hippocampal neurogenesis and cognitive function in APP/PS1 mice by promoting the release of the myokine CTSB

**Fig. 7.**
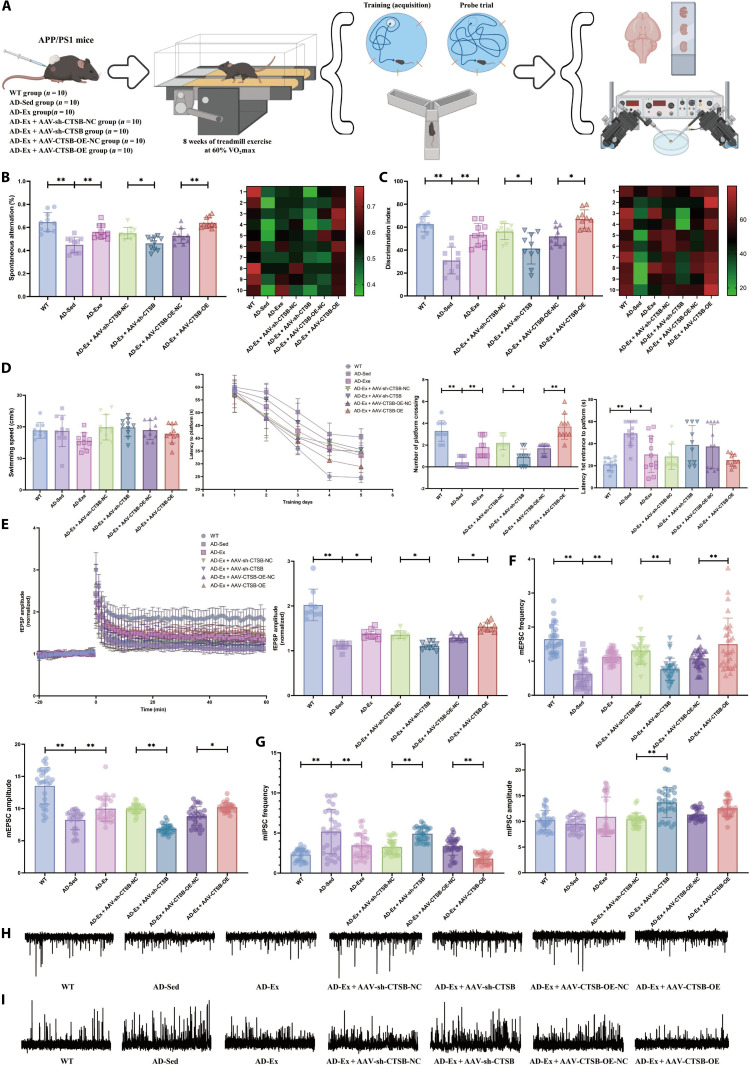
Effects of knockdown or overexpression of cathepsin B (CTSB) expression in muscle tissue on memory and cognitive function in amyloid precursor protein/presenilin 1 (APP/PS1) mice. (A) Schematic diagram of the animal experimental design. (B) Y-maze test in APP/PS1 mice. (C) Novel object recognition test in APP/PS1 mice. (D) Morris water maze test in APP/PS1 mice. (E to I) Patch–clamp recordings in brain tissues of APP/PS1 mice. *n* = 10; *P < 0.05; **P < 0.01.

**Fig. 8.**
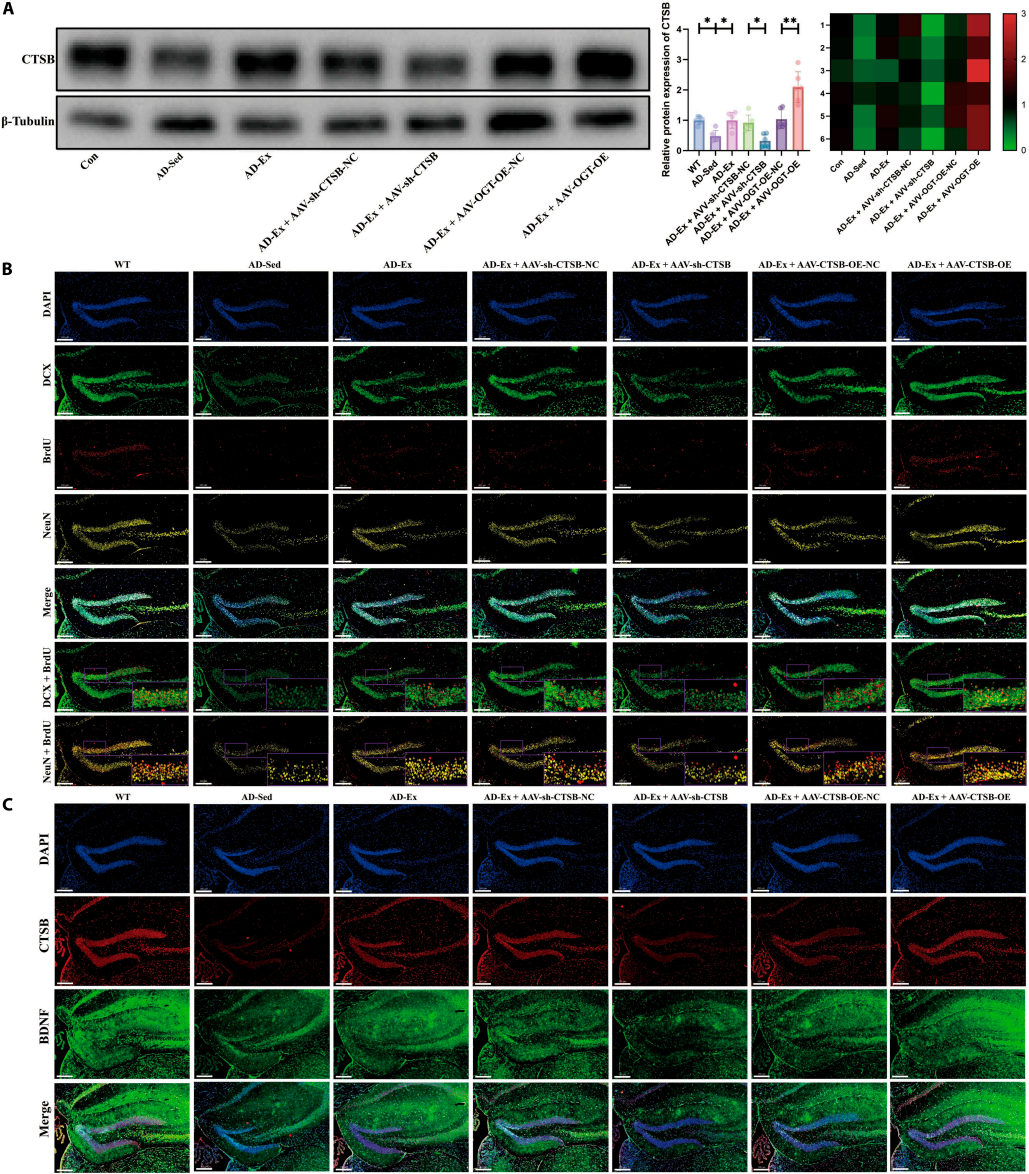
Effects of knockdown or overexpression of cathepsin B (CTSB) in muscle tissue on hippocampal neurogenesis and the protein expression of CTSB and brain-derived neurotrophic factor (BDNF) in amyloid precursor protein/presenilin 1 (APP/PS1) mice. (A) Protein expression of CTSB in muscle tissue of APP/PS1 mice. (B) Detection of hippocampal neuronal proliferation in APP/PS1 mice detected by immunofluorescence. (C) Protein expression of CTSB and BDNF in hippocampal tissue of APP/PS1 mice detected by immunofluorescence. *n* = 6; *P < 0.05; **P < 0.01.

To further investigate whether treadmill running improves hippocampal neurogenesis and cognitive function in APP/PS1 mice by up-regulating CTSB expression in muscle tissue, we used AAV-sh-CTSB and AAV-CTSB-OE to knock down or overexpress CTSB, respectively, in the muscle of APP/PS1 mice, combined with treadmill running intervention for validation (Fig. 7A). Results from the Y-maze (Fig. 7B), novel object recognition (Fig. 7C), and Morris water maze (Fig. 7D) tests showed that 8 weeks of treadmill running significantly enhanced cognitive function in APP/PS1 mice. Knockdown of muscle CTSB partially reversed the cognitive improvement induced by treadmill running, while CTSB overexpression further enhanced the beneficial effects of treadmill running on cognition. Further, as shown in Fig. 6E to I, patch–clamp techniques were used to evaluate hippocampal synaptic plasticity and synaptic transmission in APP/PS1 mice by measuring long-term potentiation (LTP) and miniature excitatory/inhibitory postsynaptic currents (mEPSCs/mIPSCs). LTP results (Fig. 7E) revealed that, compared to the WT group, the field excitatory postsynaptic potential (fEPSP) amplitude was significantly reduced in the AD-sedentary (Sed) group, indicating impaired synaptic plasticity. Treadmill running significantly increased fEPSP amplitude and partially restored synaptic function. Knockdown of muscle CTSB inhibited the exercise-induced improvement in LTP, while CTSB overexpression further enhanced the effect of exercise, suggesting that treadmill running may improve synaptic plasticity in APP/PS1 mice by up-regulating CTSB expression in muscle tissue. mEPSC recordings (Fig. 7F) showed that both the frequency and amplitude of mEPSCs were significantly diminished in APP/PS1 mice than those observed in WT mice. Treadmill running substantially elevated mEPSC frequency, an effect that was attenuated by CTSB knockdown and further enhanced by CTSB overexpression, indicating improved excitatory synaptic transmission. Similarly, mIPSC results (Fig. 7G) demonstrated a notable decrease in mIPSC frequency in APP/PS1 mice, which was partially restored by treadmill running. Knockdown of CTSB again reduced frequency, while CTSB overexpression significantly increased it, suggesting that CTSB is also involved in regulating inhibitory synaptic function. The trends in spontaneous neuronal activity across groups, as illustrated by representative mEPSC and mIPSC traces (Fig. 7H and I), were consistent with the above results. These findings further support that treadmill running, by up-regulating CTSB expression in muscle tissue, not only promotes neurogenesis but also markedly enhances synaptic plasticity and synaptic transmission efficiency in APP/PS1 mice, providing electrophysiological evidence for the improvement of cognitive function. Results of HE staining (Fig. S5A) and Nissl staining (Fig. S5B) demonstrated that the quantity and arrangement of hippocampal neurons were markedly increased in the AD-Ex group relative to the AD-Sed group. Neuronal structure was impaired upon muscle-specific CTSB knockdown, while CTSB overexpression further enhanced neuronal structural integrity, as evidenced by more tightly packed hippocampal neurons. As shown in Fig. S5C, thioflavin S staining indicated significantly enhanced amyloid-β (Aβ) fluorescence in the brain tissue of AD-Sed mice relative to the WT controls, while treadmill running intervention markedly reduced cerebral Aβ accumulation. Muscle-specific CTSB knockdown attenuated the inhibitory effect of treadmill running on Aβ accumulation, whereas CTSB overexpression further enhanced the benefits of treadmill running. Glycine silver immersion plating nerve staining was used to assess the extent of neurofibrillary degeneration in the brain tissues across groups (Fig. S5D). The results demonstrated an increase in silver-positive deposits in the AD-Sed group. Muscle-specific CTSB knockdown promoted neurofilament fragmentation and deposit formation, while the CTSB overexpression group exhibited partial restoration of neurofilament structural integrity and a significant reduction in silver-positive staining. Furthermore, immunohistochemistry was performed to assess the presence of astrocytes (glial fibrillary acidic protein [GFAP]) and microglia (ionized calcium-binding adapter molecule 1 [Iba1]) (Fig. S5E to H). The results showed significantly elevated expression of GFAP and Iba1 in brain tissues of the AD-Sed group, indicating marked neuroinflammatory responses. In contrast, the AD-Ex group exhibited reduced neuroinflammation. Knockdown of muscle CTSB promoted the expression of GFAP and Iba1, while CTSB overexpression further suppressed their expression. Furthermore, consistent with our previous findings, enzyme-linked immunosorbent assay (ELISA) analysis also revealed that treadmill running significantly promotes the circulating levels of CTSB and BDNF proteins in APP/PS1 mice (Fig. S5I). In addition, WB was performed to examine CTSB protein expression in muscle tissues of WT and variously treated APP/PS1 mice (Fig. 8A). Consistent with previous findings, treadmill running promoted CTSB expression in muscle tissue. Both muscle-specific CTSB knockdown and overexpression resulted in significant changes. As shown in Fig. 8B and Fig. S9J to L, immunofluorescence results indicated impaired hippocampal neurogenesis in the AD-Sed group relative to the WT controls, which was partially reversed by 8 weeks of treadmill running. Knockdown of CTSB attenuated the exercise-induced promotion of hippocampal neurogenesis, while CTSB overexpression further enhanced the proneurogenic effects of exercise. Importantly, consistent with earlier observations in WT mice, the data revealed a pronounced elevation of CTSB and BDNF proteins in the brain of AD mice following treadmill running. Muscle-specific CTSB knockdown or overexpression respectively impaired or enhanced the expression of cerebral CTSB and BDNF (Fig. 8C and Fig. S9Q and R). These results imply that myogenic CTSB mediates the beneficial effects of treadmill running on hippocampal neurogenesis and cognitive performance in APP/PS1 mice.

### Myokine CTSB may be delivered via EVs to mediate treadmill-running-induced improvement of cognitive function

**Fig. 9.**
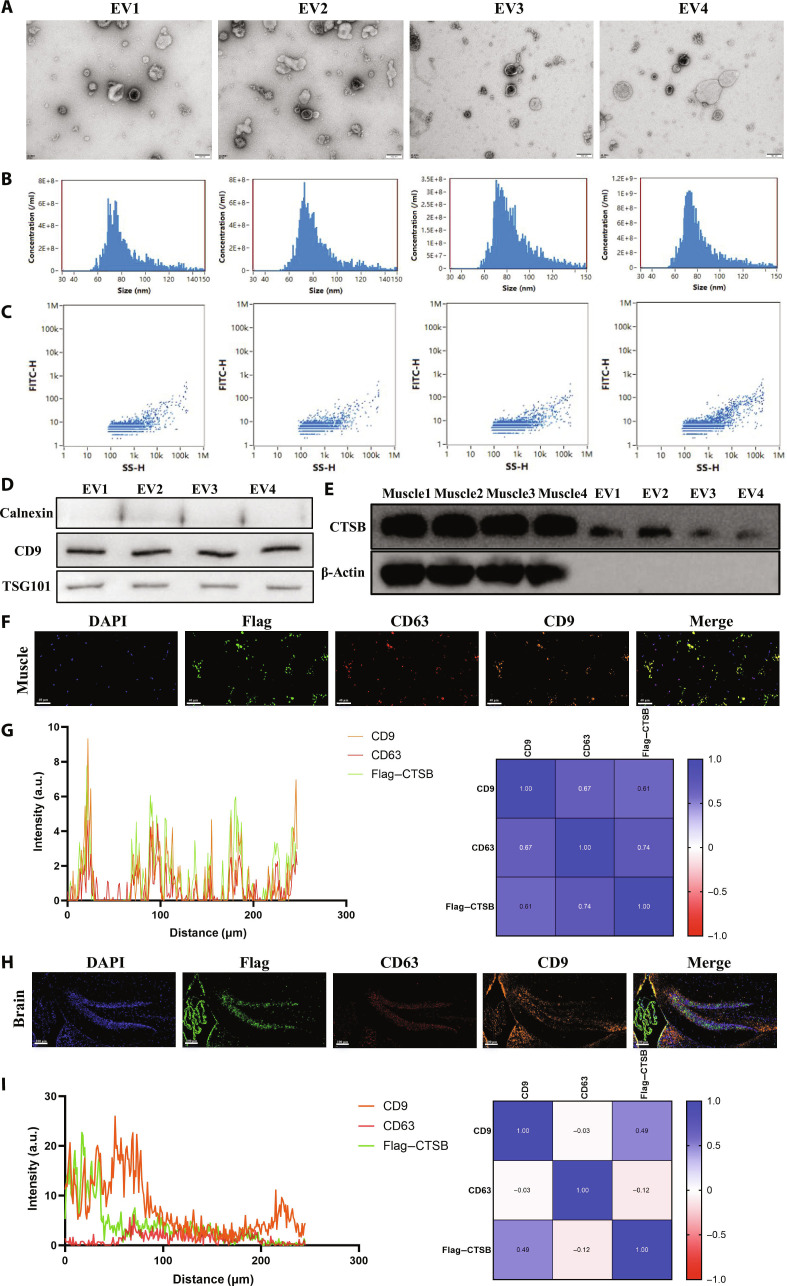
The myokine cathepsin B (CTSB) may be released via extracellular vesicles (EVs). (A to D) Identification of EVs derived from muscle tissue of amyloid precursor protein/presenilin 1 (APP/PS1) mice via transmission electron microscopy, particle size analysis, and protein marker detection. FITC-H, fluorescein isothiocyanate-height; SS-H, side scatter-height. (E) Western blotting (WB) analysis of CTSB protein expression in muscle tissue and muscle-derived EVs of APP/PS1 mice. (F and G) Immunofluorescence colocalization analysis of Flag–CTSB and EV protein markers in muscle tissue of AAV-CTSB-OE mice. (H and I) Immunofluorescence colocalization analysis of Flag–CTSB and EV protein markers in hippocampal tissue of AAV-CTSB-OE mice.

Although our preliminary findings suggest that treadmill running may improve hippocampal neurogenesis and cognitive function by promoting the release of the myokine CTSB from mouse muscle tissue, it remains unclear whether CTSB is secreted into the bloodstream via EVs and subsequently reaches the brain via systemic circulation. Previous research indicates that physical exercise can enhance cognitive performance by promoting the release of microRNAs from rat muscle into the bloodstream via EVs [30], indicating that myogenic CTSB may also be secreted into the blood via EVs, which can protect their cargo from degradation during circulation. To evaluate the hypothesis, we isolated EVs from the muscle tissue of mice (Fig. 9A to D). WB analysis confirmed the presence of CTSB in muscle-derived EVs (Fig. 9E), indicating that the myokine CTSB can be secreted from muscle tissue via EVs. Furthermore, in muscle tissue of mice overexpressing Flag-tagged CTSB, Flag expression showed a strong correlation with the EV markers CD63 and CD9 (Fig. 9F and G). Importantly, the presence of Flag protein was confirmed in the brains of mice overexpressing Flag-tagged CTSB (via intramuscular injection), providing direct evidence that myogenic CTSB can reach the brain (Fig. 9H). Although there was no significant link observed between Flag protein and the EV markers CD63 and CD9 in brain tissue (Fig. 9I), this may be attributed to the diverse cellular origins of brain EVs, which are not exclusively derived from muscle tissue. These results preliminarily indicate that myogenic CTSB may be secreted into the bloodstream via EVs to facilitate intertissue communication.

In addition, at the cellular level, it was further demonstrated that exercise might promote the expression of the CTSB protein in C2C12 cells and alleviate Aβ-induced damage in HT22 cells via releasing EVs. First, C2C12 cells were treated with 100 μM 5-aminoimidazole-4-carboxamide 1-β-D-ribofuranoside (AICAR) (an adenosine monophosphate kinase agonist) to simulate the effects of exercise on myoblasts (Fig. S7A). AICAR supplementation was found to promote total O-GlcNAcylation levels and CTSB protein expression in C2C12 cell line (Fig. S7B). Furthermore, we isolated EVs from the C2C12 cell line and examined the effects of myoblast-derived EVs on Aβ-induced HT22 cells. EV protein marker detection (Fig. S7C), particle size analysis (Fig. S7D), and transmission electron microscopy (Fig. S7E) confirmed the successful isolation of myoblast-derived EVs. Similar to the EV contents extracted from mouse muscle tissue, we also detected CTSB protein expression in EVs derived from C2C12 cells via WB (Fig. S7C). EV uptake assays confirmed that 1,1′-dioctadecyl-3,3,3′,3′-tetramethylindocarbocyanine perchlorate-labeled myoblast-derived EVs were internalized by HT22 cells (Fig. S7F). Moreover, reverse transcription quantitative polymerase chain reaction (qPCR) results demonstrated that different concentrations of EVs derived from myoblasts treated with AICAR (100 μM for 24 h) significantly promoted HT22 cell proliferation and highlighted the expression of plasticity-related genes (Fig. S7G). Reactive oxygen species detection also revealed that AICAR-EVs at various concentrations partially reversed Aβ-induced reactive oxygen species accumulation in HT22 cells (Fig. S7H). In addition, flow cytometry analysis indicated that AICAR-EVs inhibited Aβ-induced apoptosis in HT22 cells (Fig. S7I). This finding offers profound insights into the mechanisms underlying muscle–brain cross-talk.

## Discussion

**Fig. 10.**
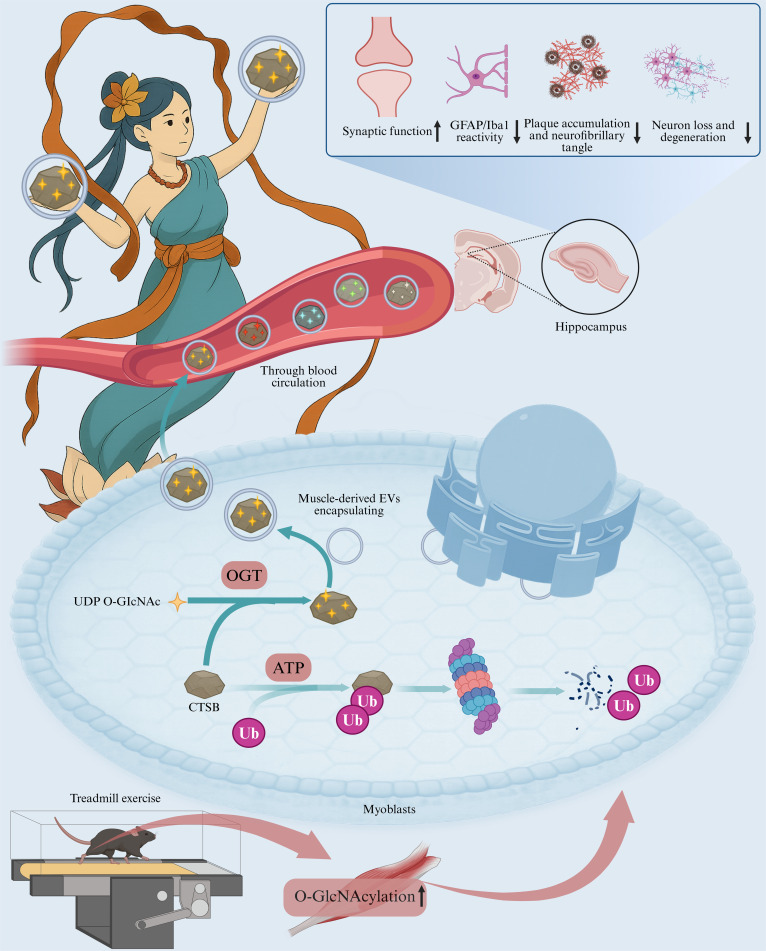
Molecular mechanism diagram of this study (created in https://BioRender.com). UDP, uridine diphosphate; ATP, adenosine triphosphate.

Our results, as depicted in Fig. 10, indicate that muscle-derived CTSB is stabilized through O-GlcNAcylation mediated by OGT and may be secreted into the bloodstream via EVs, thereby enhancing hippocampal neuronal differentiation, synaptic plasticity, as well as memory and cognitive function in mice. These results establish the crucial role of the OGT/CTSB axis in exercise-induced cognitive improvement and emphasize the scientific value and reproducibility of precisely controlling exercise dosage based on VO_2_max. The exploration of this dose–response relationship provides new insights that may guide future exercise strategies targeting AD and related neurodegenerative disorders.

Some studies have indicated that excessively high exercise intensity may impair cognition [31], although others have reported that high-intensity exercise can reduce amyloid deposition and improve cognitive function [32]. We propose that these contradictory findings are closely related to the selection of exercise protocols and the definition of exercise intensity. Based on measurements of VO_2_max, our study designed exercise regimens of varying intensities and durations and demonstrated that dose-dependent treadmill running enhances hippocampal neurogenesis and cognitive performance. However, no significant cognitive impairment was observed in the 80% VO_2_max group, which may also be attributed to the exercise frequency (every other day) not reaching a harmful excessive dosage. VO_2_max, a critical indicator of the body’s capacity to capture and exploit oxygen, can be quantified through incremental load tests. Previous studies have confirmed that exercise intensity defined as a proportion of VO_2_max enables the design of objective exercise protocols [33–35]. In our study, exercise intensity was stratified into 20% VO_2_max, 40% VO_2_max, 60% VO_2_max, and 80% VO_2_max based on VO_2_max test in mice. Mice in the treadmill running groups underwent training 3 times per week according to their respective running protocols, with each session lasting 1 h, for a duration of 2 to 8 weeks. The step-down avoidance test and the 8-arm maze test demonstrated that learning and memory performance improved progressively with increasing exercise intensity and duration. Histopathological analyses confirmed that treadmill running serves as a potent modulator of hippocampal neurogenesis and memory enhancement. Earlier studies have demonstrated that engaging in moderate-intensity aerobic exercise enhances pattern separation and LTP within the dentate gyrus of the hippocampus [36,37]. Evidence suggests that moderate aerobic exercise may boost anterior hippocampal volume by approximately 2% over 12 months and improve spatial memory, with volume changes correlating with serum BDNF levels [33]. Our animal experimental data not only replicate this phenomenon but also offer a more nuanced analysis of the effects of exercise at varying intensities and durations. The results align with previous studies indicating that moderate exercise yields the greatest cognitive benefits. Previous studies have indicated that moderate training can maximize BDNF secretion. Hippocampal BDNF protein expression increases with the volume of exercise; however, beyond a certain threshold, excessive exercise induces oxidative stress that impairs neural signaling and compromises cognitive function [38]. In other words, both excessively low and high intensities of exercise yield suboptimal benefits, with moderate intensity being most favorable. Although our highest intensity group (80% VO_2_max) did not exhibit cognitive decline and still showed increased BDNF levels, its cost-effectiveness is limited because of reduced exercise compliance and an elevated risk of cartilage damage. Our study demonstrates that training at 60% VO_2_max every other day for 8 weeks substantially enhances cognitive function in mice, which aligns with previous findings suggesting that moderate-intensity exercise is superior to a sedentary lifestyle and outperforms both insufficient and excessive intensity regimens [39]. Therefore, the optimal intensity threshold for cognitive benefits may be highly dependent on the specific exercise training regimen, highlighting the need for future research to focus more on the scientific design of exercise protocols.

A second major finding of this present study is the emphasis on myokine CTSB mediates the cognitive effects of treadmill running, along with the preliminary untangle that CTSB is released via EVs derived from muscle tissue. Through integrated omics analysis and data from the UK Biobank cohort, CTSB was identified as a key exercise-responsive myokine that may mediate the cognitive-enhancing effects of PA. Specifically, analysis of the UK Biobank cohort data demonstrated that CTSB exhibits significant mediating effects in the relationship between PA (LTPA and TPA) and various types of cognitive impairments—including reaction time, matching test, fluid intelligence, prospective memory, and numeric memory—with stronger mediating effects observed at moderate exercise intensities. Furthermore, we observed that CTSB protein levels in mouse muscle tissue increased with both the intensity and duration of treadmill running. WB and ELISA results confirmed concomitant elevations of CTSB expression in both plasma and hippocampal tissues. These findings align with previous research in which proteomic analysis of runners’ serum identified CTSB as a secreted factor up-regulated after long-term running, and exogenous CTSB was shown to enhance BDNF and DCX expression in neural progenitor cells [18]. Other human and primate studies have also reported elevated plasma CTSB levels following endurance exercise, with individuals exhibiting higher fitness levels demonstrating greater CTSB levels and better cognitive performance [40–42]. Importantly, our mechanistic studies extend these observations: We verified via WB that muscle-derived EVs are enriched with CTSB. Moreover, immunofluorescence staining revealed that exogenously expressed Flag-tagged CTSB, injected in situ into muscle tissue, could be detected in brain tissue. These data provide direct evidence that CTSB may be transported from muscle to the brain via EVs, supporting the concept of a muscle–brain axis regulatory network. Accumulating evidence indicates that EVs, serving as key mediators of paracrine signaling, protect their cargo from degradation and facilitate intertissue/organ communication by transporting proteins, nucleic acids, and lipids through the circulation to distant target organs [43,44]. Recent investigations indicate that exercise triggers the rapid release of muscle-derived EVs carrying microRNAs, thereby enhancing synaptic plasticity and memory [45]. In a chronic cerebral hypoperfusion mouse model, administration of muscle-derived EVs enriched with miR-17/20a-5p activated the mammalian target of rapamycin pathway and restored cognitive function, demonstrating that systemic delivery of these EVs enhances synaptic plasticity and memory, thereby ameliorating vascular dementia [30]. Other myokines, such as interleukin-6 and insulin-like growth factor 1, have also been reported to cross the BBB and influence neurogenesis, although their transport has not been proven to depend on EVs [46–48]. Our preliminary findings suggest that the myokine CTSB may be packaged and secreted via muscle-derived EVs and transported to the brain, where it improves memory and cognitive function. This indicates that EV-mediated transport could be a crucial pathway for CTSB to reach the brain and exert its effects, providing a mechanistic framework for muscle–brain communication. This pathway also offers an explanation for the exercise-induced increase in circulating CTSB. Notably, exercise-enhanced O-GlcNAcylation promotes the stability of CTSB protein in muscle, enabling its efficient packaging and transport to the brain. In summary, our study highlights muscle-derived EVs as potential key carriers in exercise-mediated muscle–brain communication. However, further experimental evidence is needed to validate and explore the EVs release and transport of the myokine CTSB, as well as its downstream regulatory networks in the brain.

The third key finding of this study is that exercise can promote the stable expression of CTSB by facilitating its O-GlcNAcylation. O-GlcNAcylation is a widespread and dynamic PTM regulated by the transferase OGT and the hydrolase OGA [49,50]. Previous studies indicate that increasing OGT levels in hippocampal neurons improves associative and spatial memory and reverses aging-related cognitive decline [51–53]. O-GlcNAcylation is associated with synaptic plasticity and cognitive function; increasing O-GlcNAc levels through OGT-OE or OGA inhibition improves memory function in aged mice [54,55]. Our study emphasizes the role of OGT-mediated O-GlcNAcylation in the muscle–brain axis and proposes that O-GlcNAcylation of CTSB in muscle tissue may enhance its accumulation and secretion, thereby improving brain function. In present study, treadmill running was found to up-regulate OGT and total O-GlcNAcylated protein levels in muscle tissue. Immunofluorescence staining revealed colocalization of OGT and CTSB. Knockdown of OGT or inhibition of O-GlcNAcylation with OSMI-1 reduced CTSB protein expression, whereas OGT-OE enhanced CTSB stability and suppressed its ubiquitin-mediated degradation. A previous oncology study reported that glucose metabolism promotes O-GlcNAcylation at the S210 site of CTSB in macrophages [56,57]. In our study, IP combined with site-directed mutagenesis indicated that O-GlcNAcylation occurs at the T199 site of CTSB. These results imply that although O-GlcNAcylation of CTSB may be a common regulatory mechanism across tissues, the functional O-GlcNAcylation sites may vary. Previous research has identified CTSB as a crucial intermediary by which exercise improves cognition in WT mice [18], which is consistent with our results. We found that intramuscular injection of AAV-sh-CTSB partially reversed the exercise-induced promotion of hippocampal neurogenesis and memory in WT mice. Furthermore, intramuscular injection of AAV-OGT-OE increased CTSB expression in muscle, further improving memory and cognitive performance and enhancing neurogenesis in WT mice. Importantly, we extended this mechanistic insight to an AD model. We further validated the beneficial effects of muscle-derived CTSB on hippocampal neurogenesis and cognition in APP/PS1 mice. Eight weeks of treadmill running markedly improved spatial memory in APP/PS1 mice. Electrophysiological recordings from hippocampal slices showed that treadmill running restored LTP amplitude and increased the frequency of both mEPSCs and mIPSCs, indicating improved synaptic transmission. Notably, knockdown of muscle CTSB attenuated the benefits of exercise, while CTSB overexpression further enhanced these effects. These results align with recent studies demonstrating that long-term voluntary running in APP/PS1 mice partially up-regulates hippocampal BDNF, promotes neurogenesis, ameliorates amyloid pathology, improves cognition, and enhances microglial glucose metabolism and morphological plasticity [58,59].

In addition, several studies have indicated that the absence of CTSB ameliorates neuroinflammation and cognitive function in disease models such as chronic periodontitis or traumatic brain injury, suggesting that the role of CTSB may be pathology specific [60,61]. However, most of these studies used systemic knockout or pharmacological inhibition, which may affect multiple tissues and organs. Our data demonstrate that modulating muscular CTSB under healthy exercise conditions plays a critical role, underscoring that tissue specificity is an important determinant of functional outcomes. Our findings also highlight that treadmill running increases the expression levels of OGT and O-GlcNAcylation in muscle tissue, thereby conferring cognitive benefits, suggesting that OGT in muscle may serve as a potential target for exercise-induced benefits. In summary, this study systematically untangles the key mechanisms through which treadmill running improves cognition via the muscle–brain axis. We established a dose–response relationship between exercise-intensity-formulated based on VO_2_max and both hippocampal neurogenesis and cognitive function. Notably, in our study, the primary evaluation metrics for hippocampal neurogenesis included BrdU^+^/DCX^+^, BrdU^+^/NeuN^+^, and BrdU^−^/NeuN^+^ cells. BrdU^+^/DCX^+^ cells represent newly generated and immature neurons, indicating that neurogenesis is in progress and the cells are at an early stage of differentiation. BrdU^+^/NeuN^+^ cells represent newly generated neurons that have already expressed a mature neuronal marker, indicating that neurogenesis has successfully produced new, mature neurons. BrdU^−^/NeuN^+^ cells represent preexisting mature neurons present prior to the experiment. An increase in the number of BrdU^−^/NeuN^+^ cells demonstrates a neuroprotective effect, whereas a decrease suggests ongoing neuronal death. Although the 5 consecutive days of BrdU injection prior to mouse euthanasia were insufficient for labeling the maturation process of newborn neurons (typically 28 d), it has been documented that some newly generated neurons (certain interneuron subtypes) can initiate NeuN expression shortly after exit from the cell cycle. As noted by Lai et al. [62], these cells are characterized by fainter NeuN immunoreactivity and smaller cell bodies compared to neighboring mature neurons. These cells represent early lineage commitment rather than functional maturity. It is worth noting that while we observed an increase in neurogenesis (BrdU^+^/NeuN^+^), a large majority of the preserved NeuN^+^ cells in the treatment groups were BrdU^−^. This implies that our intervention exerts a dual effect: promoting acute neurogenesis and, more importantly, providing neuroprotection to preexisting mature neurons against AD-related neuronal loss. Furthermore, we identified the myokine CTSB as a critical mediator of exercise-induced cognitive benefits, potentially transported to the brain via EVs, which was validated in an APP/PS1 model. We also elucidated that OGT-dependent O-GlcNAcylation promotes CTSB expression by stabilizing it. These results preliminarily indicate that exercise plays a central role in enhancing hippocampal neurogenesis and cognitive function by modulating the muscular OGT/CTSB axis, thereby providing new targets and a theoretical foundation for exercise interventions in cognitive decline and neurodegenerative disorders.

## Conclusion

In this study, we revealed that exercise intensity and duration exhibited a dose-dependent relationship in improving memory and cognition in WT mice. Treadmill running promoted morphological improvements and neuronal differentiation in hippocampal neurons. Omics analysis and data from the UK Biobank cohort study identified the myokine CTSB as a potential key mediator of exercise-induced cognitive enhancement. Mechanistically, treadmill running may up-regulate muscle OGT-mediated O-GlcNAcylation levels, thereby promoting the secretion of CTSB into the bloodstream via EVs, ultimately improving memory and cognitive function in WT mice. Furthermore, these findings were extended to an AD mouse model. In APP/PS1 mice, treadmill running was shown to improve cognitive function, reduce amyloid plaque deposition, and attenuate neuroinflammation. Knockdown of muscle CTSB attenuated the benefits of exercise, while overexpression of muscle CTSB enhanced them. In summary, appropriate treadmill running activates the muscle OGT/CTSB signaling and facilitates the release and brain transport of CTSB via EVs, thereby enhancing hippocampal neurogenesis and cognitive function in mice. Our study further elucidates the molecular mechanisms underlying the muscle–brain axis during exercise and offers a novel theoretical framework for the prevention and management of neurodegenerative diseases through PA.

However, this study has several limitations. First, although the UK Biobank cohort data highlighted the significant mediating effect of CTSB in the improvement of cognitive function through PA, there is a lack of real-world population cohort studies—particularly multicenter cohort studies—to provide robust evidence. Second, the focus of this project on treadmill running as a single type of PA limits the generalizability of the findings regarding its impact on neurodegenerative diseases. Additional exploration is warranted to determine the influence of different exercise modalities—such as high-intensity interval training and even hypoxic training—on neurodegenerative diseases, as well as to examine the optimal exercise protocols. Third, recent studies have proposed the concept of a “multiorgan–brain axis”, emphasizing that not only skeletal muscle but also multiple peripheral organs—including the adipose tissue, liver, and spleen—collectively regulate brain metabolic and immune homeostasis through the secretion of signaling molecules and metabolites [42]. This suggests that exercise-induced cognitive improvement relies not solely on the muscle–brain axis but rather on a synergistic response across multiple systems that collectively promote brain health, indicating the need to investigate complex regulatory networks in the future. Fourth, additional evidence is required to confirm that the myokine CTSB is secreted via EVs. For instance, experiments combining EV fluorescence tracing techniques or blocking EV secretion to functionally validate this mechanism are necessary in mice. Fifth, while this study establishes the pathway through which exercise improves cognition by regulating CTSB stability via activating muscle O-GlcNAc modification and promoting its delivery to the brain via EVs, the specific cell types, receptor molecules, and downstream signaling cascades through which CTSB acts within brain tissue remain to be elucidated in future studies. Sixth, other unknown key exercise-responsive genes (such as the top DEGs identified in omics analyses) may participate in regulating the muscle–brain axis. Further research is needed to elucidate the molecular regulatory networks through which exercise improves cognitive function via the muscle–brain axis. Seventh, although our study confirms that OGT-dependent O-GlcNAcylation stabilizes the CTSB protein by inhibiting its ubiquitination-mediated degradation, the specific mechanisms (CTSB ubiquitination sites or E3 ligase recognition) remain to be elucidated. Future studies should use techniques such as mass spectrometry and gene editing to further explore how O-GlcNAcylation of CTSB inhibits its ubiquitination. Such research are expected to enhance insight into the positive impact of exercise on the brain and to inspire novel strategies for combating neurodegenerative conditions.

## Materials and Methods

### Animal experiments

The animals used in this experiment were purchased from Shanghai Model Organisms Center Inc. and housed in the specific-pathogen-free animal laboratory at Beijing Sport University. Mice were housed in standardized cages that allowed unrestricted movement, given ad libitum access to a nationally approved solid diet and water, and kept at a controlled temperature of 22 ± 2 °C with relative humidity ranging from 55% to 75%, under a regular 12-h light/12-h dark cycle. Each mouse was administered intraperitoneal injections of BrdU at 50 mg/kg, once daily for 5 consecutive days before being euthanized. Behavioral tests were conducted at each observation time point before other measurements were performed. Blood collection was achieved through cardiac puncture at the heart apex under isoflurane anesthesia. Prior to brain tissue collection, intracardiac perfusion was performed. All mice were humanely euthanized via cervical dislocation. All experimental procedures were approved by the Animal Ethics Committee of Beijing Sport University (approval no. 2021076A).

### Measurement of VO_2_max

Each set of mice underwent a 10-min warm-up on a treadmill at 6 m/min with no incline, followed by incremental speed increases of 1 m/min every 3 min until the VO_2_max threshold was achieved. The average VO_2_max value was calculated for each group. The criteria for determining VO_2_max were as follows: The mice were unable to continue running on the treadmill despite being subjected to light/electrical stimulation; oxygen uptake reached a plateau and did not change with further increases in speed; or the increase in VO_2_max between 2 consecutive stages was less than 5%. The treadmill running protocol for each group was established according to the group’s mean VO_2_max. Finally, exercise intensity and experimental groups were defined according to percentages of VO_2_max: 20%, 40%, 60%, and 80% VO_2_max groups.

### Step-down avoidance test

The step-down avoidance test was conducted using an 8-channel system (SDT-8, Chengdu Techman Co. Ltd., China) to evaluate memory performance in mice. The procedure consisted of 2 electric shock training sessions spaced 1 h apart, followed by a memory retention test 24 h later. (a) Electric shock training: Each mouse was placed on a platform. When it stepped down onto the grid floor, a foot shock (0.4 mA) was delivered. The subject was placed back into its home cage, and following a 1-h pause, the identical training protocol was carried out again. The step-down latency (recorded as latency 1) was recorded. If a mouse remained on the platform for more than 60 s, it was considered to have remembered the aversive stimulus and was placed back into the cage. If the mouse stayed on the platform for less than 60 s, the training was repeated after 1 h. Animals that still failed to meet the criterion were excluded from further testing. (b) Memory retention test: Memory retention test was performed 24 h after the first training session. The procedure was identical to that of the training phase, except that no electric shock was administered. The number of errors (i.e., instances of stepping down) and the time spent on the platform (recorded as latency 2) were measured. If a mouse did not step down within 10 min, the test was terminated, and latency 2 was recorded as 600 s. Step-down latency and number of errors were adopted as indicators of memory performance.

### Eight-arm maze test

The 8-arm maze apparatus (JLBehv-8ARMM, Shanghai Jiliang Software Science & Technology Co. Ltd., China) was adopted to evaluate the memory performance of mice in each group. Chocolate was utilized as the bait during the experiment. Prior to formal testing, the mice underwent a 4-d adaptation training period, with each session lasting 10 min per day. On day 1, 0.08 g of bait was positioned in the central area of the maze, and 3 mice were simultaneously introduced into the maze for free exploration. On day 2, 0.08 g of bait was placed both in the central area and at the entrance of each arm, and 3 mice were again allowed to explore freely. On day 3, 0.08 g of bait was positioned at the terminus of each arm, and 3 mice were introduced into the maze for free exploration. On day 4, bait was placed only at the end of arms 1, 2, 6, and 7, and individual exploration was conducted by each mouse in turn. After each adaptation session, food was provided in a restricted manner. Before the formal test, all mice were weighed and then fasted for 24 h with free access to water, until their body weight reached 80% to 85% of the prefasting weight. During testing, bait was placed only in arms 1, 2, 6, and 7, and this configuration remained unchanged throughout the experiment. The mice were permitted to roam unrestrictedly within the maze to search for and consume the chocolate bait. The test was terminated either when the mouse had explored each of the 4 baited arms or after 5 min had elapsed. After each trial, feces and any other potential cueing residues left by the mice were cleaned from the maze. Each mouse received one test per day over a 5-d period. The following parameters were recorded during the training sessions: Working memory errors, defined as reentry into a baited arm within the same trial; reference memory errors, defined as entry into an unbaited arm; and the total exploration time, the time taken for the mouse to visit all 4 baited arms. Memory performance was assessed by statistically analyzing the counts of working memory errors, reference memory errors, and the overall exploration duration.

### HE staining

After cardiac perfusion, the brain tissues were routinely embedded in paraffin and sectioned. Conventional deparaffinization and clearing were performed using xylene and a graded ethanol series. The brain tissue sections were incubated with hematoxylin solution at room temperature. Differentiation was carried out using a 1% hydrochloric acid–ethanol solution, followed by rinsing with tap water to assess the staining intensity. The sections were then treated with weak ammonia water for bluing. Subsequently, the sections were incubated with eosin staining solution and rinsed with water. The procedure continued with dehydration, clearing, and mounting steps. Afterward, the sections were examined and photographed using a light microscope.

### Nissl staining

The paraffin-embedded mouse brain tissue sections were routinely dewaxed and cleared, following the same procedure as that used for HE staining. The brain sections were immersed in Nissl staining solution for coloration. After rinsing with water, differentiation was performed using 95% alcohol. The differentiation process was monitored under a microscope to ensure clarity of the Nissl bodies. Cells exhibited a mottled blue–purple appearance under light microscopy. The sections underwent tap-water rinsing, followed by oven drying. Subsequently, dehydration, clearing, and mounting were carried out sequentially. Afterward, the sections were examined and photographed using a light microscope.

### Immunofluorescence/immunohistochemical staining

Tissue sections were subjected to conventional deparaffinization using xylene and a graded ethanol series. The samples were subjected to antigen retrieval by incubating them overnight at 60 °C under gentle conditions. Subsequently, the tissue samples underwent standard immunohistochemical staining procedures. A hydrophobic barrier was drawn around the tissue sections using an immunohistochemical pen. After treatment with 3% hydrogen peroxide, the sections were incubated with goat serum. The sections were incubated overnight at 4 °C in a humidified chamber after applying diluted primary antibodies. Following primary antibody incubation, the sections underwent a rinse in 1× phosphate-buffered saline (PBS). The appropriate secondary antibodies were added and allowed to incubate at room temperature for 50 min. For immunofluorescence staining, fluorescence-conjugated secondary antibodies were used, and an antifade mounting medium was used to mount the sections. For dual-color immunofluorescence staining, horseradish peroxidase/cyanine3-conjugated goat anti-rabbit immunoglobulin G (IgG) was used as the secondary antibody. The antibody-staining workflow was repeated by performing antigen retrieval, followed by incubation with the primary antibody and then the secondary antibody after the initial staining round. Finally, the nuclei were counterstained with 4′,6-diamidino-2-phenylindole (DAPI) and incubated for 10 min protected from light. The sections were examined and photographed under an inverted fluorescence microscope.

### Transcriptomic sequencing

Mouse muscle tissue was processed with an RNA extraction kit (catalog no. B511311, Sangon Biotech [Shanghai] Co. Ltd., China) to obtain total RNA. The RNA was assessed for integrity with 1% agarose gel electrophoresis after the removal of any genomic DNA contamination. The quality and concentration of RNA were subsequently determined for library preparation. The mRNA sequencing (mRNA-seq) libraries were prepared with the VAHTS mRNA-seq V2 Library Prep Kit for Illumina, incorporating unique index tags for each sample. Sequencing quality was subsequently assessed with FastQC (v0.11.2). After filtering, the clean reads were mapped to the reference genome using HISAT2 (v2.0). The alignment results were statistically analyzed with RSeQC (version 2.6.1). Homogeneity distribution and structural features of the genome were examined using Qualimap (version 2.2.1). Gene coverage was statistically analyzed with BEDTools (version 2.26.0). Gene expression values were calculated using StringTie (version 1.3.3b). Principal components analysis, together with principal coordinate analysis, was used to illustrate the disparities in intersample distances and expression levels. DEGs between 2 sample groups were identified using DESeq2 (version 1.12.4). Finally, functional analysis of the DEGs was conducted.

### Population-based analysis of PA and cognitive function in the UK Biobank cohort

This study used data from the UK Biobank to examine the association between PA and cognitive outcomes. The exposure was baseline PA, including LTPA and TPA, quantified in metabolic equivalent of task (MET)-minutes per week and categorized as low, moderate, or high. The primary outcome was incident dementia, identified through hospital and death registry records using validated International Classification of Diseases 10th Revision codes. Baseline cognitive function was assessed via 5 computerized tests. Multivariable Cox proportional hazards models were applied to estimate the association between PA and dementia risk, with follow-up until diagnosis, death, or end of data availability. Linear regression models evaluated cross-sectional associations between PA and cognitive performance. Mediation analyses were conducted to assess whether CTSB, DCUN1D1, GLRX, SERPINE1, and GSN mediated the relationship between PA and cognition, adjusting for age and sex. (detailed information about the methodology, variable definitions, and coding procedures can be found in Supplementary Material 1)

### ELISA assay

The levels of BDNF and CTSB in mouse serum were quantified with an ELISA kit following the supplier’s protocol. Briefly, the antibody was diluted to 1 to 10 μg/ml, and 100 μl of this solution was pipetted into each well of a 96-well plate and then left at ambient temperature for 1.5 h. After washing the plate 3 times with wash buffer, 200 μl of blocking solution was added to each well, and the plate was incubated again at room temperature for 1.5 h. After another 3 washes, the mouse serum (100 μl) was pipetted into each well and allowed to incubate at ambient temperature for 1.5 h (with blank and standard wells included in parallel). The plate was washed 3 additional times, after which 100 μl of the biotin-conjugated antibody working solution was added to each well and the plate was incubated for 1.5 h. After 3 additional washes, 100 μl of enzyme conjugate working solution was added and incubated for 1.5 h. The plate was washed 3 additional times, after which 100 μl of 3,3′,5,5′-tetramethylbenzidine substrate solution was added to each well. It was then incubated in the dark for 10 to 30 min, allowing a clear color gradient to develop in the serially diluted standard wells. Then, 100 μl of 2 M sulfuric acid solution was added. Finally, absorbance readings were obtained at 450 nm via a microplate reader.

### Culture of C2C12 cells

The C2C12 cell line (#CRL-1772, American Type Culture Collection) was cultured in basal medium (C11995500BT, Gibco, USA) that was enriched with 10% fetal bovine serum (10099-141C, Gibco, USA), penicillin (100 IU/ml), and streptomycin (100 μg/ml; 15140-122, Gibco, USA). Cell dissociation was performed using 0.25% trypsin–EDTA (25200-056, Gibco, USA). The myogenic differentiation medium contains 2% horse serum (26050-088, Gibco, USA), not 10% fetal bovine serum.

### Giemsa staining

C2C12 cells were transferred to 6-well plates and maintained in myogenic differentiation induction medium. After differentiation induction, the complete culture medium was aspirated, and the cells were washed 3 times with 1× ice-cold PBS. The cells were then fixed in 4 % paraformaldehyde for 10 min and subsequently rinsed 3 times with ice-cold 1× PBS. Finally, the cells were stained with Giemsa staining solution and observed under a light microscope for imaging.

### WB assay

The cryopreserved muscle tissues were thawed on ice. A quantity of tissue samples was weighed and then homogenized in protein lysis buffer supplemented with 1% protease inhibitor and 1% phosphatase inhibitor. Following thorough grinding of the tissues under low-temperature conditions, the samples were centrifuged at 12,000 rpm for 15 min at 4 °C. After the sample was centrifuged, the supernatant was collected, and the protein level was quantified using the bicinchoninic acid method. Separation and stacking gels were prepared. After vertical electrophoresis using SDS–polyacrylamide gel electrophoresis, the proteins were transferred horizontally onto a polyvinylidene difluoride membrane. Upon completion of the horizontal electrophoresis, the membrane was blocked on a shaker for 1 h at room temperature. The membrane was rinsed 3 times with 1× tris-buffered saline with Tween 20 (TBST), after which the diluted primary antibodies were applied and left to incubate overnight at 4 °C. Subsequently, the membrane underwent another 3 washes with 1× TBST, followed by a 2-h incubation with the appropriate secondary antibodies at room temperature on a shaker. After incubation, 3 additional washes with 1× TBST were performed. Target protein bands were visualized using enhanced chemiluminescence. All antibodies used in this work are detailed in Table 15.

**Table 15.** Antibodies

Antibodies	Sources
ACAN	Proteintech, 68350-1-Ig
O-GlcNAc	Merck, MABS157
MMP-13	Proteintech, 18165-1-AP
CTSB	Cell Signaling Technology, 31718S
CTSB	Abmart, PA6411S
OGT	Proteintech, 11576-2-AP
OGT	Abmart, T58134
Flag	Proteintech, 20543-1-AP
HA	Abmart, M20013S
Ub	Abmart, M026378S
DCX	Proteintech, 13925-1-AP
BrdU	Servicebio, GB12051-50
NeuN	Servicebio, GB11138-50
BDNF	Servicebio, GB11559-50
GFAP	Servicebio, GB12096-50
Iba1	Servicebio, GB12105-50
Calnexin	Affinity, AF5362
CD9	Affinity, AF5139
TSG101	Affinity, DF8427
CD63	Servicebio, GB115712-50
β-Actin	Proteintech, 66009-1-Ig
GAPDH	Proteintech, 60004-1-Ig
β-Tubulin	Proteintech, 10094-1-AP

O-GlcNAc, O-linked N-acetylglucosamine; OGT, O-linked N-acetylglucosaminyltransferase; CTSB, cathepsin B; HA, hemagglutinin; Ub, ubiquitin; BrdU, 5-bromo-2′-deoxyuridine; BDNF, brain-derived neurotrophic factor; GFAP, glial fibrillary acidic protein; GAPDH, glyceraldehyde-3-phosphate dehydrogenase; ACAN, aggrecan core protein; MMP-13, matrix metallopeptidase 13; DCX, doublecortin; NeuN, neuronal nuclei antigen; Iba1, ionized calcium binding adapter molecule 1; TSG101, tumor susceptibility gene 101.

### IP/co-IP

Cells were plated in 10-cm dishes, and following drug exposure, they were rinsed 3 times with ice-cold 1× PBS. Then, 500 μl of IP lysis buffer containing phosphatase inhibitors was added to each dish for complete lysis, followed by incubation on ice for 5 to 10 min (brief sonication was applied to facilitate cell lysis). The lysates were subsequently centrifuged at 10,000 rpm for 15 min at 4 °C. A 20-μl aliquot of the supernatant was collected as the input group. The remaining supernatant was mixed with primary antibodies and incubated overnight at 4 °C. The next day, 20 μl of Protein A/C magnetic beads (prewashed 3 times with 1× TBST solution) were added. The beads were captured using a magnetic stand, and the supernatant was poured off, and the washing process was repeated 3 to 5 times. Finally, the beads were resuspended in 1× loading buffer and heated to 100 °C for 8 min. Subsequent electrophoresis steps were consistent with those used in WB experiments. For exogenous co-IP experiments, antibody-conjugated nanomagnetic beads were used in place of primary antibody overnight incubation.

### Molecular docking

First, the STRING database was used to investigate potential interactions between the 2 proteins. The specific steps were as follows: (a) access the homepage of the STRING database website and enter the information of the 2 proteins, selecting “*Mus musculus*” as the species. (b) Submit the query and set parameters: Click the search button, and the system will perform the search based on the entered protein information and selected species. The default medium confidence threshold (0.4) was used as the initial parameter setting. (c) View and export the protein–protein interaction network: After the search is completed, the system displays a protein interaction map, from which the results can be downloaded and exported. Subsequently, the HDOCK server was used to perform molecular docking experiments to forecast the binding regions of the 2 molecules. The molecular visualization software PyMOL was used to open the downloaded PDB file and examine the protein–protein docking conformations. Finally, the docking scores output by the HDOCK website were recorded, and the retrieved PDB file was then uploaded to PDBePISA for additional examination. A lower binding energy indicates stronger binding affinity.

### Prediction of O-GlcNAcylation sites

The protein sequence of CTSB was retrieved from the website: https://www.ncbi.nlm.nih.gov. The protein sequences of CTSB from different species (the FASTA sequences of the proteins can be obtained from the PubMed website) were input into the O-GlcNAc modification prediction website at https://services.healthtech.dtu.dk to predict potential O-GlcNAcylation sites of CTSB.

### Y-maze test

The Y-maze apparatus consists of 3 equally long plastic arms with an angle of 120° between each pair of arms. The mouse was gently placed at the maze’s midpoint and permitted to roam unrestricted for 10 min. An arm entry was counted only when the animal’s 4 paws fully entered the arm. Both the order and the total count of arm entries were documented. A valid alternation was defined as 3 successive entries into 3 different arms. The spontaneous alternation percentage was then determined by the following equation: Spontaneous Alternation Rate (%) = [(Number of Spontaneous Alternations) / (Total Arm Entries − 2)] × 100. Between trials, the maze was cleaned with 75% ethanol to eliminate any olfactory cues that could affect behavioral performance.

### Novel object recognition test

The novel object recognition test, performed in an open box apparatus measuring 30 × 30 × 45 cm^3^, was used to assess spatial memory in rodents. In the habituation stage, each mouse was confined to the arena for 10 min daily over 3 successive days. During the testing stage, mice were again placed in the arena for 10 min with a pair of identical objects (cylinders). One hour later, the mice were placed back into the arena for an additional 10 min, during which one of the items was swapped for a new object (a cone). The time during which the mouse’s snout was within 2 cm of each object was recorded. Data from animals with a cumulative exploration duration of less than 8 s were discarded. The proportion of time the subject spent investigating the novel object was expressed as a discrimination index (DI), calculated by the formula: DI = [Time spent exploring novel object / (Time spent exploring novel object + Time spent exploring familiar object)] × 100%.

### Morris water maze

The Morris water maze test consists of 2 phases: the place navigation test and the spatial probe test. The place navigation test was performed over 5 d. The main procedures were as follows: Mice were individually placed into the water from 4 marked entry points. The time taken from entering the water to climbing onto the hidden platform was measured as the escape latency. A mouse was considered to have found the hidden platform if it remained on the platform for 10 s (at which point the camera automatically terminated the recording session). The maximum allowed time for locating the hidden platform was 60 s. If a mouse did not locate the platform within 60 s, the trial was terminated, and the escape latency was logged as 60 s. The mouse was then directed to the hidden platform and allowed to remain there for 10 s. This guidance procedure was implemented to reinforce the mouse’s learning and memory of the environmental cues surrounding the platform, thereby facilitating quicker localization of the platform in subsequent trials. One day prior to the commencement of the Morris water maze test, all mice were introduced into the circular pool without the platform and allowed to swim unrestricted for 10 min to acclimate to the experimental environment. After each trial, every mouse was dried with a towel and a hairdryer prior to returning them to their home cages. The day after the place navigation test was completed, a spatial probe assessment was administered to every group. Prior to this assessment, the hidden platform located in the first quadrant was taken away. Similarly, each mouse was introduced into the water from the 4 designated entry points, with each trial lasting 60 s. All swimming paths of the mice were documented using the camera. The number of platform crossings, total swimming distance, and the percentage of time and distance spent in the target quadrant (where the platform had been located) were measured to evaluate spatial memory performance. After each trial, every mouse was dried with a towel and a hairdryer before being returned to its home cage.

### Patch–clamp detection

#### Whole-cell recording

(a) Recording of mEPSCs. The pipette internal solution for mEPSCs was prepared. During whole-cell recording, 1 μM tetrodotoxin was incorporated into the perfusion solution to block sodium channels. Under voltage-clamp mode, the CA1 region of the hippocampus was located using an optical microscope. Pyramidal neurons were clamped using a glass microelectrode filled with the internal solution (electrode resistance, 5 to 8 MΩ). After forming a high-resistance seal (gigohm range), the membrane was ruptured to establish whole-cell mode, and the membrane potential was held constant at −70 mV. Recordings were performed in gap-free mode for 3 to 5 min, with 2 recordings per cell at 2- to 3-min intervals. Acquired data were analyzed using Mini Analysis software, and the frequency and amplitude of mEPSCs were recorded. (b) Recording of mIPSCs: The pipette internal solution for mIPSCs was prepared. Recordings were performed by clamping the voltage at 0 mV. Under voltage-clamp mode, using the same adjustment method as for mEPSC recordings, the acquired data were analyzed using Mini Analysis software, and the frequency and amplitude of mIPSCs were recorded.

#### Field potential recording

The hippocampal slices, after incubation, were placed in the recording chamber where they were continuously superfused with oxygen-saturated artificial cerebrospinal fluid at a flow rate of 2 to 3 ml/min. All recordings were conducted at room temperature. Excitatory postsynaptic potentials were recorded in the CA1 stratum radiatum after stimulating the Schaffer collateral pathway. Electrical stimulation was delivered via a concentric bipolar stimulating electrode placed near the CA3 region, and fEPSPs were recorded in the CA1 region. Stimuli were delivered at 20-s intervals. The recording electrode was filled with 4 M NaCl and had a resistance of 1 to 3 MΩ. The stimulus intensity was adjusted to maintain an initial fEPSP amplitude of approximately 0.5 to 1.0 mV. After stabilization for at least 10 min, subsequent procedures were conducted.

#### LTP recording

fEPSPs were evoked via the stimulating electrode. After the fEPSP amplitude had stabilized for at least 10 min, stimulus intensities of 50, 100, 200, 300, 400, 500, 600, 700, and 800 μA were applied sequentially until the maximum fEPSP amplitude was reached. A stimulus intensity yielding 30% of the maximum fEPSP amplitude was selected as the baseline. After stable baseline recording for at least 20 min, high-frequency stimulation (100 Hz, 2 trains, 30-s intertrain interval) was delivered. Following high-frequency stimulation, baseline recording was resumed and continued for 1 h.

### Thioflavin S staining

Thioflavin S staining was performed as directed by the manufacturer’s instructions. Briefly, the paraffin-embedded mouse brain tissue sections were routinely dewaxed and cleared, following the same procedure as that used for HE staining. Brain tissue sections were immersed in a 0.3% Thioflavin S solution (prepared in 50% ethanol) and incubated for 5 to 8 min in the dark. The sections were then rinsed with 80% ethanol for 10 s, followed by a 10-s dip in distilled water and one rinse with distilled water. Subsequently, the sections were treated with DAPI staining solution and incubated for 10 min protected from light. Each section underwent three 5-min washes in 1× PBS on a shaking platform. The sections were ultimately mounted with an antifade medium. Imaging was carried out under a laser scanning confocal fluorescence microscope.

### Glycine silver immersion plating nerve staining

Glycine silver immersion plating nerve staining was performed as directed by the manufacturer’s instructions. Briefly, the paraffin-embedded mouse brain tissue sections were routinely dewaxed and cleared, following the same procedure as that used for HE staining. Then, the sections were stained with silver glycinate solution C for 5 min and then with silver glycinate solution B for 5 min, followed by staining with silver glycinate solution A I (prewarmed to 45 °C in advance) for several seconds and subsequently with silver glycinate solution A II (also prewarmed to 45 °C in advance) for several seconds. Distilled water was used to rinse the sections thereafter. If the background appeared too dark, the sections were treated with silver glycinate solution D for 1 s and rinsed 3 times with distilled water. After conventional dehydration and clearing, the sections were fixed with a neutral-resin medium. Observation and imaging were performed using a light microscope.

### Extraction of EVs from muscle tissue

EVs were isolated from mouse muscle tissue using ultracentrifugation. The muscle tissue was minced into approximately 1-mm^3^ fragments using ophthalmic surgical scissors and transferred into a 50-ml centrifuge tube. After adding an equal volume of the targeted enzymatic digestion solution to the tube, it was kept at 37 °C in a temperature-controlled incubator with gentle rocking for 2 to 3 h. After the suspension turned turbid, the tube was removed from the incubator. The digestion was stopped by introducing Dulbecco’s modified Eagle’s medium (DMEM) supplemented with 10% exosome-depleted serum. The mixture was centrifuged at 2,000*g* for 5 min at 4 °C, after which the supernatant was transferred to a fresh 50-ml centrifuge tube. A series of gradient centrifugation steps was then performed. The supernatant was centrifuged at 2,000*g* for 30 min at 4 °C, after which the supernatant was harvested. Subsequently, it was centrifuged at 10,000*g* for 45 min at 4 °C. The supernatant was then removed, and the remaining fraction was passed through a 0.45-μm filter to eliminate larger EVs. The filtrate was collected and subjected to ultracentrifugation at 100,000*g* for 70 min at 4 °C. After ultracentrifugation, the supernatant was discarded, and the pellet was retained. The pellet was resuspended in 10 ml of ice-cold 1× PBS and ultracentrifuged again at 100,000*g* for 70 min at 4 °C. The resulting pellet, containing muscle tissue-derived EVs, was resuspended in 200 μl of 1× PBS. The concentration and size of EVs were determined using the NanoAnalyzer system (NanoFCM, China). Calibration was performed using 68- to 155-nm silica nanospheres. Imaging of the EVs was conducted using transmission electron microscopy (Talos F200, Thermo Fisher Scientific, USA).

### Gene editing experiments (in vitro and in vivo)

#### Cell transfection

The siRNA targeting the OGT gene was obtained from HeYuan Biotechnology Co. Ltd. (China). The transfection mixture was made by dissolving 4 μg of plasmid DNA (or 50 nM siRNA) in 250 μl of DMEM basal medium within a 1.5-ml centrifuge tube. In a separate tube, 10 μl of LipoFiter 3.0 (HANBIO, HB-TRLF3-1000). was mixed with another 250 μl of DMEM basal medium. After gently combining the 2 solutions, the mixture was allowed to stand at room temperature for 20 min. Finally, 500 μl of this preparation was transferred to each well of a 6-well plate, and 1.5 ml of complete growth medium was added to each well. In this study, the target sequence for OGT knockdown was GCACAGCUCUGAAACUUAAGCTT; the target sequence for CTSB knockdown was AGGTGCAACAAGAGCTGTGAA; the plasmid for CTSB overexpression was pCDNA3.1-CMV-MCS-3HA-EF1-Puro; the plasmid for OGT-OE was pHBLV-CMV-MCS-3flag-EF1-puro; and the 4 point-mutation plasmids for CTSB were pCMV-Ctsb(mouse)-T183A-3×FLAG-P2A-CopGFP, pCMV-Ctsb(mouse)-S194A-3×FLAG-P2A-CopGFP, pCMV-Ctsb(mouse)-T199A-3×FLAG-P2A-CopGFP, and pCMV-Ctsb (mouse)-T204A-3×FLAG-P2A-CopGFP.

#### Recombinant AAV construction for animal experiments

Recombinant AAVs for OGT-OE, CTSB overexpression, and CTSB knockdown were constructed using standard molecular cloning procedures. Briefly, the following vectors were used: GV526 (CAG-MCS-SV40 PolyA) for OGT-OE, GV411 (CMV-betaGlobin-MCS-3Flag-SV40 PolyA) for CTSB overexpression, and GV478 (U6-MCS-CAG-EGFP) for CTSB knockdown. The procedures included vector digestion, retrieval of the target gene fragment, ligation of annealed products into the vector, and transformation into competent cells. Positive clones were identified and cultured in LB medium. The resulting bacterial cultures were sequenced and aligned with the target gene sequences. Correct clones were selected for plasmid extraction to be used in subsequent AAV packaging. AAV packaging was performed using the AAV Helper-Free System, which involves cotransfection of human embryonic kidney 293T cells with 3 plasmids: the viral vector, pAAV-RC, and pHelper. After 6 h of transfection, the culture medium was refreshed with complete growth medium. Viruses were harvested 72 h posttransfection. The harvested viruses were purified by gradient ultracentrifugation and concentrated using ultrafiltration columns to obtain high-purity AAV preparations. Finally, viral titers were determined using qPCR.

### Statistical analysis

Experimental results are expressed as the means ± standard deviation ($\bar{x}$ ± *S*). Statistical analysis was performed using SPSS 25.0 software. GraphPad Prism 10 was used for graphing statistical data. In the mouse experiments involving treadmill running at different intensities, data from the 8-arm maze test were analyzed using repeated-measures analysis of variance (ANOVA), while the remaining data were analyzed using 2-way ANOVA to determine whether each factor exerted an independent effect or interactions. This was followed by one-way ANOVA to assess differences among groups. In the remaining experiments, an independent *t* test was applied for pairwise group comparisons, while one-way ANOVA together with the least significant difference post hoc test was used for analyses involving multiple groups. Results were deemed statistically significant when the *P* value was below 0.05.

## Ethical Approval

Approval has been given by the Ethical Committee of Beijing Sport University (approval no. 2021076A) on the Care and Use of Animal Subjects in Research. This study was according to the Helsinki Declaration. The UK Biobank data were obtained with approval from the North West Multi-center Research Ethics Committee. This research was conducted using resources from the UK Biobank under approved application number 312092.

## Data Availability

The data presented in this study are available from the corresponding author upon request.

## Supplementary Materials

Supplementary 1 Supplementary Text Figs. S1 to S9 Raw Data of WB
