# Supplementary material for: Myokine Cathepsin B as a Key Muscle–Brain Axis Regulator Mediates Treadmill-Running-Induced Hippocampal Neurogenesis and Cognitive Improvement in Mice
Source: Research (Wash D C). 2026 Apr 23;9:1233. doi: 10.34133/research.1233 (PMC13103465; doi:10.34133/research.1233)
Supplement: Supplementary 1 — Supplementary Text Figs. S1 to S9 Raw Data of WB [file research.1233.f1.zip › Supplementary_Materials.pdf]

Supplementary Materials for

Myokine CTSB as a key muscle-brain axis regulator mediates exercise-induced hippocampal neurogenesis and cognitive improvement in mice

Xuchang Zhou[1#], Dongxue Wang[2#], Huili Deng[3#], Jianmin Guo[4#], Xier Chen[1,5], Zhangyu Lin[1,5], Baolong Liu[6], Ruobing Zhao[7], Lu Gao[1], Xuan Yin[2], Yun Zhang[1,8], Yan Chen[1], Yajing Yang[1,9], Qingxian Li[10], Qu Shen[3], Jianguang Ji[2*], Guoxin Ni[1*]

[1]Department of Rehabilitation Medicine, the First Affiliated Hospital of Xiamen University, School of Medicine, Xiamen University, Xiamen 361003, China

[2]Faculty of Health Sciences, University of Macau, Macau SAR 999078, China

[3]School of Medicine, Xiamen University, Xiamen 361102, China

[4]Institute of Integrated Bioinfomedicine and Translational Science (IBTS), School of Chinese Medicine, Hong Kong Baptist University, Hong Kong 999077, China

[5]School of Sports Medicine, Wuhan Sports University, Wuhan 430079, China

[6]Department of Developmental Cell Biology, Key Laboratory of Cell Biology, Ministry of Public Health, China Medical University, Shenyang 110122, China

[7]Department of Exercise Physiology, Beijing Sport University, Beijing, 100084, China

[8]Medical college, Guangxi University, Nanning, 53004, China

[9]College of Acupuncture-Moxibustion and Orthopedics, Hubei University of Chinese Medicine, Wuhan 430061, China

[10]Department of Orthopedic Surgery, the First Affiliated Hospital of Xiamen University, School of Medicine, Xiamen University, Xiamen 361003, China.

* Correspondence: jianguangji@um.edu.mo (Jianguang Ji) and nigx@xmu.edu.cn (Guoxin Ni)

† Xuchang Zhou, Dongxue Wang, Huili Deng, and Jianmin Guo contributed equally to this work

This PDF file includes:

Supplementary Text: UK Biobank cohort analysis
Figs. S1 to S9
Raw Data of WB

Supplementary Text

UK Biobank cohort analysis

Supplementary method

Study population

Data were obtained from the UK Biobank, a large-scale population-based cohort with over 500,000 individuals aged 37–73 years who were recruited between 2006 and 2010. At the assessment center, participants reported their demographics, socioeconomic status, and lifestyle factors. In addition, they underwent physical examinations and consented to being followed through record linkage. Further information regarding this cohort can be found at http://www.ukbiobank.ac.uk/.

A total of 502,357 participants were recruited into the UK Biobank cohort between 2006 and 2010. After excluding individuals with missing information on any covariates (n = 104,488), prevalent dementia at baseline (n = 104), missing leisure-time PA (LTPA) measurements (n = 27,408), and missing total physical activity (TPA) measurements (n = 1,182), the final sample sizes were 370,357 participants for the analysis of LTPA and 395,953 for the analysis of TPA. Further analyses were conducted to investigate the association between physical activity (PA) and cognitive decline, with cognitive assessments performed at baseline. (Supplementary Table S3).

Exposure measurement

Participants' PA was collected at baseline, which included LTPA and TPA. LTPA included five activities undertaken in the last four weeks: walking for pleasure, light DIY (do-it-yourself), heavy DIY, strenuous sports, and other exercises. Participants were asked to quantify the frequency and duration of their participation, which was converted to an average weekly value. A metabolic equivalent (MET) value was assigned to walking for pleasure (3.5), light DIY (1.5), heavy DIY (5.5), strenuous sports (8.0) other exercises (4.0), and none of the five activities (0). The frequency (once in the last 4 weeks, 2-3 times in the last 4 weeks, once a week, 2-3 times a week, 4-5 times a week or every day) and duration (< 15 minutes, between 15 and 30 minutes, between 30 minutes and 1 hour, between 1 and 1.5 hours, between 1.5 and 2 hours, between 2 and 3 hours, or > 3 hours) of LTPA were recorded, and each category was assigned to specific value to facilitate calculation (Supplementary Table S1). Similarly, TPA which was a modified version of the International Physical Activity Questionnaire (IPAQ), was assessed through capturing the frequency and duration of walking, moderate, and vigorous PA performed over the last seven days. Data were analysied in accordance with the IPAQ scoring protocol. MET levels included walking (3.3), moderate-intensity (4.0), and vigorous-intensity (8.0). For both the measures, total weekly PA (MET-mins/week) was calculated by multiplying the frequency, duration, and the MET values. Based on the standard scoring criteria of PA, we grouped the sample as low PA (<600 MET-mins/week), moderate PA (600 to <3000 MET-mins/week), and high PA (≥3000 MET-mins/week); the threshold at 600 MET-mins/week is equivalent to reaching the recommended guidelines (150 minutes per week) for moderate-intensity PA.

Identification of dementia

Dementia cases were identified using hospital inpatient admissions and death registry records. Inpatient admissions records were available from the Hospital Episode Statistics for England, the Scottish Morbidity Record for Scotland, and the Patient Episode Database for Wales. Death registry records were available from the NHS England for England and Wales, and the Information and Statistics Division for Scotland. Primary and secondary hospital diagnoses and causes of death were recorded using the International Classification of Diseases (ICD-10) coding

system. The ICD codes used to ascertain dementia were selected and validated by the UK Biobank outcome adjudication group (Supplementary Table S2)[1].

Cognitive function assessment

Cognitive testing in the UKB was conducted via touchscreen assessments. Referring to previous research [2], we included five cognitive tests administered during the Initial Assessment Visit (2006–2010): (1) UKB Reaction Time Test: This test measured the time it took for participants to accurately identify matching pairs, and the outcome measure was the mean time to correctly identify matches. (2) UKB Pairs Matching Test: This test assessed participants' ability to match pairs correctly, and the outcome measure was the number of incorrect matches made. (3) UKB Fluid Intelligence: Fluid intelligence was evaluated by the number of questions answered correctly within a 2-min time frame. (4) UKB Prospective Memory: This test assessed prospective memory, focusing on the accuracy of participants' recall. (5) UKB Numeric Memory: Numeric memory was assessed based on the longest number correctly recalled during the numeric memory test. Detailed information about these tests is available on the UKB official website.

Plasma proteomic measurement

The baseline blood samples were collected between 2007 and 2010, followed by subsequent sample processing utilizing the antibody-based Olink Explore Proximity Extension Assay for standardized quantification. Detailed protocols regarding sample handling, plasma analysis using Olink proteomics detection methods, data processing, and quality control procedures have been thoroughly described in previous publications[3] .

Covariates

Covariates included sociodemographic(age, sex, ethnicity, education level, and socioeconomic status (Townsend deprivation index)), lifestyle (smoking status, body mass index (BMI) and alcohol status), and genetic factors (*APOE*-ε4 carrier status) previously associated with dementia and are considered here to be potential confounders in determining the relationship between the exposure and outcome[4].

Statistical analysis

Descriptive statistics were used to compare baseline characteristics between participants with and without dementia. For normally distributed variables, means and standard deviations were reported, while medians and interquartile ranges were calculated for skewed variables. Multivariable Cox proportional hazards models were employed to estimate the association between PA and incident dementia. Follow-up time (in years) was calculated from the enrollment assessment date to the date of the first dementia diagnosis, death, loss to follow-up, or end of follow-up—whichever occurred first. The end of follow-up was defined based on the availability of electronic health record data in the UK Biobank: September 30, 2021, for England; July 31, 2021, for Scotland; and February 28, 2018, for Wales. The proportional hazards assumption was assessed visually using scaled Schoenfeld residuals, and no violations were observed. Three models were constructed for the main analyses: Model 1 was the crude model; Model 2 was adjusted for age, sex, education, and APOE-ε4 carrier status; and Model 3 was fully adjusted for age, sex, ethnicity, education, Townsend deprivation index, BMI, smoking status, alcohol consumption, family history of dementia, and APOE-ε4 carrier status. Additionally, linear regression models were used to examine the cross-sectional association between PA and baseline cognitive function, with adjustments consistent with the Cox models. Mediation analyses were performed to determine whether CTSB mediated the relationship between PA and improved cognitive function, with adjustment limited to age and sex.

1. Wilkinson, T., et al., *Identifying dementia outcomes in UK Biobank: a validation study of primary care, hospital admissions and mortality data.* Eur J Epidemiol, 2019. **34**(6): p. 557-565.
2. Yang, K., et al., *Residential blue space, cognitive function, and the role of air pollution in middle-aged and older adults: A cross-sectional study based on UK biobank.* Ecotoxicol Environ Saf, 2024. **288**: p. 117355.
3. Sun, B.B., et al., *Plasma proteomic associations with genetics and health in the UK Biobank.* Nature, 2023. **622**(7982): p. 329-338.
4. Livingston, G., et al., *Dementia prevention, intervention, and care: 2020 report of the Lancet Commission.* Lancet, 2020. **396**(10248): p. 413-446.

Supplementary Figures Legends

Fig. S1 Effects of treadmill exercise at different intensities and durations on hippocampal morphology in WT mice. (A: HE staining of hippocampal tissue in WT mice; B: Nissl staining of hippocampal tissue in WT mice; 2W group: 2-week treadmill exercise, 4W group: 4-week treadmill exercise, 8W group: 8-week treadmill exercise, Q group: Sedentary control group, 0.2 group: 20% $\dot{V}O_2max$ treadmill exercise, 0.4 group: 40% $\dot{V}O_2max$ treadmill exercise, 0.6 group: 60% $\dot{V}O_2max$ treadmill exercise, 0.8 group: 80% $\dot{V}O_2max$ treadmill exercise, n=6)

Fig. S2 Treadmill exercise promotes the release of the myokine CTSB. (A: Exercise protocol for human subjects performing a single session of exercise at different intensities; B: Volcano plot of differentially expressed proteins in the blood proteome following a single session of moderate-intensity exercise in human subjects; C: Volcano plot of differentially expressed proteins in the blood proteome following a single session of high-intensity exercise in human subjects; D: Expression of CTSB protein in the muscle tissue of WT mice detected by immunofluorescence staining; E: Expression of CTSB and BDNF proteins in the hippocampal tissue of WT mice detected by immunofluorescence staining; F: Expression of CTSB protein in the serum of WT mice detected by ELISA; G: Expression of BDNF protein in the serum of WT mice, as detected by ELISA; n=6, *P<0.05, **P<0.01, Fig S2A-C was adapted and modified from [Guseh et al., 2020, Scientific reports, doi:10.1038/s41598-020-67669-0], used under CC BY 4.0 [https://creativecommons.org/licenses/by/4.0/]. Changes were made to emphasize CTSB protein among the differentially expressed proteins.)

Fig. S3 Potential regulation of CTSB by O-GlcNAcylation/Ubiquitination in C2C12 cells. (A: Prediction of potential O-GlcNAcylation sites in human CTSB protein using NetOGlyc-4.0; B: Prediction of potential O-GlcNAcylation sites in mouse CTSB protein using NetOGlyc-4.0; C: Prediction of potential O-GlcNAc modification sites in rat CTSB protein using NetOGlyc-4.0; D: Giemsa staining of C2C12 cells at different time points during myoblast differentiation; E: WB analysis of total O-GlcNAcylation levels and CTSB protein expression after treatment with different concentrations of TMG; F: WB analysis of CTSB protein expression after treatment with different concentrations of CHX; G: WB analysis of CTSB protein expression after treatment with different concentrations of MG132; n=3, *P<0.05, **P<0.01)

Fig. S4 Co-localization of CTSB and OGT in mouse skeletal muscle tissue by immunofluorescence. (A: Immunofluorescence co-localization analysis of CTSB and OGT proteins in mouse skeletal muscle tissue overexpressing CTSB; B: Immunofluorescence co-localization analysis of CTSB and OGT proteins in mouse skeletal muscle tissue overexpressing OGT)

Fig. S5 Effects of knockdown or overexpression of CTSB expression in muscle tissue on pathological changes in brain tissue of APP/PS1 mice. (A: HE staining of brain tissue in APP/PS1 mice; B: Nissl staining of brain tissue in APP/PS1 mice; C: Thioflavin S staining of brain tissue in APP/PS1 mice; D: Silver staining of brain tissue in APP/PS1 mice; E: Immunohistochemical staining of GFAP protein expression in brain tissue of APP/PS1 mice; F: Immunohistochemical staining of Iba1 protein expression in brain tissue of APP/PS1 mice; G: Statistical analysis of GFAP protein expression in APP/PS1 mouse brain tissue; H: Statistical

analysis of Iba1 protein expression in APP/PS1 mouse brain tissue; I: CTSB and BDNF protein expression in APP/PS1 mouse blood was measured via ELISA; n=6, *P<0.05, **P<0.01)

Fig. S6 CTSB T199 site mutation reduces protein stability. (A-B: WB analysis of the effects of CTSB T199A mutation combined with MG132 treatment on CTSB protein expression in C2C12 cells; C and E: WB analysis of the effects of CTSB T199A mutation combined with CHX treatment on CTSB protein expression in C2C12 cells; D and F: WB analysis of the effects of CTSB T199A mutation combined with CHX/OSMI-1 treatment on CTSB protein expression in C2C12 cells; G: IP assay to detect the effect of CTSB T199A mutation combined with MG132/ OSMI-1 treatment on CTSB ubiquitination; n=3, *P<0.05, **P<0.01)

Fig. S7 Myoblast-derived EVs may ameliorate Aβ-induced hippocampal neuronal cell injury by delivering CTSB. (A: CCK-8 assay to assess the effect of different concentrations of AIACR on C2C12 cells; B: WB analysis of the effects of AIACR on total O-GlcNAcylation levels and CTSB protein expression in C2C12 cells at different time points; C: WB detection of EV protein markers; D: Nanoparticle flow cytometry analysis of the size distribution of isolated myoblast-derived EVs; E: TEM analysis of the morphology and size of isolated myoblast-derived EVs; F: IF assay to determine whether PKH26-labeled EVs were internalized by C2C12 cells; G: RT-qPCR analysis of the effects of different concentrations of myoblast-derived EVs on the expression of genes related to proliferation and synaptic plasticity in Aβ-treated HT22 cells; H: IF assay to detect the effects of different concentrations of myoblast-derived EVs on ROS accumulation in Aβ-treated HT22 cells; I: Flow cytometry analysis of the effects of different concentrations of myoblast-derived EVs on apoptosis in Aβ-treated HT22 cells; n=3, *P<0.05, ** P<0.01)

Fig. S8 Quantitative analysis of Figures 4K, 4T, and 4U. (A: WB analysis of changes in CTSB p rotein expression after treatment with MG132, CQ, and CHX; B: IP analysis indicating that ubiq uitination-mediated degradation of CTSB protein is regulated by OSMI-1; C: Four point mutatio n plasmids were constructed to validate potential O-GlcNAcylation sites on the CTSB protein; n =3, *P<0.05, **P<0.01)

Fig. S9 Quantitative analysis of BrdU/DCX, BrdU/NeuN, BrdU/NeuN, and BDNF/CTSB. (A: Effects of different treadmill exercises on BrdU/DCX cells in the hippocampus of WT mice; B-C: Effects of different treadmill exercises on BrdU/NeuN and BrdU-/NeuN cells in the hippocam pus of WT mice; D: The effects of CTSB protein knockdown on BrdU/DCX cells in the hippoca mpus of WT mice; E-F: The effects of CTSB protein knockdown on BrdU/NeuN and BrdU-/Neu N cells in the hippocampus of WT mice; GOGT protein overexpression: The effects of on BrdU/ DCX cells in the hippocampus of WT mice; H-I: The effects of OGT protein overexpression on BrdU/NeuN and BrdU-/NeuN cells in the hippocampus of WT mice; J: The effects of knockdow n/overexpression of CTSB Protein on BrdU/DCX cells in the hippocampus of APP/PS1 Mice; K-L: The effects of knockdown/overexpression of CTSB Protein on BrdU/NeuN and BrdU-/NeuN cells in the hippocampus of APP/PS1 Mice; M: The effect of CTSB protein knockdown on BDN F protein expression in the hippocampus of WT mice; N: The effect of CTSB protein knockdown on CTSB protein expression in the hippocampus of WT mice; O: The effect of OGT protein ove rexpression on BDNF protein expression in the hippocampus of WT mice; P: The effect of OGT protein overexpression on CTSB protein expression in the hippocampus of WT mice; Q: The effe cts of knockdown/overexpression of CTSB Protein on BDNF protein expression in the hippocam pus of APP/PS1 Mice; R: The effects of knockdown/overexpression of CTSB Protein on CTSB p rotein expression in the hippocampus of APP/PS1 Mice; n=6, *P<0.05, **P<0.01)

Fig. S1 Effects of treadmill running at different intensities and durations on hippocampal morphology in WT mice. (A: HE staining of hippocampal tissue in WT mice; B: Nissl staining of hippocampal tissue in WT mice; 2W group: 2-week treadmill running, 4W group: 4-week treadmill running, 8W group: 8-week treadmill running, Q group: Sedentary control group, 0.2 group: 20% VO_2max treadmill running, 0.4 group: 40% VO_2max treadmill running, 0.6 group: 60% VO_2max treadmill running, 0.8 group: 80% VO_2max treadmill running, n=6)

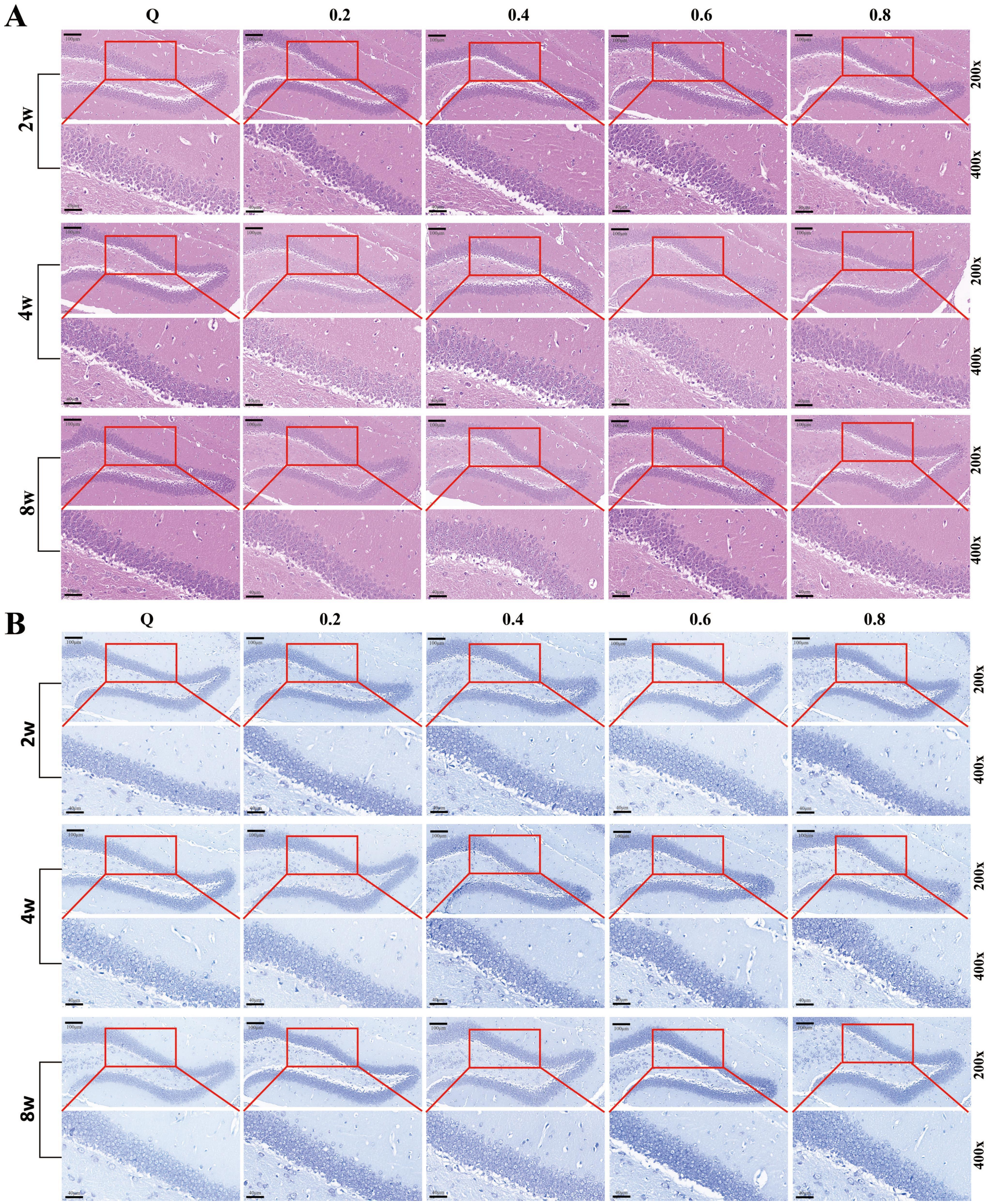
A
Q
0.2
0.4
0.6
0.8
2w
4w
8w
200x
400x
B
Q
0.2
0.4
0.6
0.8
2w
4w
8w
200x
400x

Fig. S2 Treadmill running promotes the release of the myokine CTSB. (A: Exercise protocol for human subjects performing a single session of exercise at different intensities; B: Volcano plot of differentially expressed proteins in the blood proteome following a single session of moderate-intensity exercise in human subjects; C: Volcano plot of differentially expressed proteins in the blood proteome following a single session of high-intensity exercise in human subjects; D: Expression of CTSB protein in the muscle tissue of WT mice detected by immunofluorescence staining; E: Expression of CTSB and BDNF proteins in the hippocampal tissue of WT mice detected by immunofluorescence staining; F: Expression of CTSB protein in the serum of WT mice detected by ELISA; G: Expression of BDNF protein in the serum of WT mice, as detected by ELISA; n=6, *P<0.05, **P<0.01, Fig S2A-C was adapted and modified from [Guseh et al., 2020, Scientific reports, doi:10.1038/s41598-020-67669-0], used under CC BY 4.0 [https://creativecommons.org/licenses/by/4.0/]. Changes were made to emphasize CTSB protein among the differentially expressed proteins.)

A

Intensity dependent physiological and performance metrics	Moderate intensity	High intensity
Average treadmill speed (miles per hour)	6±0	9.0±0.9
Average heart rate (beats per minute)	148±16	180±8
Average heart rate, % maximum	76±9	92±4
Heart rate, final mile (beats per minute)	150±16	187±7
Average HR as % of HR at ventilatory threshold	81±10%	100±4%
Final mile heart rate as % of HR at ventilatory threshold	82±10%	102±5%
Running time (min)	50±0	33±4

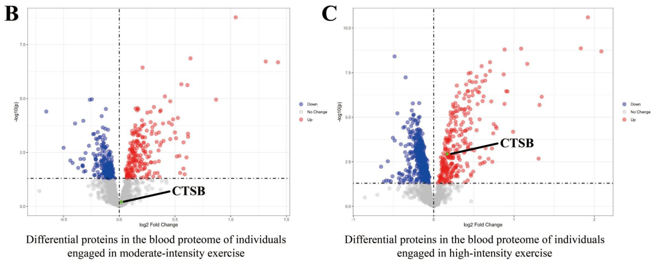

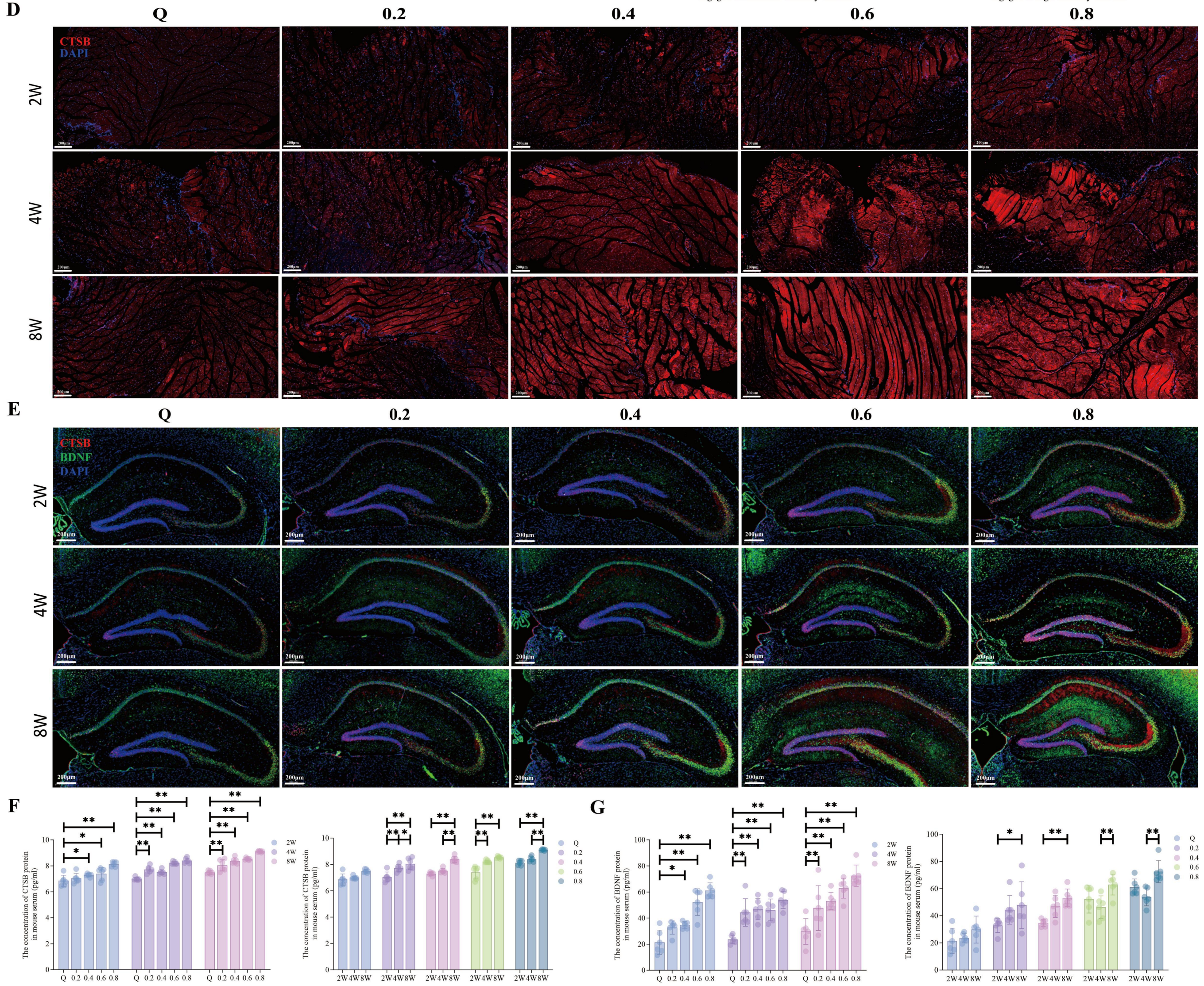

Fig. S3 Potential regulation of CTSB by O-GlcNAcylation/Ubiquitination in C2C12 cells. (A: Prediction of potential O-GlcNAcylation sites in human CTSB protein using NetOGlyc-4.0; B: Prediction of potential O-GlcNAcylation sites in mouse CTSB protein using NetOGlyc-4.0; C: Prediction of potential O-GlcNAc modification sites in rat CTSB protein using NetOGlyc-4.0; D: Giemsa staining of C2C12 cells at different time points during myoblast differentiation; E: WB analysis of total O-GlcNAcylation levels and CTSB protein expression after treatment with different concentrations of TMG; F: WB analysis of CTSB protein expression after treatment with different concentrations of CHX; G: WB analysis of CTSB protein expression after treatment with different concentrations of MG132; n=3, *$P<0.05$, **$P<0.01$)

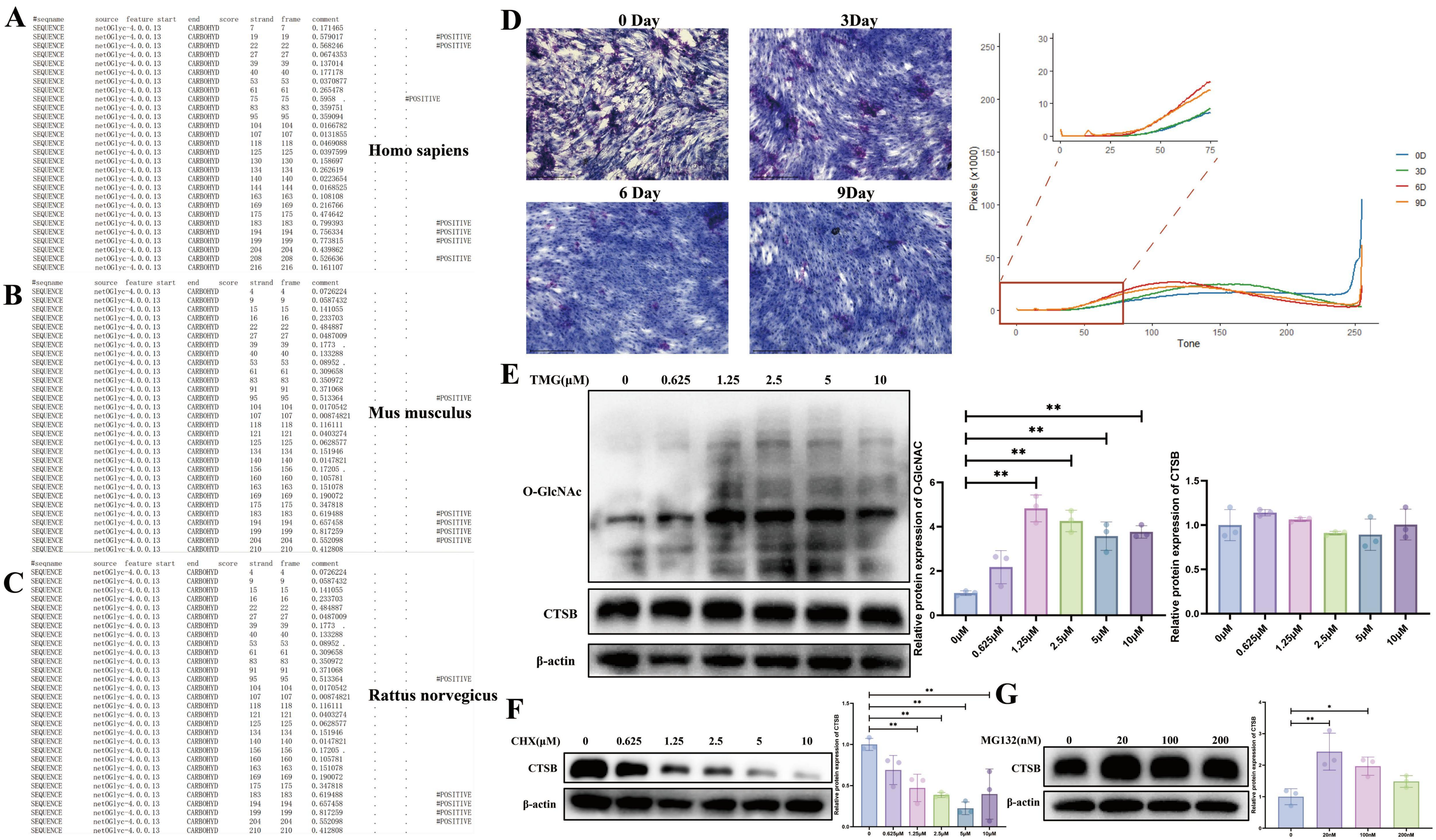

A
#seqname source feature start end score strand frame comment
SEQUENCE netOGlyc-4.0.0.13 CARBOHYD 7 7 0.171465 . .
SEQUENCE netOGlyc-4.0.0.13 CARBOHYD 19 19 0.579017 . . #POSITIVE
SEQUENCE netOGlyc-4.0.0.13 CARBOHYD 22 22 0.568246 . . #POSITIVE
SEQUENCE netOGlyc-4.0.0.13 CARBOHYD 27 27 0.0674353 . .
SEQUENCE netOGlyc-4.0.0.13 CARBOHYD 39 39 0.137014 . .
SEQUENCE netOGlyc-4.0.0.13 CARBOHYD 40 40 0.177178 . .
SEQUENCE netOGlyc-4.0.0.13 CARBOHYD 53 53 0.0370877 . .
SEQUENCE netOGlyc-4.0.0.13 CARBOHYD 61 61 0.265478 . .
SEQUENCE netOGlyc-4.0.0.13 CARBOHYD 75 75 0.5958 . . #POSITIVE
SEQUENCE netOGlyc-4.0.0.13 CARBOHYD 83 83 0.359751 . .
SEQUENCE netOGlyc-4.0.0.13 CARBOHYD 95 95 0.359094 . .
SEQUENCE netOGlyc-4.0.0.13 CARBOHYD 104 104 0.0166782 . .
SEQUENCE netOGlyc-4.0.0.13 CARBOHYD 107 107 0.0131855 . .
SEQUENCE netOGlyc-4.0.0.13 CARBOHYD 118 118 0.0469088 . .
SEQUENCE netOGlyc-4.0.0.13 CARBOHYD 125 125 0.0397599 . .
SEQUENCE netOGlyc-4.0.0.13 CARBOHYD 130 130 0.158697 . .
SEQUENCE netOGlyc-4.0.0.13 CARBOHYD 134 134 0.262619 . .
SEQUENCE netOGlyc-4.0.0.13 CARBOHYD 140 140 0.0223654 . .
SEQUENCE netOGlyc-4.0.0.13 CARBOHYD 144 144 0.0168525 . .
SEQUENCE netOGlyc-4.0.0.13 CARBOHYD 163 163 0.108108 . .
SEQUENCE netOGlyc-4.0.0.13 CARBOHYD 169 169 0.216766 . .
SEQUENCE netOGlyc-4.0.0.13 CARBOHYD 175 175 0.474642 . .
SEQUENCE netOGlyc-4.0.0.13 CARBOHYD 183 183 0.799393 . . #POSITIVE
SEQUENCE netOGlyc-4.0.0.13 CARBOHYD 194 194 0.756334 . . #POSITIVE
SEQUENCE netOGlyc-4.0.0.13 CARBOHYD 199 199 0.773815 . . #POSITIVE
SEQUENCE netOGlyc-4.0.0.13 CARBOHYD 204 204 0.439862 . .
SEQUENCE netOGlyc-4.0.0.13 CARBOHYD 208 208 0.526636 . . #POSITIVE
SEQUENCE netOGlyc-4.0.0.13 CARBOHYD 216 216 0.161107 . .
Homo sapiens
B
#seqname source feature start end score strand frame comment
SEQUENCE netOGlyc-4.0.0.13 CARBOHYD 4 4 0.0726224 . .
SEQUENCE netOGlyc-4.0.0.13 CARBOHYD 9 9 0.0587432 . .
SEQUENCE netOGlyc-4.0.0.13 CARBOHYD 15 15 0.141055 . .
SEQUENCE netOGlyc-4.0.0.13 CARBOHYD 16 16 0.233703 . .
SEQUENCE netOGlyc-4.0.0.13 CARBOHYD 22 22 0.484887 . .
SEQUENCE netOGlyc-4.0.0.13 CARBOHYD 27 27 0.0487009 . .
SEQUENCE netOGlyc-4.0.0.13 CARBOHYD 39 39 0.1773 . .
SEQUENCE netOGlyc-4.0.0.13 CARBOHYD 40 40 0.133288 . .
SEQUENCE netOGlyc-4.0.0.13 CARBOHYD 53 53 0.08952 . .
SEQUENCE netOGlyc-4.0.0.13 CARBOHYD 61 61 0.309658 . .
SEQUENCE netOGlyc-4.0.0.13 CARBOHYD 83 83 0.350972 . .
SEQUENCE netOGlyc-4.0.0.13 CARBOHYD 91 91 0.371068 . .
SEQUENCE netOGlyc-4.0.0.13 CARBOHYD 95 95 0.513364 . . #POSITIVE
SEQUENCE netOGlyc-4.0.0.13 CARBOHYD 104 104 0.0170542 . .
SEQUENCE netOGlyc-4.0.0.13 CARBOHYD 107 107 0.00874821 . .
SEQUENCE netOGlyc-4.0.0.13 CARBOHYD 118 118 0.116111 . .
SEQUENCE netOGlyc-4.0.0.13 CARBOHYD 121 121 0.0403274 . .
SEQUENCE netOGlyc-4.0.0.13 CARBOHYD 125 125 0.0628577 . .
SEQUENCE netOGlyc-4.0.0.13 CARBOHYD 134 134 0.151946 . .
SEQUENCE netOGlyc-4.0.0.13 CARBOHYD 140 140 0.0147821 . .
SEQUENCE netOGlyc-4.0.0.13 CARBOHYD 156 156 0.17205 . .
SEQUENCE netOGlyc-4.0.0.13 CARBOHYD 160 160 0.105781 . .
SEQUENCE netOGlyc-4.0.0.13 CARBOHYD 163 163 0.151078 . .
SEQUENCE netOGlyc-4.0.0.13 CARBOHYD 169 169 0.190072 . .
SEQUENCE netOGlyc-4.0.0.13 CARBOHYD 175 175 0.347818 . .
SEQUENCE netOGlyc-4.0.0.13 CARBOHYD 183 183 0.619488 . . #POSITIVE
SEQUENCE netOGlyc-4.0.0.13 CARBOHYD 194 194 0.657458 . . #POSITIVE
SEQUENCE netOGlyc-4.0.0.13 CARBOHYD 199 199 0.817259 . . #POSITIVE
SEQUENCE netOGlyc-4.0.0.13 CARBOHYD 204 204 0.552098 . . #POSITIVE
SEQUENCE netOGlyc-4.0.0.13 CARBOHYD 210 210 0.412808 . .
Mus musculus
C
#seqname source feature start end score strand frame comment
SEQUENCE netOGlyc-4.0.0.13 CARBOHYD 4 4 0.0726224 . .
SEQUENCE netOGlyc-4.0.0.13 CARBOHYD 9 9 0.0587432 . .
SEQUENCE netOGlyc-4.0.0.13 CARBOHYD 15 15 0.141055 . .
SEQUENCE netOGlyc-4.0.0.13 CARBOHYD 16 16 0.233703 . .
SEQUENCE netOGlyc-4.0.0.13 CARBOHYD 22 22 0.484887 . .
SEQUENCE netOGlyc-4.0.0.13 CARBOHYD 27 27 0.0487009 . .
SEQUENCE netOGlyc-4.0.0.13 CARBOHYD 39 39 0.1773 . .
SEQUENCE netOGlyc-4.0.0.13 CARBOHYD 40 40 0.133288 . .
SEQUENCE netOGlyc-4.0.0.13 CARBOHYD 53 53 0.08952 . .
SEQUENCE netOGlyc-4.0.0.13 CARBOHYD 61 61 0.309658 . .
SEQUENCE netOGlyc-4.0.0.13 CARBOHYD 83 83 0.350972 . .
SEQUENCE netOGlyc-4.0.0.13 CARBOHYD 91 91 0.371068 . .
SEQUENCE netOGlyc-4.0.0.13 CARBOHYD 95 95 0.513364 . . #POSITIVE
SEQUENCE netOGlyc-4.0.0.13 CARBOHYD 104 104 0.0170542 . .
SEQUENCE netOGlyc-4.0.0.13 CARBOHYD 107 107 0.00874821 . .
SEQUENCE netOGlyc-4.0.0.13 CARBOHYD 118 118 0.116111 . .
SEQUENCE netOGlyc-4.0.0.13 CARBOHYD 121 121 0.0403274 . .
SEQUENCE netOGlyc-4.0.0.13 CARBOHYD 125 125 0.0628577 . .
SEQUENCE netOGlyc-4.0.0.13 CARBOHYD 134 134 0.151946 . .
SEQUENCE netOGlyc-4.0.0.13 CARBOHYD 140 140 0.0147821 . .
SEQUENCE netOGlyc-4.0.0.13 CARBOHYD 156 156 0.17205 . .
SEQUENCE netOGlyc-4.0.0.13 CARBOHYD 160 160 0.105781 . .
SEQUENCE netOGlyc-4.0.0.13 CARBOHYD 163 163 0.151078 . .
SEQUENCE netOGlyc-4.0.0.13 CARBOHYD 169 169 0.190072 . .
SEQUENCE netOGlyc-4.0.0.13 CARBOHYD 175 175 0.347818 . .
SEQUENCE netOGlyc-4.0.0.13 CARBOHYD 183 183 0.619488 . . #POSITIVE
SEQUENCE netOGlyc-4.0.0.13 CARBOHYD 194 194 0.657458 . . #POSITIVE
SEQUENCE netOGlyc-4.0.0.13 CARBOHYD 199 199 0.817259 . . #POSITIVE
SEQUENCE netOGlyc-4.0.0.13 CARBOHYD 204 204 0.552098 . . #POSITIVE
SEQUENCE netOGlyc-4.0.0.13 CARBOHYD 210 210 0.412808 . .
Rattus norvegicus
D
0 Day
3Day
6 Day
9Day
Pixels (x1000)
Tone
0D
3D
6D
9D
E
TMG(μM)
0
0.625
1.25
2.5
5
10
O-GlcNAc
CTSB
β-actin
Relative protein expression of O-GlcNAC
Relative protein expression of CTSB
0μM
0.625μM
1.25μM
2.5μM
5μM
10μM
**
F
CHX(μM)
0
0.625
1.25
2.5
5
10
CTSB
β-actin
Relative protein expression of CTSB
G
MG132(nM)
0
20
100
200
CTSB
β-actin
20nM
100nM
200nM
*

Fig. S4 Co-localization of CTSB and OGT in mouse skeletal muscle tissue by immunofluorescence. (A: Immunofluorescence co-localization analysis of CTSB and OGT proteins in mouse skeletal muscle tissue overexpressing CTSB; B: Immunofluorescence co-localization analysis of CTSB and OGT proteins in mouse skeletal muscle tissue overexpressing OGT)

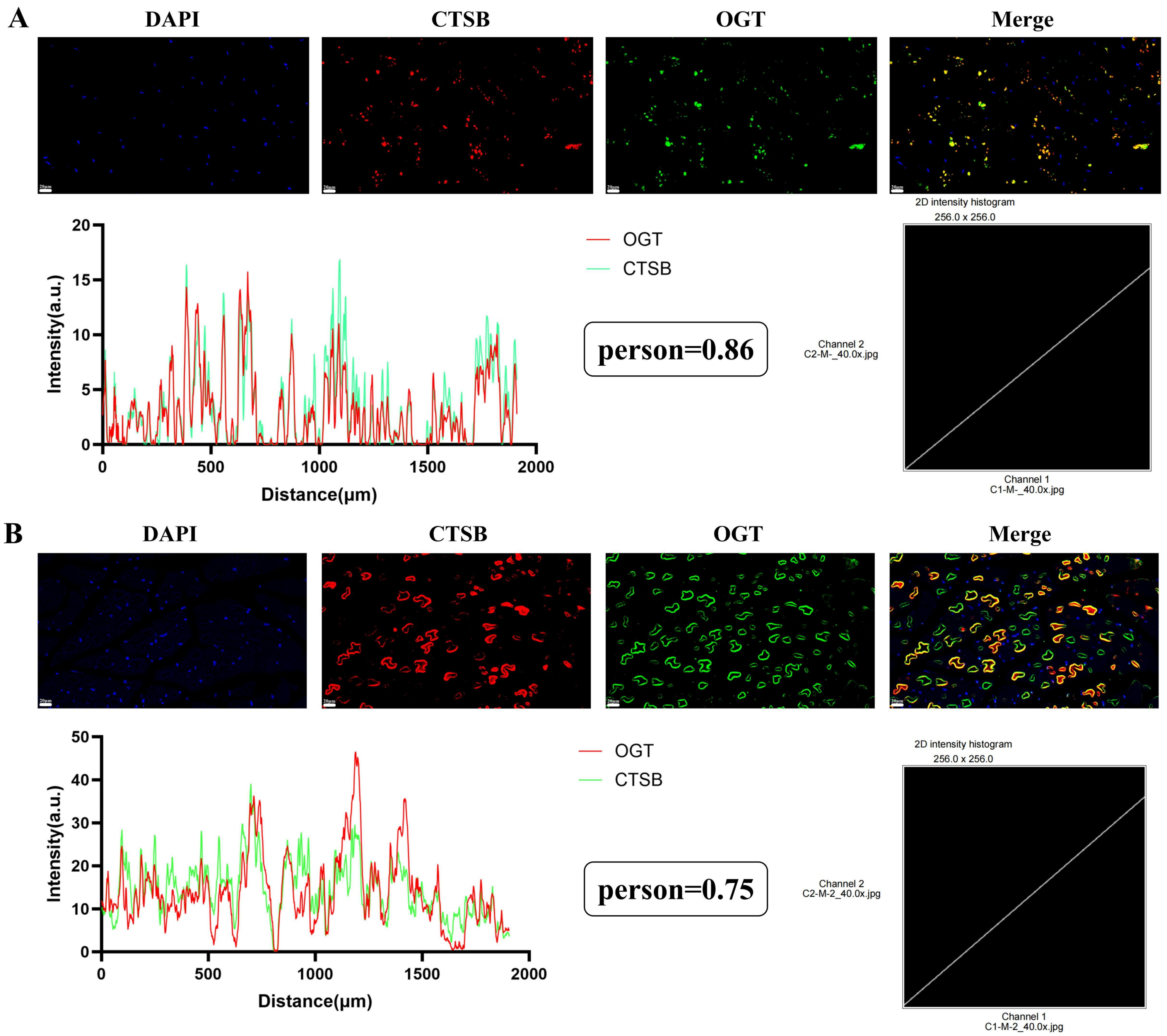
A
DAPI
CTSB
OGT
Merge
2D intensity histogram
256.0 x 256.0
OGT
CTSB
person=0.86
Intensity(a.u.)
Distance(μm)
Channel 2
C2-M-_40.0x.jpg
Channel 1
C1-M-_40.0x.jpg
B
DAPI
CTSB
OGT
Merge
2D intensity histogram
256.0 x 256.0
OGT
CTSB
person=0.75
Intensity(a.u.)
Distance(μm)
Channel 2
C2-M-2_40.0x.jpg
Channel 1
C1-M-2_40.0x.jpg

Fig. S5 Effects of knockdown or overexpression of CTSB expression in muscle tissue on pathological changes in brain tissue of APP/PS1 mice. (A: HE staining of brain tissue in APP/PS1 mice; B: Nissl staining of brain tissue in APP/PS1 mice; C: Thioflavin S staining of brain tissue in APP/PS1 mice; D: Silver staining of brain tissue in APP/PS1 mice; E: Immunohistochemical staining of GFAP protein expression in brain tissue of APP/PS1 mice; F: Immunohistochemical staining of Iba1 protein expression in brain tissue of APP/PS1 mice; G: Statistical analysis of GFAP protein expression in APP/PS1 mouse brain tissue; H: Statistical analysis of Iba1 protein expression in APP/PS1 mouse brain tissue; I: CTSB and BDNF protein expression in APP/PS1 mouse blood was measured via ELISA; n=6, *$P<0.05$, **$P<0.01$)

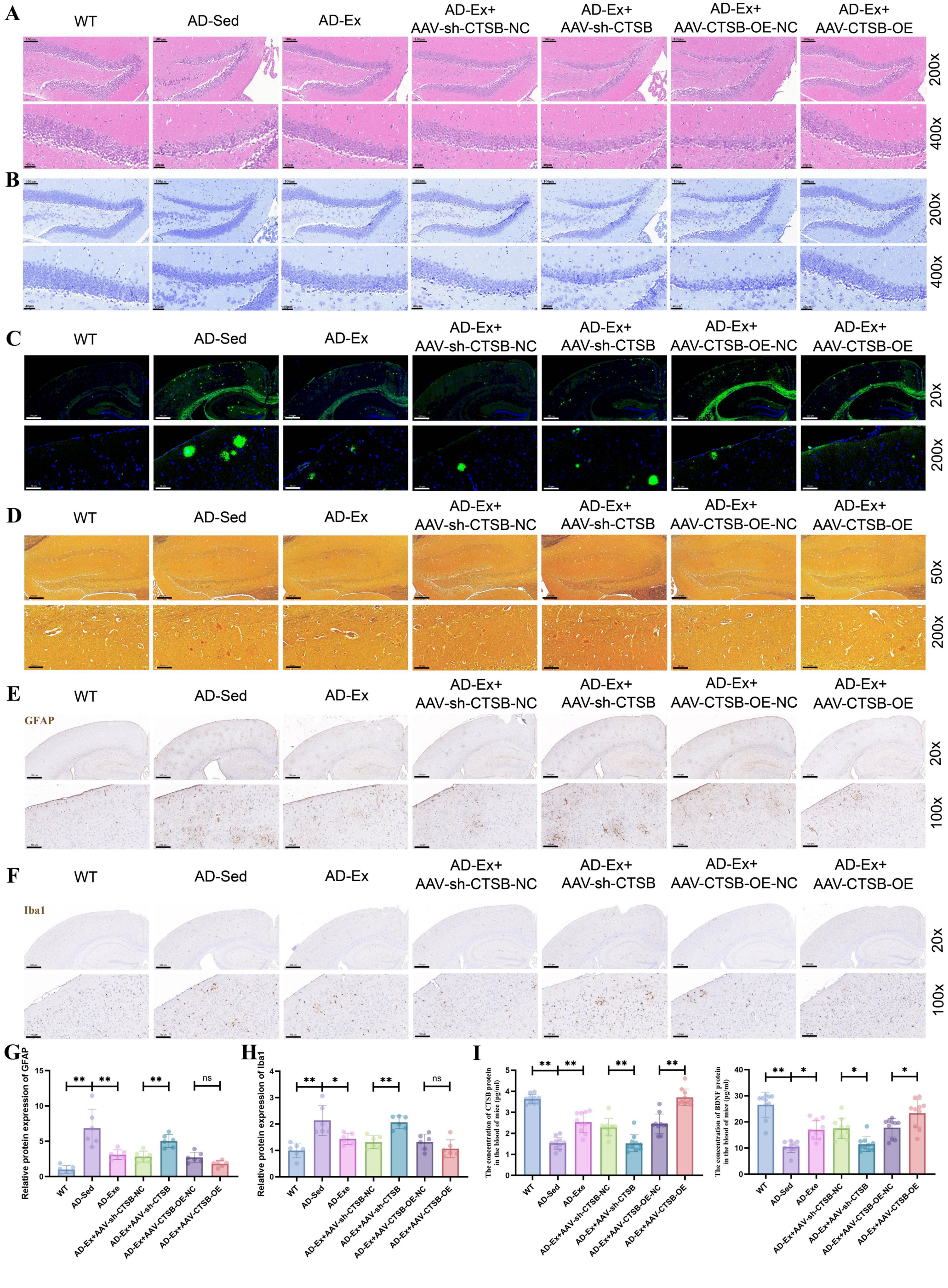
A
WT
AD-Sed
AD-Ex
AD-Ex+ AAV-sh-CTSB-NC
AD-Ex+ AAV-sh-CTSB
AD-Ex+ AAV-CTSB-OE-NC
AD-Ex+ AAV-CTSB-OE
200x
400x
B
200x
400x
C
WT
AD-Sed
AD-Ex
AD-Ex+ AAV-sh-CTSB-NC
AD-Ex+ AAV-sh-CTSB
AD-Ex+ AAV-CTSB-OE-NC
AD-Ex+ AAV-CTSB-OE
20x
200x
D
WT
AD-Sed
AD-Ex
AD-Ex+ AAV-sh-CTSB-NC
AD-Ex+ AAV-sh-CTSB
AD-Ex+ AAV-CTSB-OE-NC
AD-Ex+ AAV-CTSB-OE
50x
200x
E
GFAP
WT
AD-Sed
AD-Ex
AD-Ex+ AAV-sh-CTSB-NC
AD-Ex+ AAV-sh-CTSB
AD-Ex+ AAV-CTSB-OE-NC
AD-Ex+ AAV-CTSB-OE
20x
100x
F
Iba1
WT
AD-Sed
AD-Ex
AD-Ex+ AAV-sh-CTSB-NC
AD-Ex+ AAV-sh-CTSB
AD-Ex+ AAV-CTSB-OE-NC
AD-Ex+ AAV-CTSB-OE
20x
100x
G
Relative protein expression of GFAP
H
Relative protein expression of Iba1
I
The concentration of CTSB protein in the blood of mice (pg/ml)
The concentration of BDNF protein in the blood of mice (pg/ml)
WT
AD-Sed
AD-Exe
AD-Ex+AAV-sh-CTSB-NC
AD-Ex+AAV-sh-CTSB
AD-Ex+AAV-CTSB-OE-NC
AD-Ex+AAV-CTSB-OE
**
*
ns

Fig. S6 CTSB T199 site mutation reduces protein stability. (A-B: WB analysis of the effects of CTSB T199A mutation combined with MG132 treatment on CTSB protein expression in C2C12 cells; C and E: WB analysis of the effects of CTSB T199A mutation combined with CHX treatment on CTSB protein expression in C2C12 cells; D and F: WB analysis of the effects of CTSB T199A mutation combined with CHX/OSMI-1 treatment on CTSB protein expression in C2C12 cells; G-H: HIP assay to detect the effect of CTSB T199A mutation combined with MG132/OSMI-1 treatment on CTSB ubiquitination; n=3, *P<0.05, **P<0.01)

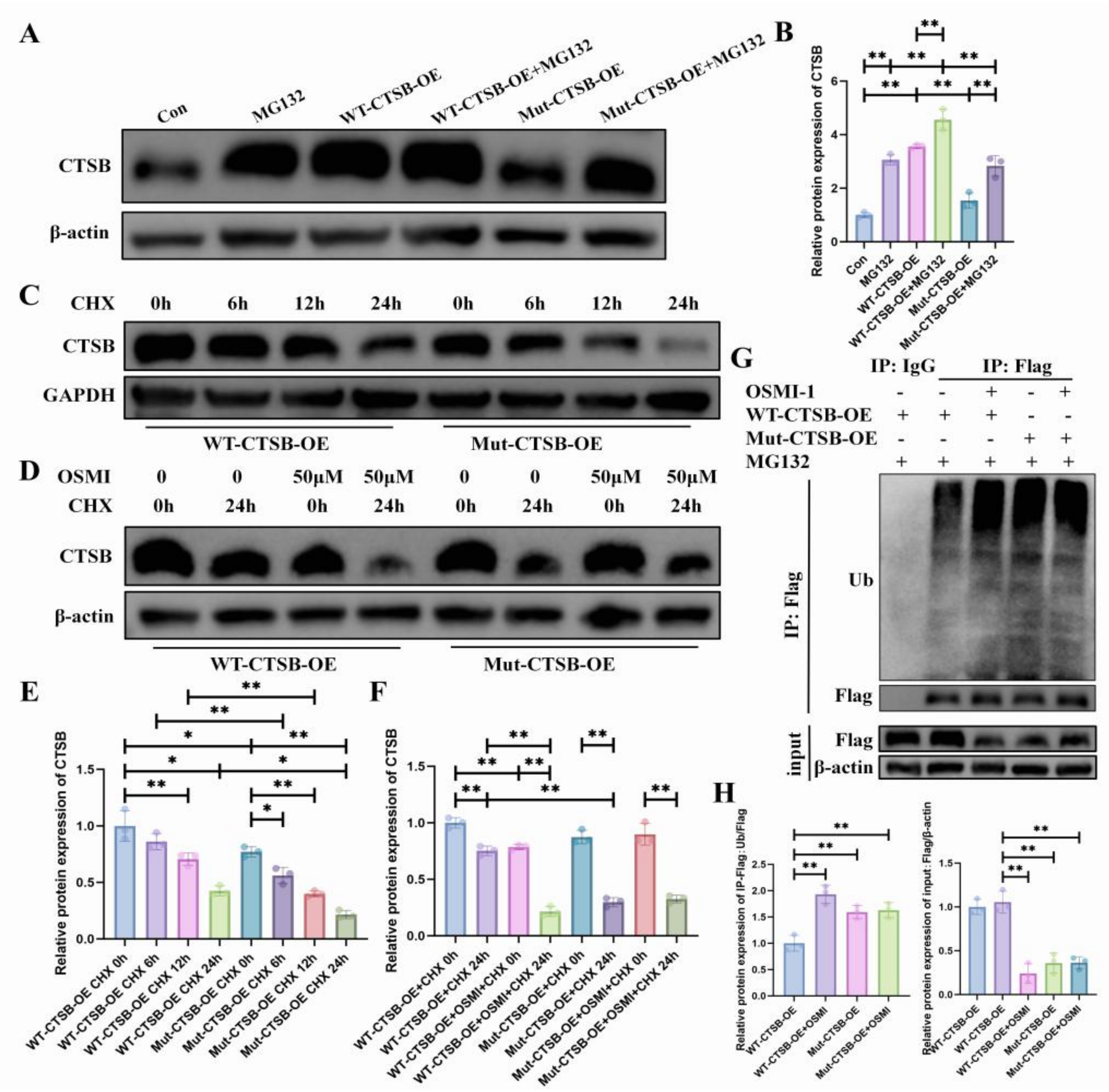
A
Con
MG132
WT-CTSB-OE
WT-CTSB-OE+MG132
Mut-CTSB-OE
Mut-CTSB-OE+MG132
CTSB
β-actin
B
Relative protein expression of CTSB
C
CHX 0h 6h 12h 24h 0h 6h 12h 24h
CTSB
GAPDH
WT-CTSB-OE
Mut-CTSB-OE
D
OSMI 0 0 50μM 50μM 0 0 50μM 50μM
CHX 0h 24h 0h 24h 0h 24h 0h 24h
CTSB
β-actin
WT-CTSB-OE
Mut-CTSB-OE
E
WT-CTSB-OE CHX 0h
WT-CTSB-OE CHX 6h
WT-CTSB-OE CHX 12h
WT-CTSB-OE CHX 24h
Mut-CTSB-OE CHX 0h
Mut-CTSB-OE CHX 6h
Mut-CTSB-OE CHX 12h
Mut-CTSB-OE CHX 24h
F
WT-CTSB-OE+CHX 0h
WT-CTSB-OE+CHX 24h
WT-CTSB-OE+OSMI+CHX 0h
WT-CTSB-OE+OSMI+CHX 24h
Mut-CTSB-OE+CHX 0h
Mut-CTSB-OE+CHX 24h
Mut-CTSB-OE+OSMI+CHX 0h
Mut-CTSB-OE+OSMI+CHX 24h
G
IP: IgG
IP: Flag
OSMI-1
WT-CTSB-OE
Mut-CTSB-OE
MG132
Ub
Flag
IP: Flag
input
β-actin
H
Relative protein expression of IP-Flag: Ub/Flag
Relative protein expression of input: Flag/β-actin
WT-CTSB-OE
WT-CTSB-OE+OSMI
Mut-CTSB-OE
Mut-CTSB-OE+OSMI

Fig. S7 Myoblast-derived EVs may ameliorate Aβ-induced hippocampal neuronal cell injury by delivering CTSB. (A: CCK-8 assay to assess the effect of different concentrations of AIACR on C2C12 cells; B: WB analysis of the effects of AIACR on total O-GlcNAcylation levels and CTSB protein expression in C2C12 cells at different time points; C: WB detection of EV protein markers; D: Nanoparticle flow cytometry analysis of the size distribution of isolated myoblast-derived EVs; E: TEM analysis of the morphology and size of isolated myoblast-derived EVs; F: IF assay to determine whether PKH26-labeled EVs were internalized by C2C12 cells; G: RT-qPCR analysis of the effects of different concentrations of myoblast-derived EVs on the expression of genes related to proliferation and synaptic plasticity in Aβ-treated HT22 cells; H: IF assay to detect the effects of different concentrations of myoblast-derived EVs on ROS accumulation in Aβ-treated HT22 cells; I: Flow cytometry analysis of the effects of different concentrations of myoblast-derived EVs on apoptosis in Aβ-treated HT22 cells; n=3, $*P<0.05$, $**P<0.01$)

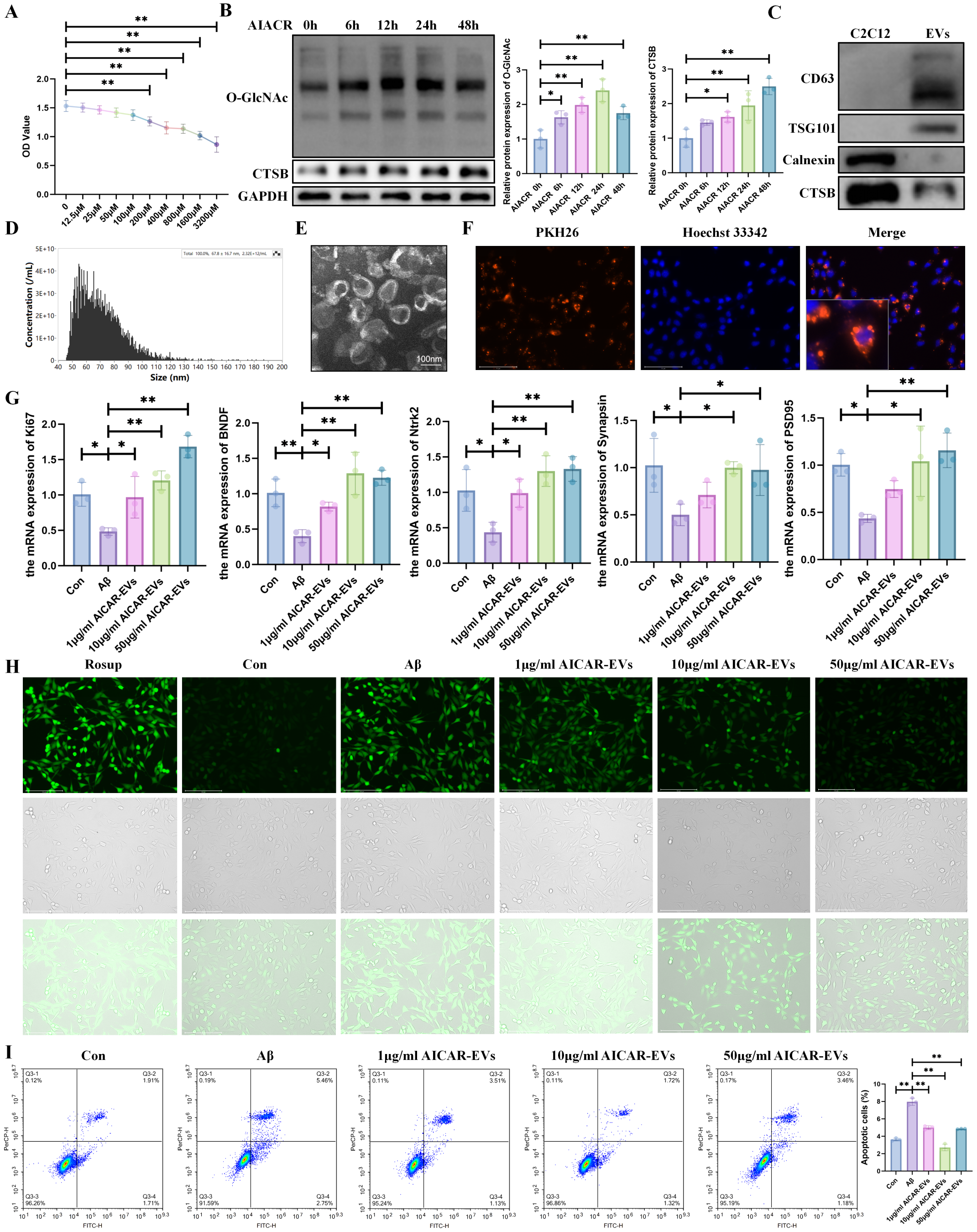
A
OD Value
0
12.5μM
25μM
50μM
100μM
200μM
400μM
800μM
1600μM
3200μM
B
AIACR 0h 6h 12h 24h 48h
O-GlcNAc
CTSB
GAPDH
Relative protein expression of O-GlcNAc
Relative protein expression of CTSB
C
C2C12
EVs
CD63
TSG101
Calnexin
CTSB
D
Concentration (/mL)
Size (nm)
E
100nm
F
PKH26
Hoechst 33342
Merge
G
the mRNA expression of Ki67
the mRNA expression of BNDF
the mRNA expression of Ntrk2
the mRNA expression of Synapsin
the mRNA expression of PSD95
Con
Aβ
1μg/ml AICAR-EVs
10μg/ml AICAR-EVs
50μg/ml AICAR-EVs
H
Rosup
Con
Aβ
1μg/ml AICAR-EVs
10μg/ml AICAR-EVs
50μg/ml AICAR-EVs
I
Con
Aβ
1μg/ml AICAR-EVs
10μg/ml AICAR-EVs
50μg/ml AICAR-EVs
PerCP-H
FITC-H
Apoptotic cells (%)

Figure S8 Quantitative analysis of Figures 4K, 4T, and 4U.(AWB analysis of changes in CTSB proteine xpression after treatment with MG132, CQ, and CHX; B: IP analysis indicating that ubiquitination-mediated degradation of CTSB protein is regulated by OSMI-1; C: Four point mutation plasmids were constructed to validate potential O-GlcNAcylation sites on the CTSB protein; n=3, *P<0.05, **P<0.01)

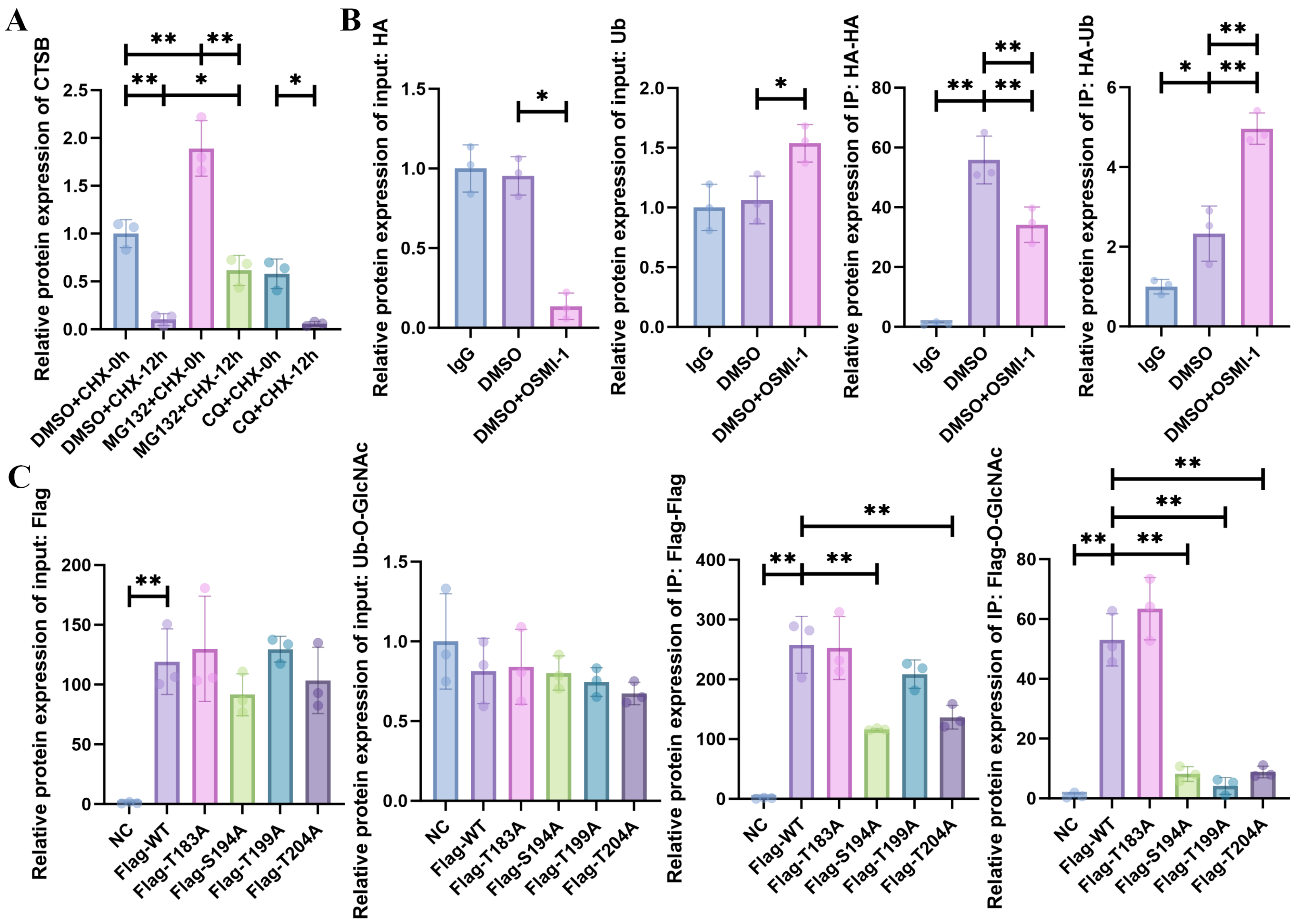

A
B
C
Relative protein expression of CTSB
DMSO+CHX-0h
DMSO+CHX-12h
MG132+CHX-0h
MG132+CHX-12h
CQ+CHX-0h
CQ+CHX-12h
Relative protein expression of input: HA
Relative protein expression of input: Ub
Relative protein expression of IP: HA-HA
Relative protein expression of IP: HA-Ub
IgG
DMSO
DMSO+OSMI-1
Relative protein expression of input: Flag
Relative protein expression of input: Ub-O-GlcNAc
Relative protein expression of IP: Flag-Flag
Relative protein expression of IP: Flag-O-GlcNAc
NC
Flag-WT
Flag-T183A
Flag-S194A
Flag-T199A
Flag-T204A

Figure S9 Quantitative analysis of BrdU$^+$/DCX$^+$, BrdU$^+$/NeuN$^+$, BrdU$^-$/NeuN$^+$, and BDNF/CTSB.(A: Effects of different treadmill exercises on BrdU$^+$/DCX$^+$ cells in the hippocampus of WT mice; B-C: Effects of different treadmill exercises on BrdU$^+$/NeuN$^+$ and BrdU$^-$/NeuN$^+$ cells in the hippocampus of WT mice; D: The effects of CTSB protein knockdown on BrdU$^+$/DCX$^+$ cells in the hippocampus of WT mice; E-F: The effects of CTSB protein knockdown on BrdU$^+$/NeuN$^+$ and BrdU$^-$/NeuN$^+$ cells in the hippocampus of WT mice; GOGT protein overexpression: The effects of on BrdU$^+$/DCX$^+$ cells in the hippocampus of WT mice; H-I: The effects of OGT protein overexpression on BrdU$^+$/NeuN$^+$ and BrdU$^-$/NeuN$^+$ cells in the hippocampus of WT mice; J: The effects of knockdown/overexpression of CTSB Protein on BrdU$^+$/DCX$^+$ cells in the hippocampus of APP/PS1 Mice; K-L: The effects of knockdown/overexpression of CTSB Protein on BrdU$^+$/NeuN$^+$ and BrdU$^-$/NeuN$^+$ cells in the hippocampus of APP/PS1 Mice; M: The effect of CTSB protein knockdown on BDNF protein expression in the hippocampus of WT mice; N: The effect of CTSB protein knockdown on CTSB protein expression in the hippocampus of WT mice; O: The effect of OGT protein overexpression on BDNF protein expression in the hippocampus of WT mice; P: The effect of OGT protein overexpression on CTSB protein expression in the hippocampus of WT mice; Q: The effects of knockdown/overexpression of CTSB Protein on BDNF protein expression in the hippocampus of APP/PS1 Mice; R: The effects of knockdown/overexpression of CTSB Protein on CTSB protein expression in the hippocampus of APP/PS1 Mice; n=6, *P<0.05, **P<0.01)

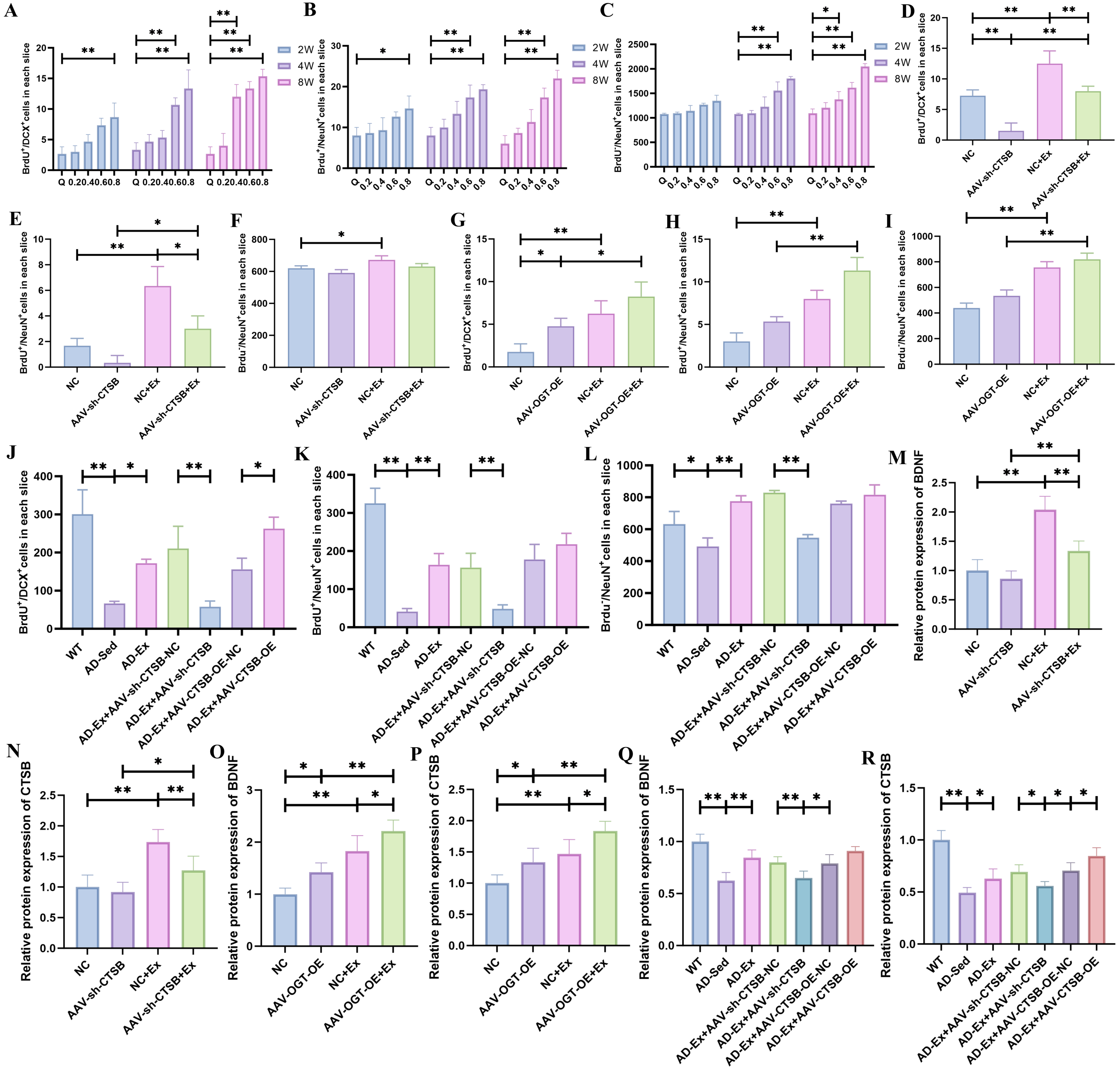
A
BrdU⁺/DCX⁺cells in each slice
2W
4W
8W
Q 0.2 0.4 0.6 0.8
B
Brdu⁺/NeuN⁺cells in each slice
C
BrdU⁺/NeuN⁺cells in each slice
D
BrdU⁺/DCX⁺cells in each slice
NC
AAV-sh-CTSB
NC+Ex
AAV-sh-CTSB+Ex
E
BrdU⁺/NeuN⁺cells in each slice
F
Brdu⁺/NeuN⁺cells in each slice
G
BrdU⁺/DCX⁺cells in each slice
AAV-OGT-OE
AAV-OGT-OE+Ex
H
Brdu⁺/NeuN⁺cells in each slice
I
Brdu⁺/NeuN⁺cells in each slice
J
BrdU⁺/DCX⁺cells in each slice
WT
AD-Sed
AD-Ex
AD-Ex+AAV-sh-CTSB-NC
AD-Ex+AAV-sh-CTSB
AD-Ex+AAV-CTSB-OE-NC
AD-Ex+AAV-CTSB-OE
K
BrdU⁺/NeuN⁺cells in each slice
L
Brdu⁺/NeuN⁺cells in each slice
M
Relative protein expression of BDNF
N
Relative protein expression of CTSB
O
Relative protein expression of BDNF
P
Relative protein expression of CTSB
Q
Relative protein expression of BDNF
R
Relative protein expression of CTSB

Figure3A: WB analysis of changes in total O-GlcNAcylation and CTSB protein expression following treatment with different concentrations of OSMI-1;

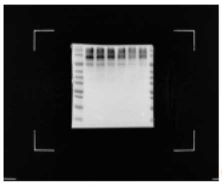
O-GlcNAc

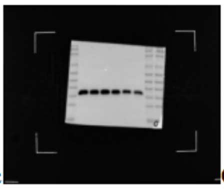
CTSB

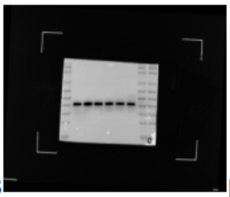
β-actin

Figure3B: WB analysis of changes in total O-GlcNAcylation, OGT, and CTSB protein expression after OGT knockdown;

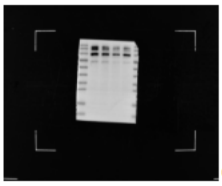
O-GlcNAc

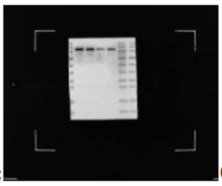
OGT

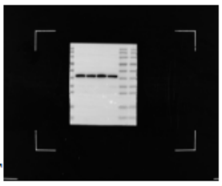
β-actin

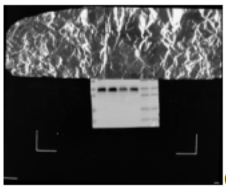
CTSB

Figure3D: WB analysis of changes in total O-GlcNAcylation, Flag-OGT, and CTSB protein expression after OGT overexpression;

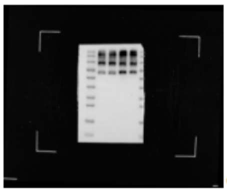
O-GlcNAc

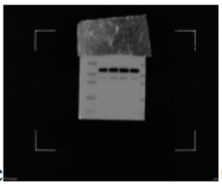
GAPDH

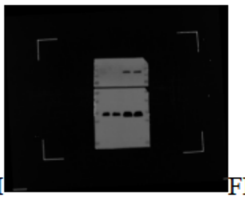
FLAG/CTSB

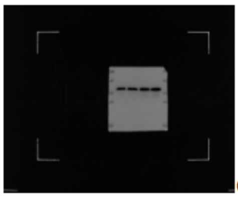
GAPDH

Figure3E: WB analysis of changes in CTSB protein expression after treatment with OSMI-1 and CHX;

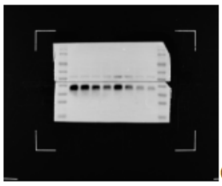
CTSB

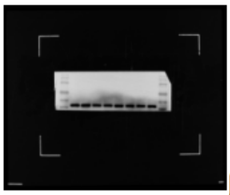
β-tubulin

Figure3F: WB analysis of changes in CTSB protein expression after treatment with si-OGT and CHX;

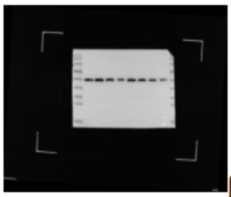
β-tubulin

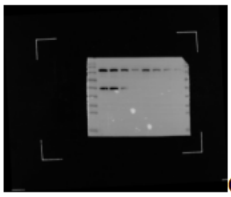
OGT

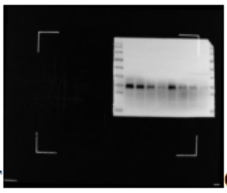
CTSB

Figure3G: WB analysis of changes in CTSB protein expression after treatment with OGT-OE and

CHX;

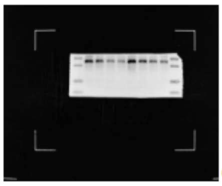
CTSB
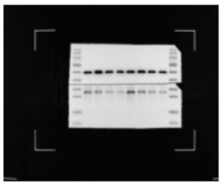
β-tubulin
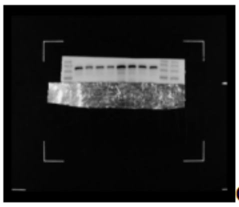
OGT

Figure3H: WB analysis of changes in CTSB protein expression after treatment with OSMI-1 and MG132;

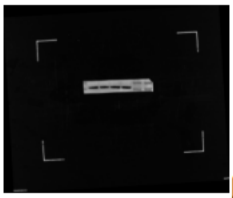
β-actin
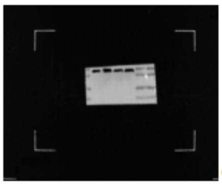
CTSB

Figure3I: WB analysis of changes in CTSB protein expression after treatment with OGT-OE and MG132;

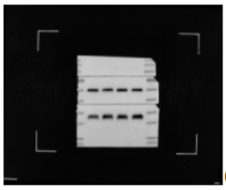
CTSB/β-actin
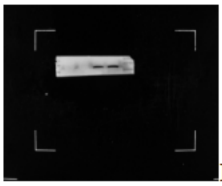
FLAG

Figure3J: WB analysis of changes in CTSB protein expression after treatment with si-OGT and MG132;

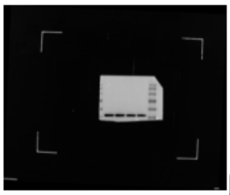
β-actin
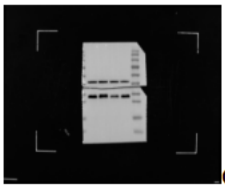
CTSB
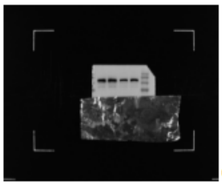
OGT

Figure3K: WB analysis of changes in CTSB protein expression after treatment with MG132, CQ, and CHX;

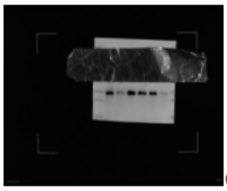
CTSB
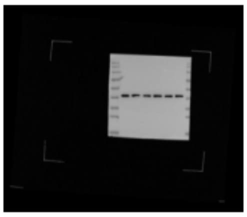
β-actin

Figure3N-O: Endogenous co-IP analysis suggesting a potential physical interaction between CTSB and OGT proteins;

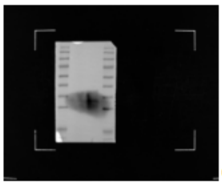
CTSB
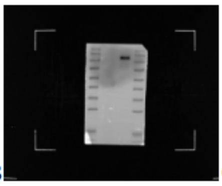
OGT
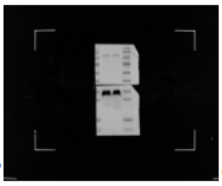
CTSB

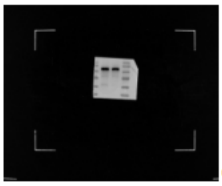
OGT
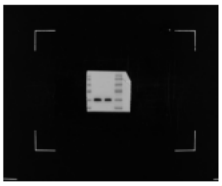
β-tubulin

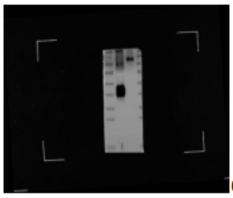
OGT
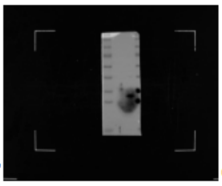
CTSB
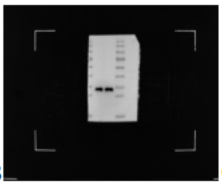
CTSB

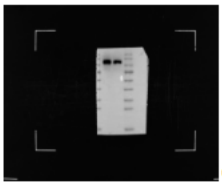
OGT
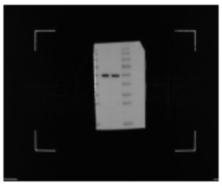
β-actin

Figure3P-Q: Exogenous co-IP analysis suggesting a potential physical interaction between CTSB and OGT proteins;

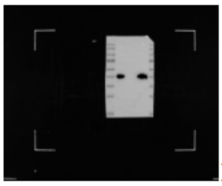
HA
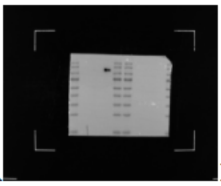
FLAG
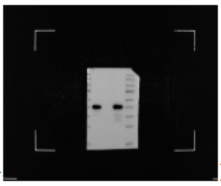
HA
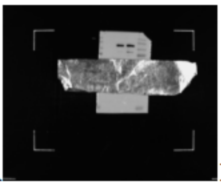
FLAG

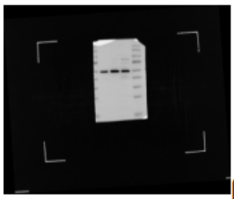
β-actin

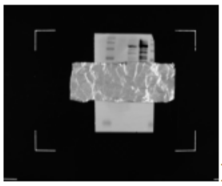
FLAG
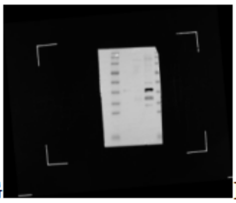
HA
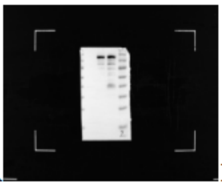
FLAG

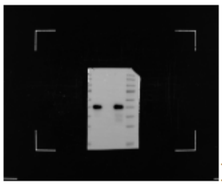
HA
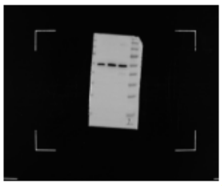
β-tubulin

Figure3R: GST pull-down assay suggesting a potential physical interaction between CTSB and OGT proteins;

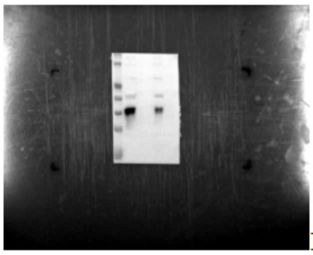
HA-CTSB
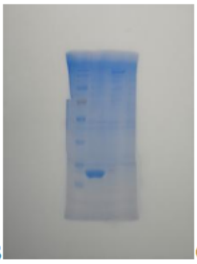
GST

Figure3S: IP analysis suggesting the presence of O-GlcNAcylation modification on CTSB protein;

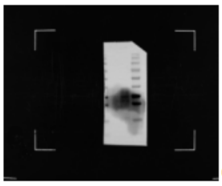
O-GlcNAc
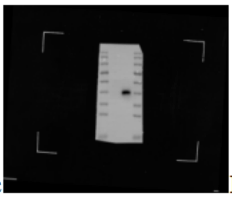
HA
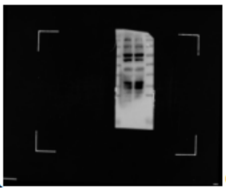
O-GlcNAc

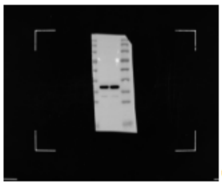
GAPDH
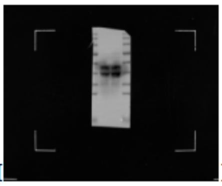
HA

Figure3T: IP analysis indicating that ubiquitination-mediated degradation of CTSB protein is regulated by OSMI-1; n=3, *P<0.05, **P<0.01)

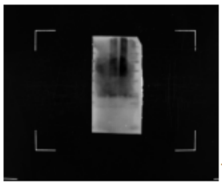
Ub
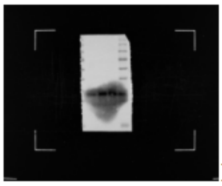
HA
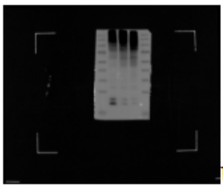
Ub
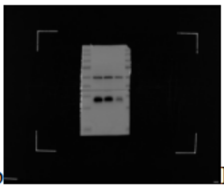
HA

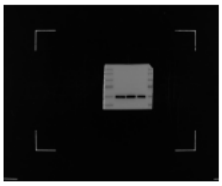
β-tubulin

Figure3U: Four point mutation plasmids were constructed to validate potential O-GlcNAcylation sites on the CTSB protein

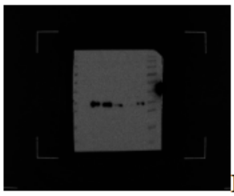
IP: O-GlcNAc
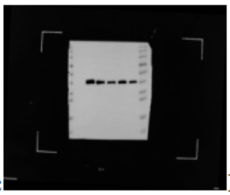
IP: Flag

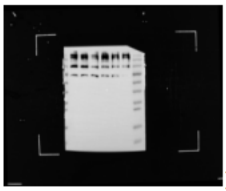
Input: O-GlcNAc
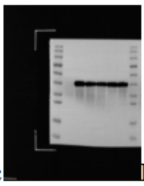
Input: Flag
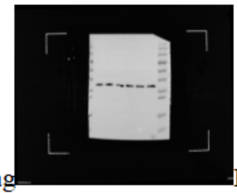
Input: GAPDH

Figure S3E: WB analysis of total O-GlcNAcylation levels and CTSB protein expression after treatment with different concentrations of TMG;

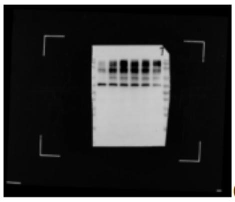
O-GlcNAc

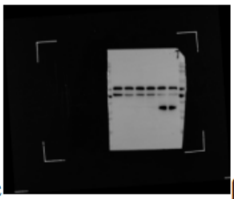
β-actin

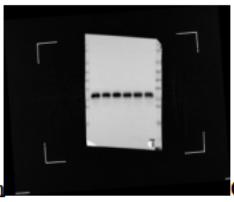
CTSB

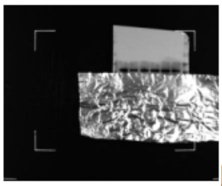
β-actin

Figure S3F: WB analysis of CTSB protein expression after treatment with different concentrations of CHX;

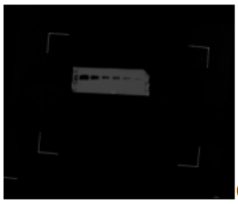
CTSB

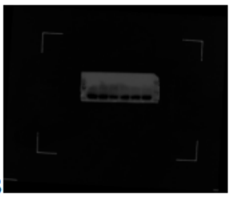
β-actin

Figure S3G: WB analysis of CTSB protein expression after treatment with different concentrations of MG132; n=3, *P<0.05, **P<0.01)

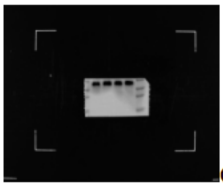
CTSB

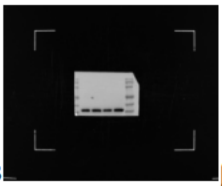
β-actin

Figure4K: WB analysis of CTSB protein expression in skeletal muscle tissue of WT mice

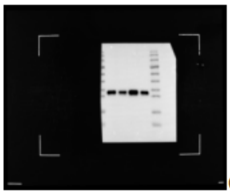
CTSB
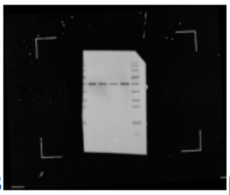
β-Tubulin

Figure5L: WB analysis of total O-GlcNAcylation, OGT, and CTSB protein expression in skeletal muscle tissue of WT mice

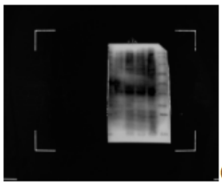
O-GlcNAc
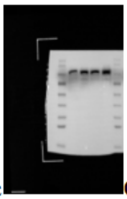
OGT
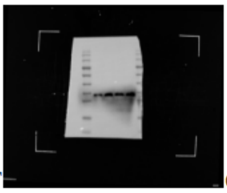
CTSB
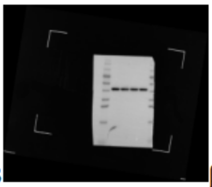
β-Tubulin

Figure7A: Protein expression of CTSB in muscle tissue of APP/PS1 mice

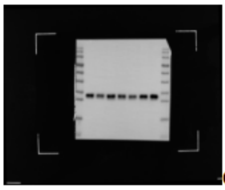
CTSB
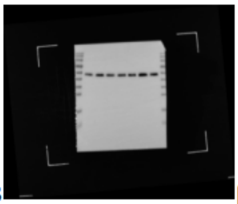
β-Tubulin

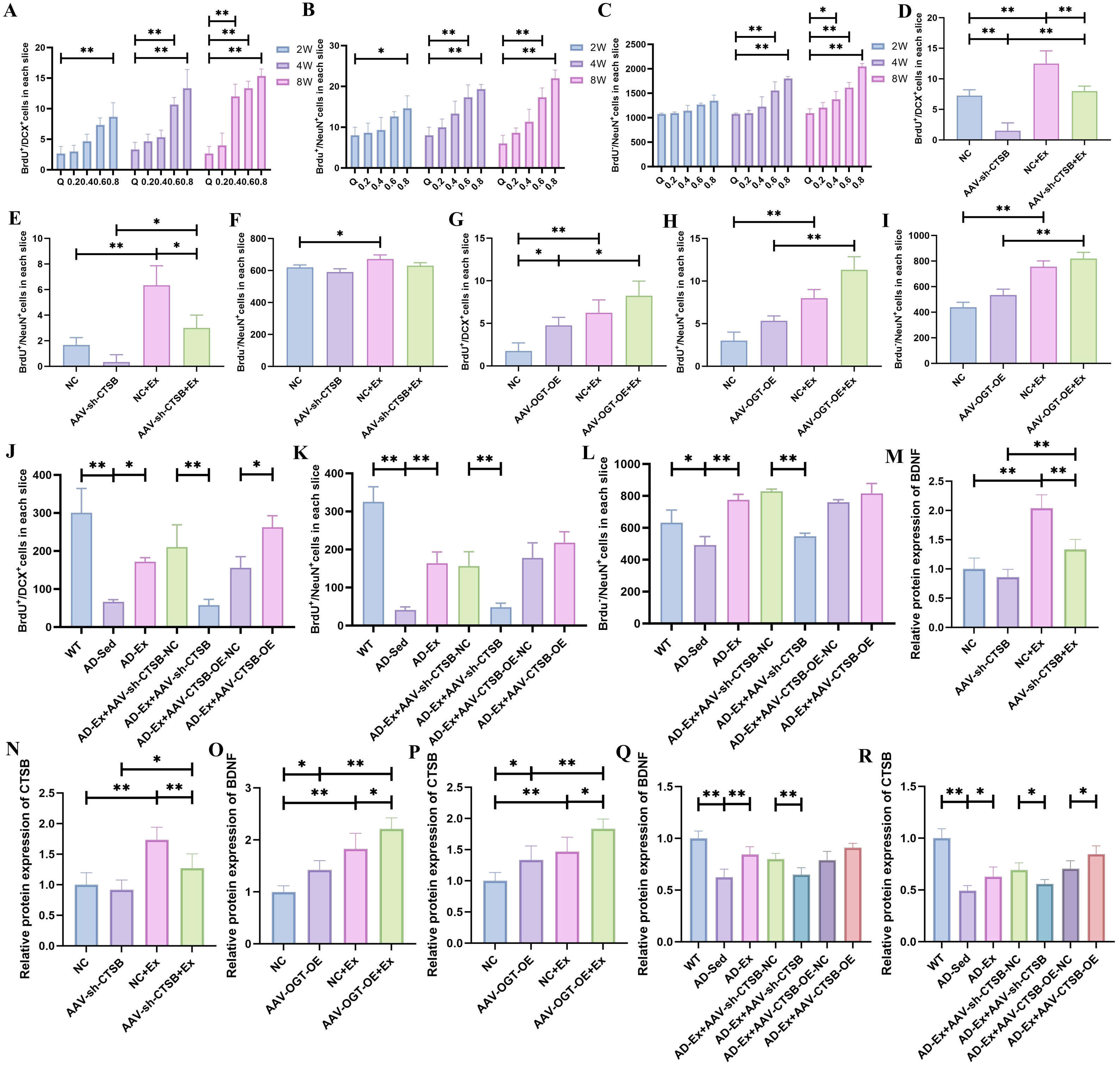
A
BrdU+/DCX+cells in each slice
Q 0.20.40.60.8
2W
4W
8W
B
Brdu+/NeuN+cells in each slice
Q 0.2 0.4 0.6 0.8
C
BrdU+/NeuN+cells in each slice
D
BrdU+/DCX+cells in each slice
NC
AAV-sh-CTSB
NC+Ex
AAV-sh-CTSB+Ex
E
BrdU+/NeuN+cells in each slice
F
Brdu+/NeuN+cells in each slice
G
BrdU+/DCX+cells in each slice
AAV-OGT-OE
AAV-OGT-OE+Ex
H
BrdU+/NeuN+cells in each slice
I
Brdu+/NeuN+cells in each slice
J
BrdU+/DCX+cells in each slice
WT
AD-Sed
AD-Ex
AD-Ex+AAV-sh-CTSB-NC
AD-Ex+AAV-sh-CTSB
AD-Ex+AAV-CTSB-OE-NC
AD-Ex+AAV-CTSB-OE
K
BrdU+/NeuN+cells in each slice
L
Brdu+/NeuN+cells in each slice
M
Relative protein expression of BDNF
N
Relative protein expression of CTSB
O
Relative protein expression of BDNF
P
Relative protein expression of CTSB
Q
Relative protein expression of BDNF
R
Relative protein expression of CTSB
