# Supplementary figures and images for: Myokine Cathepsin B as a Key Muscle–Brain Axis Regulator Mediates Treadmill-Running-Induced Hippocampal Neurogenesis and Cognitive Improvement in Mice
Source: Research (Wash D C). 2026 Apr 23;9:1233. doi: 10.34133/research.1233 (PMC13103465; doi:10.34133/research.1233)

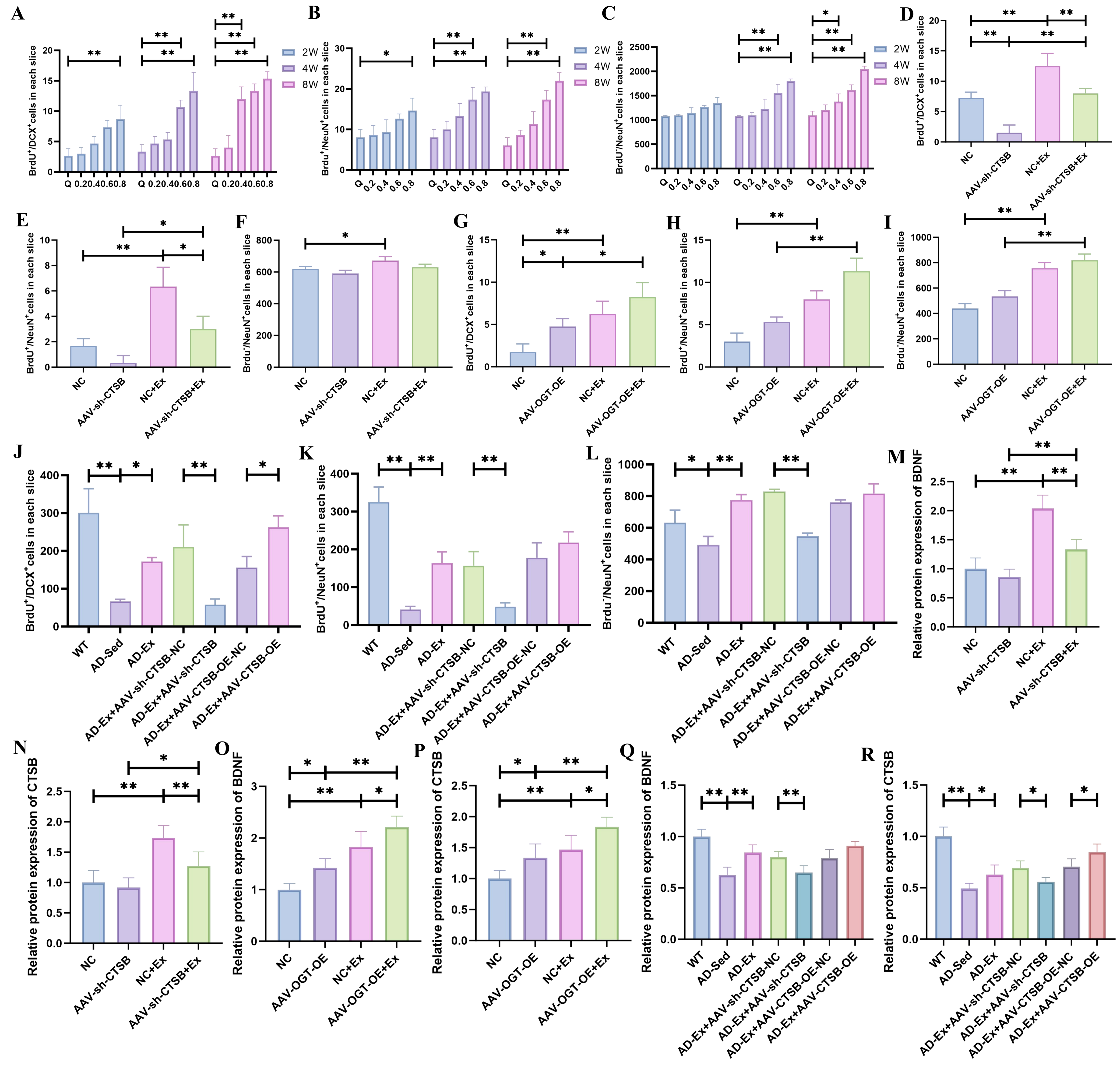

Supplement: Supplementary 1 — Supplementary Text Figs. S1 to S9 Raw Data of WB [file research.1233.f1.zip › Figure S9.pdf]
